# Supplementary material for: Routes to diagnosis of symptomatic cancer in sub-Saharan Africa: systematic review
Source: BMJ Open. 2020 Nov 19;10(11):e038605. doi: 10.1136/bmjopen-2020-038605 (PMC7678384; doi:10.1136/bmjopen-2020-038605)
Supplement: Supplementary data [file bmjopen-2020-038605supp002.pdf]

Additional file 2: Ovid MEDLINE(R), PsycINFO, Embase, Global Health

Set	Search Statement
1.	Angola*.ti,ab.
2.	Gabon*.ti,ab.
3.	Nigeria*.ti,ab.
4.	Benin*.ti,ab.
5.	Gambia*.ti,ab.
6.	Rwanda*.ti,ab.
7.	Botswana*.ti,ab.
8.	Ghana*.ti,ab.
9.	Sao Tome*.ti,ab.
10.	Sao Tome.mp. and Principe*.ti,ab. [mp=title, abstract, heading word, drug trade name, original title, device manufacturer, drug manufacturer, device trade name, keyword, floating subheading word, candidate term word]
11.	Burkina Faso*.ti,ab.
12.	Guinea-Bissau*.ti,ab.
13.	Seychelles*.ti,ab.
14.	Guinea*.ti,ab.
15.	Senegal*.ti,ab.
16.	Burundi*.ti,ab.
17.	Cabo Verde*.ti,ab.
18.	Kenya*.ti,ab.
19.	Sierra Leone*.ti,ab.
20.	Cameroon*.ti,ab.
21.	Lesotho*.ti,ab.
22.	Somalia*.ti,ab.
23.	Central African Republic*.ti,ab.
24.	Liberia*.ti,ab.
25.	South Africa*.ti,ab.
26.	Chad*.ti,ab.
27.	Madagascar*.ti,ab.
28.	South Sudan*.ti,ab.
29.	Comoros*.ti,ab.
30.	Malawi*.ti,ab.
31.	Sudan*.ti,ab.
32.	Congo*.ti,ab.
33.	Mali*.ti,ab.
34.	Swaziland*.ti,ab.
35.	Mauritania*.ti,ab.
36.	Tanzania*.ti,ab.
37.	Cote d'Ivoire*.ti,ab.
38.	Mauritius*.ti,ab.
39.	togo*.ti,ab.
40.	Equatorial Guinea*.ti,ab.
41.	Mozambique*.ti,ab.
42.	Uganda*.ti,ab.
43.	Eritrea*.ti,ab.
44.	Namibia*.ti,ab.
45.	Zambia*.ti,ab.
46.	Ethiopia*.ti,ab.
47.	Niger*.ti,ab.
48.	Zimbabwe*.ti,ab.
49.	Africa*.ti,ab.

50.	Sub-Sahara*.ti,ab.
51.	1 or 2 or 3 or 4 or 5 or 6 or 7 or 8 or 9 or 10 or 11 or 12 or 13 or 14 or 15 or 16 or 17 or 18 or 19 or 20 or 21 or 22 or 23 or 24 or 25 or 26 or 27 or 28 or 29 or 30 or 31 or 32 or 33 or 34 or 35 or 36 or 37 or 38 or 39 or 40 or 41 or 42 or 43 or 44 or 45 or 46 or 47 or 48 or 49 or 50
52.	cancer*.ti,ab.
53.	exp Neoplasms/
54.	(adenocarcinoma or astrocytoma or glioma or Hodgkin$ or leuk?emia or lymphoma or medulloblastoma or melanoma or mesothelioma or myeloma or neuroblastoma or nonmelanoma or osteosarcoma or retinoblastoma or sarcoma or seminoma or teratoma).ti,ab.
55.	52 or 53 or 54
56.	(pathway adj5 diagnos$).ti,ab.
57.	(pathway adj5 detect$).ti,ab.
58.	(route adj5 diagnos$).ti,ab.
59.	(route adj5 detect$).ti,ab.
60.	diagnos$.ti,ab.
61.	detect$.ti,ab.
62.	consult$.ti,ab.
63.	(help adj5 seek$).ti,ab.
64.	present$.ti,ab.
65.	(route adj5 consult$).ti,ab.
66.	(route adj5 present$).ti,ab.
67.	(pathway adj5 consult$).ti,ab.
68.	(pathway adj5 present$).ti,ab.
69.	56 or 57 or 58 or 59 or 60 or 61 or 62 or 63 or 64 or 65 or 66 or 67 or 68
70.	primary care.ti,ab.
71.	family doctor.ti,ab.
72.	physician.ti,ab.
73.	(health adj5 practitioner).ti,ab.
74.	General Practitioners/ or Family Practice/ or Primary Health Care/
75.	70 or 71 or 72 or 73 or 74
76.	51 and 55 and 69 and 75
