# Supplementary material for: Routes to diagnosis of symptomatic cancer in sub-Saharan Africa: systematic review
Source: BMJ Open. 2020 Nov 19;10(11):e038605. doi: 10.1136/bmjopen-2020-038605 (PMC7678384; doi:10.1136/bmjopen-2020-038605)
Supplement: Author's manuscript [file bmjopen-2020-038605.draft_revisions.pdf]

BMJ Open is committed to open peer review. As part of this commitment we make the peer review history of every article we publish publicly available.

When an article is published we post the peer reviewers' comments and the authors' responses online. We also post the versions of the paper that were used during peer review. These are the versions that the peer review comments apply to.

The versions of the paper that follow are the versions that were submitted during the peer review process. They are not the versions of record or the final published versions. They should not be cited or distributed as the published version of this manuscript.

BMJ Open is an open access journal and the full, final, typeset and author-corrected version of record of the manuscript is available on our site with no access controls, subscription charges or pay-per-view fees (http://bmjopen.bmj.com).

If you have any questions on BMJ Open's open peer review process please email info.bmjopen@bmj.com

BMJ Open

Routes to diagnosis of symptomatic cancer in Sub-Saharan Africa: a systematic review

Journal:	*BMJ Open*
Manuscript ID	bmjopen-2020-038605
Article Type:	Original research
Date Submitted by the Author:	17-Mar-2020

Complete List of Authors:	Martins, Tanimola; University of Exeter, Primary Care; University of Exeter , Primary Care<br>Hamilton, Willie; University of Exeter Medical School, Primary Care Diagnostics

Keywords:	PRIMARY CARE, PUBLIC HEALTH, Epidemiology < ONCOLOGY, International health services < HEALTH SERVICES ADMINISTRATION & MANAGEMENT

SCHOLARONE™ Manuscripts

I, the Submitting Author has the right to grant and does grant on behalf of all authors of the Work (as defined in the below author licence), an exclusive licence and/or a non-exclusive licence for contributions from authors who are: i) UK Crown employees; ii) where BMJ has agreed a CC-BY licence shall apply, and/or iii) in accordance with the terms applicable for US Federal Government officers or employees acting as part of their official duties; on a worldwide, perpetual, irrevocable, royalty-free basis to BMJ Publishing Group Ltd ("BMJ") its licensees and where the relevant Journal is co-owned by BMJ to the co-owners of the Journal, to publish the Work in this journal and any other BMJ products and to exploit all rights, as set out in our licence.

The Submitting Author accepts and understands that any supply made under these terms is made by BMJ to the Submitting Author unless you are acting as an employee on behalf of your employer or a postgraduate student of an affiliated institution which is paying any applicable article publishing charge ("APC") for Open Access articles. Where the Submitting Author wishes to make the Work available on an Open Access basis (and intends to pay the relevant APC), the terms of reuse of such Open Access shall be governed by a Creative Commons licence – details of these licences and which Creative Commons licence will apply to this Work are set out in our licence referred to above.

Other than as permitted in any relevant BMJ Author's Self Archiving Policies, I confirm this Work has not been accepted for publication elsewhere, is not being considered for publication elsewhere and does not duplicate material already published. I confirm all authors consent to publication of this Work and authorise the granting of this licence.

For peer review only

Routes to diagnosis of symptomatic cancer in Sub-Saharan Africa: a systematic review

Tanimola Martins *, Postdoctoral Research Fellow [1]

William Hamilton, Professor of Primary care Diagnostics [1]

[1]University of Exeter Medical School, College House St Luke's Campus, Magdalen Road, Exeter, EX1 2LU

*Correspondence author's email: tom207@exeter.ac.uk

For peer review only

For peer review only - http://bmjopen.bmj.com/site/about/guidelines.xhtml

Abstract

Background: The incidence and mortality rates from cancer are rising in Sub-Saharan Africa. While effective public health measures used in developed counties can help minimise cancer risks, targeted and more radical approaches will be required to reduce cancer mortality in the region. The present study examined the evidence regarding the routes to diagnosis of cancer in Sub-Saharan Africa.

Design and settings: A systematic review of available literature was performed

Methods: The PRISMA guidelines were followed. Seven electronic databases were searched using terms relating to Sub-Saharan African countries, cancer and routes to diagnosis; comprising the population, exposure, and outcomes, respectively. Citation lists of included studies were manually searched to identify relevant studies. Furthermore, ProQuest Dissertations & Theses Global was searched to identify appropriate grey literature on the subject.

Results: 20 of 5,083 references identified met the inclusion criteria, eight focused on breast cancer, five focused on cervical cancer (two of which piloted low-cost screening methods), one on colorectal cancer, and two each focused on lymphoma, Kaposi's sarcoma, and childhood cancers. With the exception of Kaposi's sarcoma, definitive diagnoses of cases were made in tertiary health centres including (teaching and regional hospitals). The majority of participants in the review initially consulted within primary care, although a considerable proportion first used alternative medicine before seeking conventional medical help. The quality of our final selection was a major concern but their findings provide important insight into the routes to cancer diagnosis in the region.

Conclusion: Government and health departments in Sub-Saharan Africa must find radical solutions to the rising burden of cancer in the region. Interventions aimed at promoting early symptomatic presentation may improve outcomes. However, this may require better integration of various aspects of their health care systems, and recognising the role of alternative health practitioners in patients' journey to diagnosis of cancer.

For peer review only - http://bmjopen.bmj.com/site/about/guidelines.xhtml

Strengths and limitations of this study

- This is the first systematic review of the evidence relating to the routes to diagnosis of cancer in Sub-Saharan Africa
- The search strategies, assessment of quality, and narrative synthesis followed best practice
- Selected studies raised methodological concerns, but their findings provide unique insights into patients' journey to cancer diagnosis in Sub-Saharan Africa.

For peer review only

For peer review only - http://bmjopen.bmj.com/site/about/guidelines.xhtml

Background

Sub-Saharan Africa (SSA) is overburdened with communicable diseases, yet the incidence and mortality from non-communicable diseases such as cancer are rising across the region.[1] In 2018, an estimated 1.1million new cases and over 600,000 deaths due to cancer were reported in Africa, despite the absence of accurate data and significant underreporting.[2-4] These figures are considerably higher than the estimates for 2012,[5] and are expected to double by 2040, alongside the attendant social and economic costs.[3] The increase in cancer incidence is due, in part, to the poor control of cancer-related infections such as Helicobacter pylori, herpes, hepatitis, human papillomavirus (HPV) and human immunodeficiency virus (HIV). [6 7] It may also be linked to the rapid population growth, increasing life-expectancy, and unhealthy lifestyle choices, some of which may be addressed by applying lessons learned from countries in Europe and North America.[7 8] For instance, public health interventions including immunization against cancer-causing infections, tobacco and alcohol regulations, healthy eating and active lifestyle may help minimise cancer risks.[7 9 10]

Mortality from cancer is strongly associated with stage at diagnosis.[11 12] In SSA, most cancers are diagnosed at advanced stages, with limited treatment options and diminished chances of survival.[12] Part of this may be linked to the lack of organised cancer screening and several other factors relating to the patient, and health care systems organisation and operation. Patient factors such as poverty, lack of education, poor awareness of cancer, fear, stigma, and certain cultural and religious beliefs - which are strongly associated with symptoms non-recognition/misattribution and delayed medical help-seeking - are widespread in SSA.[4 11-15]

However, for the majority of patients seeking medical help, a definitive diagnosis of cancer requires specialist investigations, which in most SSA countries are limited to a few tertiary health care centres (including teaching, state and national hospitals), often located many miles away from the patient.[13] Referral to such tertiary health centres can be made by physicians in primary care or secondary care (government or privately-owned) after symptomatic presentation, although patient may access

For peer review only - http://bmjopen.bmj.com/site/about/guidelines.xhtml

specialist care directly in emergencies. This seemingly simple navigation chain is complicated by poor infrastructure, limited funding, lack of communication between health institutions, poor physician-to-patient ratio, and out-of-pocket recompense, all of which typifies health care systems in SSA.[16-19] In these health systems, the role of primary care physicians is not always well-defined, with several unorthodox providers including traditional healers, drug sellers and faith clinics offering a range of services, albeit unqualified to diagnose cancer or refer patient for specialist investigation.[16 17 20 21] These health system-related factors place patient in precarious situations with the eventual outcome being advanced-stage cancer, resulting from delayed diagnosis, underdiagnoses or misdiagnosis.[16]

Given the social and economic impacts of cancer, compounded by the struggle to cope with communicable diseases in SSA, there is need for urgent actions from government and health departments to improve cancer outcomes in the region. Such actions must be underpinned by empirical evidence to maximise resource use. In the present study, we identified and characterised the evidence relating to the routes to cancer diagnosis in SSA. Identifying and categorising routes to cancer diagnosis may help explain advanced-stage diagnosis and provide the basis for possible interventions to promote earlier diagnosis in the region.

Method

A systematic narrative review was performed. The conduct and reporting of the review was based on the Preferred Reporting Items for Systematic Reviews and Meta-Analysis (PRISMA) framework [see Additional file 1].[22]

Search Strategy

Between September 30th and November 30th 2019, a systematic search of the following electronic databases was performed: Ovid MEDLINE(R) ALL (1946 to September 30, 2019), Embase (1974 to 2019

For peer review only - http://bmjopen.bmj.com/site/about/guidelines.xhtml

For peer review only

September 30), Web of Science (1915 (1) - 2019 (69)), PsycINFO (1806 to September Week 2 2019), CINAHL Complete, Global Health (1973 to 2019 Week 36) and African Journals Online (AJOL). The search strategy included terms, their synonyms and MeSH terms relating to SSA countries, cancer, and routes to diagnosis; comprising the population, exposure and outcomes, respectively (Table 1). Citation lists of included studies were manually searched to identify relevant studies. Furthermore, ProQuest Dissertations & Theses Global was searched to identify appropriate grey literature on the subject.

Inclusion and exclusion criteria

Included studies:

- investigated cancer diagnosis
- described the routes or patient's pathway to diagnosis, including the settings of initial consultation and definitive diagnosis
- were conducted in one or more of the 48 SSA countries. Our list of SSA countries matches those featured on World Bank data catalogue used to describe health and socio-economic indices (including life-expectancy, poverty headcount, and school enrolment) in the region.[23]

Excluded studies:

- were non-English studies
- focused on populations outside the region of SSA
- investigated diseases other than cancer
- examined cancer treatment and outcomes (mortality and survival)
- examined providers' views and attitudes toward cancer diagnosis

Study Selection

This involved a two-stage screening process. Firstly, title, abstract and full articles of potentially eligible studies were sequentially screened by an experienced researcher (TM) against the inclusion and

For peer review only

exclusion criteria. Consequently, studies that appeared to meet the inclusion criteria or where a decision could not be made based on the title and/or abstract were selected for full-text review to identify those for the final analysis.

Data Extraction and synthesis

One reviewer (TM) extracted data from all included studies. Extracted data were added to a data extraction spreadsheet, which was initially piloted with seven studies. Data extraction included study characteristics: country of study, design, participants' characteristics, cancer type, health care settings for initial consultation, and eventual diagnosis. Quantitative synthesis was not possible because our final selection differed in terms of cancer sites and outcome measures. For instance, some studies described patients initially presenting to "health care practitioner", a term that may be used to describe primary care physicians or doctors in secondary care. Therefore, we performed a narrative synthesis, using the framework of Rodgers and colleagues.[24] Participants' characteristics and study main findings are illustrated in tables and figures. The predominant themes across studies were identified and form the basis of the narrative review.

Quality assessment

Two reviewers (TM and WH) assessed the methodological quality of eligible studies using the Critical Appraisal Skills Programme (CASP) checklists.[25] The CASP questions were interpreted to fit our study topic (see rows 1 and 2 in Table 3 for details). The CASP is simple, widely used, and is available for various study designs included in the review.[26] The checklist contains multiple choice questions relating to the validity of studies, significance of the study results, and their application to the research needs. TM and WH selected the appropriate CASP checklist based on study designs. Subsequently, both reviewers independently appraised and rated selected studies as "satisfactory", "medium" or

For peer review only - http://bmjopen.bmj.com/site/about/guidelines.xhtml

"high-quality", based on the extent to which the checklist items are met. Discrepancies were resolved by consensus, although no study was excluded based on quality.

Patient and Public Involvement

There was no formal patient and public involvement in this review.

Result

Study characteristics

The search identified 5,083 articles. After screening title and abstract, and removing duplicates, 4,933 irrelevant articles were excluded, 150 full text articles were assessed with 20 meeting the inclusion criteria. A PRISMA flowchart showing the reasons for abstract and full article exclusions is shown in Figure 1. The 20 studies recruited a total of 5,847 participants from eleven SSA countries, 75% of which were females with average age ranging from 4 to 59 years. The characteristics of included studies are illustrated in Table 2 with the results of quality assessment in Table 3. Seven of the 20 studies were conducted in Nigeria,[27-33] three in Ethiopia,[34-36] and two each in Ghana,[37 38] and South Africa.[39 40] One each in Cameroon,[41] Tanzania,[42] Kenya,[43] Madagascar,[44] and Democratic Republic of Congo.[45] The final study involved five countries (Kenya, Uganda, Malawi, Cameroon and Nigeria).[46]

All 20 studies were observational with seven cross-sectional surveys, seven cohorts (using medical records), three qualitative (face-to-face interviews), two pilot studies and a mixed-methods study (using both qualitative and quantitative data). Eight studies examined breast cancer,[27-30 34 35 37 38] five focused on cervical cancer (two of which piloted low cost screening methods),[33 36 42 44 45] one on colorectal cancer,[32] and two each focused on lymphoma,[40 41] Kaposi's sarcoma,[39 46] and childhood cancers.[31 43] None of the 20 studies specifically investigated the routes to cancer diagnosis, though 17 studies reported the settings of initial consultation after symptoms onset. The remaining three studies recruited participants from primary care-based HIV clinics to investigate Kaposi's sarcoma and lymphoma diagnoses.[39 40 46] These studies were included in our final selection given that both cancer

For peer review only - http://bmjopen.bmj.com/site/about/guidelines.xhtml

types are significantly more common in HIV patients and that HIV patients would most likely be seen at such settings.

Assessment of study quality

Overall, none of our 20 eligible studies had fewer than two concerns and none could be classified as "medium or high quality" study due to the limitations in their methodology (Table 3). The main flaws in these studies pertained to their small sample sizes, biases in participant recruitment, and data collection strategies. The sample sizes in most of the cohort and cross-sectional studies were rather small to be representative of the target population. 17 of the 20 studies recruited participants from hospitals, thereby introducing selection bias by systematically excluding patients diagnosed or treated elsewhere. In some studies, surveys and face-to-face interviews were performed by nurses or physician-researchers from the hospitals where participants were receiving treatment, thus drawing possibly desirable responses. Additionally, statistical analysis was largely descriptive, with most studies presenting percentages only. Despite these flaws, however, the studies provided some important findings relevant to the aim of our review, thereby warranting their inclusion in the synthesis.

Routes to cancer diagnosis

Breast cancer

Across the eight study that focused on breast cancer, definitive diagnoses of all cases were made at the tertiary health centres (Table 2). [27-30 34 35 37 38] After noticing symptoms, participants initially consulted the physician, used alternative medicine (including traditional healers, herbalist and prayer centres), or presented directly to the hospital. The proportion of patients using each of these routes to diagnosis differed slightly between studies but very similar across all the eight studies. [27-30 34 35 37 38] On the average, around a third of the participants - across the studies - initially presented symptoms to either the physician, alternative medicine practitioners or directly to the hospital.

For peer review only - http://bmjopen.bmj.com/site/about/guidelines.xhtml

Cervical cancer

Two of the five studies on cervical cancer were pilot studies of low-cost screening methods (VIA and VILI) for the disease (Table 2).[44 45] In both studies, following screening, around 2% of the participants were diagnosed with cervical neoplasia (a precancerous lesion), and less than 1% had cervical cancer.[44 45] In two of the remaining three studies, all of the participants presented with symptoms directly to tertiary health centres, predominantly with late-stage cervical cancer.[33 36] Conversely, 47% of participants in the fifth study (a cross-sectional study) initially presented symptoms to traditional health practitioners before returning to the hospital for diagnosis and treatment.[42]

Colorectal cancer

In a survey of 82 patients with rectal bleeding, Alatise *et al.* found that only 39% of the participants had consulted a physician, with 38% of patients suggesting that herbs should be used before going to the doctors (Table 2).[32]

Kaposi's Sarcoma

Of 6,292 HIV infected patients enrolled at an HIV clinic, Chu *et al.* found that 3% were diagnosed with Kaposi's Sarcoma within seven years of routine HIV care.[39] Similarly, Freeman *et al.* showed that 1,328 HIV patients from 33 HIV clinics across five African countries were diagnosed with Kaposi's Sarcoma during a four-year period of routine HIV care at the clinics.[46]

Childhood cancer

Two studies surveyed parents and carers of children with childhood cancers to determine the causes of diagnostic delay. In one of the studies, 59% of parents initially sought alternative medicine for their children, although about 60% later consulted in primary care, 38% in secondary care, and 2% presented directly to tertiary care.[43] In contrast, 69% of parent in the second study initially sought conventional medical help, but 24% either self-medicated, used herbalist or presented to a church.[31]

Lymphoma

For peer review only - http://bmjopen.bmj.com/site/about/guidelines.xhtml

For peer review only

In a survey of parents and carers of children with Burkitt lymphoma, Afungchwi and colleagues showed that 55% had used traditional healers before hospital admission, with 42% using this service before reporting to primary care.[41] In contrast, all 163 patient diagnosed with Hodgkin and Non-Hodgkin lymphoma in Antel *et al's* study were referred to the specialist by healthcare practitioners.[40]

Discussion

The route to diagnosis is a strong predictor of cancer outcomes.[47 48] In this review, we examined the evidence relating to cancer diagnosis in SSA. Across all studies included in the review, diagnoses of cancer were made at the specialist clinic in large tertiary health centres, except for Kaposi's sarcoma for which cases were detected at primary care based specialist clinics. However, participants' journey to the specialist clinics is less definitive, with a considerable proportion initially using alternative medicine before consulting conventional medical services.

Strength and limitations

To our knowledge, this is the first study to systematically identify and describe the evidence regarding the routes to cancer diagnosis in SSA. Our rigorous search strategy and explicit inclusion/exclusion criteria, quality assessment of included studies and narrative synthesis followed best practice. Our search identified only a modest number of studies, a third of which were conducted in Nigeria, which is the most populous country with the largest economy in the region. For simplicity, we omitted the British and French Overseas Territories and few countries (Egypt, Morocco, Algeria, Tunisia, and Libya) which are arbitrarily classed as part of the Arab world. Health services in some of these countries are similar to those of the developed world, providing universal care through social or government contributions.[49 50] Healthcare services in many SSA countries are not universally accessible. They are pluralistic with a range of public and private providers who barely communicate with each other. The decision to omit these countries in our search strategy may have reduced the number of selected studies slightly, we have no reason to believe that such omission had any impact on our findings.

For peer review only - http://bmjopen.bmj.com/site/about/guidelines.xhtml

A limitation was that almost half of the studies focused on breast cancer, reducing the scope of the review. Importantly, any review is only as good as the studies it finds. Our final selection used small sample sizes which limits interpretation and generalisability. The majority also recruited participants and gathered data (using researchers-administered questionnaires) from the hospital facilities where patients are being treated for their cancers, typically in the tertiary health care centres. Recruiting participants from tertiary health centres systematically exclude patients treated in private hospitals and those whose cancers may never be found due to affordability or comorbidity. Furthermore, gathering data from the hospital using researchers-administered questionnaires may generate more socially desirable responses. In this case, it is likely that participants under-report their use of alternative medicine and self-medication to look good in the eyes of their providers who may be part of the research team.

We interpreted the CASP quality instrument for this review - primarily to fit our study topic: it is unlikely these minor changes led to more studies 'failing' our quality assessment. Finally, publication bias is possible as some studies on the subject may have failed to be published in reputable peer-reviewed journals, and so omitted from the databases searched for this review.

Interpretation of findings

Only two of our final selection examined screening as a form of secondary prevention or a route to diagnoses of cancer. Catarino *et al* and Paluku *et al* investigated the use of visual inspection methods for cervical cancer screening. [44 45] Recognising the inaccuracies of this screening method,[51] less than 1% of women screened in both studies had cervical cancer, with significant dropout rates among HPV-positive women. Indeed, only 69% of HPV-positive women in Catarino *et al's* study returned for further investigation or treatment. [44] The impact of screening lies in its potential to isolate early-stage curable cancers from a large population of asymptomatic patients. The sample size in both screening studies is small. One of the studies used a "screen and treat" approach, [45] which boosted participation slightly, but an extensive follow-up period and repeated testing may be necessary to determine the true

For peer review only - http://bmjopen.bmj.com/site/about/guidelines.xhtml

effectiveness of the screening. Additionally, in a region where poverty is rife, with low health literacy and poor public awareness of cancer, the findings from these studies and lessons from developed countries indicate that efforts towards early symptomatic presentation are likely to yield better cancer outcomes compared to screening in this region. [16 52-54]

However, a considerable proportion of participants in this review initially used alternative medicine before consulting in primary care, with some presenting directly to the hospital. Only a third of women with breast cancer initially reported symptoms to primary care, despite widespread awareness campaign with relatively easy to spot symptoms.[55 56] 53% of patients with cervical cancer symptoms, 39% of those with rectal bleeding and around two-third of childhood cancers initially sought help in primary care. Access to conventional health care is restricted in most SSA countries due to limited availability, affordability, and other patient and health system-related factors highlighted earlier[16-19]. In their respective cancer journey, patients in this region may start with or revert to alternative medicine, which is considered cheaper and more natural, with some practitioners offering complete cure of cancer rather than possible remission offered by conventional medicine.[20 34] The use of alternative medicine is not limited to SSA: up to 90% of childhood cancers and around 50% of adult cancers worldwide, initially use alternative medicine.[57 58] Yet, Afungchwi *et al.* showed that in patients with Burkitt lymphoma, alternative medicine practitioners common diagnosis includes liver problem, abscess, witchcraft, poison, hernia, side pain, mushroom in the belly, and toothache.[41] Alternative medicine is associated with advanced-stage diagnosis and poorer survival of major cancer types,[59] and so may, in part, explain the poorer outcomes of cancer in SSA.

The findings of this review may have been influenced by the level of biases in our final selection, in which case, our report on the proportion using various route to diagnosis is inaccurate. If at all, we may have overestimated the proportion of patients consulting in primary care or underestimated those using alternative medicine before diagnosis, given the lack of public awareness of cancer and weakness of health care systems in the region, with significant underdiagnoses.

For peer review only - http://bmjopen.bmj.com/site/about/guidelines.xhtml

Conclusion

Recent data and evidence from SSA suggest a rapid increase in the risk and deaths from major cancer types. In a region where the rates of infectious diseases persist, with limited health budget and shortage of specialists, urgent solutions are required to minimise the burden of cancer on its rapidly growing and ageing population. As yet, there are no organised national cancer screening programmes in most countries in SSA. Indeed, the facilities and expertise required to implement a successful screening programme are absent in many of the countries. Therefore, interventions aimed at promoting earlier symptomatic presentation and reduced intervals of diagnosis are likely to yield better cancer outcomes. The majority of participants in our selected studies initially presented symptoms in primary care, though the proportion using alternative medicine first is considerable. This latter group of patients constitutes a major source of concern, bearing in mind that alternative medicine practitioners are untrained or equipped to spot cancer or a make specialist referral when necessary. Government and health department in SSA may consider ways of integrating alternative medicine within the mainstream health system to ensure that patients with possible cancer are diagnosed and treated promptly when their cancer can be cured.

For peer review only

For peer review only - http://bmjopen.bmj.com/site/about/guidelines.xhtml

Table 1: Search terms

Population	Exposure	Outcome
**Terms relating Sub-Saharan Countries:**<br>Angola, Gabon, Nigeria, Benin, Gambia, The Rwanda, Botswana, Ghana, São Tomé and Principe, Burkina Faso, Guinea, Senegal, Burundi, Guinea-Bissau, Seychelles, Cabo Verde, Kenya, Sierra Leone, Cameroon, Lesotho, Somalia, Central African Republic, Liberia, South Africa, Chad<br>Madagascar, South Sudan, Comoros, Malawi, Sudan, Congo, Dem. Rep., Mali, Swaziland, Congo Rep., Mauritania, Tanzania, Côte d'Ivoire, Mauritius, Togo, Equatorial Guinea, Mozambique, Uganda, Eritrea, Namibia, Zambia, Ethiopia, Niger, Zimbabwe	**Terms relating to Cancer:**<br>Cancer, Neoplasm, Malignant Neoplasm, tumour, Malignant tumour, Astrocytoma, Adenocarcinoma, Glioma, Mesothelioma, Medulloblastoma, Myeloma, Melanoma, Neuroblastoma, Sarcoma, Nonmelanoma, Osteosarcoma, Teratoma, Seminoma, Hodgkin, Leukaemia, Lymphoma, Retinoblastoma	**Terms relating to the routes to Cancer Diagnosis:**<br>Pathway to diagnosis<br>Pathway to detect*<br>Routes to diagnos*<br>Routes to detect*<br>Diagnos*<br>Detect*<br>Consult*<br>Help-seek*<br>Present*<br>Route to consult*<br>Routes to present*<br>Pathway to consult*<br>Pathway to present*<br>Primary care<br>Family doctor<br>Physician<br>Health care practitioner<br>General Practitioners<br>Family Practice<br>Primary Health Care

For peer review only

For peer review only - http://bmjopen.bmj.com/site/about/guidelines.xhtml

Table 2: Study characteristics

Author	Country	Type/site	Title	Method	Outcome measure	Sample characteristics	Relevant findings
Dye *et al.* 2010[34]	**Ethiopia**	**Breast**	Complex care systems in developing countries: breast cancer patient navigation in Ethiopia	A mixed-methods study using semi-structure interview to investigate participant's navigation through the health system before arriving at the tertiary health centre for treatment.	Patient navigation through the health care system that culminated in the treatment of cancer	**Participants:** 55 patients with breast cancer plus 14 carers.<br>**Mean age:** 45.5 years<br>**Sex:** 98% females	Initial presentation of symptoms was:<br>53.7% to primary care<br>16.4% to traditional healers<br>16.4% to local/regional hospital<br>9% to private hospital<br>4.5% directly to the tertiary referral centre.<br>Definitive diagnoses were made at the tertiary health centre.
Jemebere 2019[35]	**Ethiopia**	**Breast**	Barriers Associated with Presentation Delay among Breast Cancer Patients at Hawassa University Comprehensive and Specialized Hospital, Southern Ethiopia	Cross-sectional survey of women diagnosed with breast cancer at a specialised hospital	Route to diagnosis	**Participants:** 106 women<br>**Age range:** 15 - 65years.<br>**Occupation:** Farmers (3%), Labourer (9%), Merchant (16%), Professional 29%) and Housewife (43%).<br>**Education:** None (28%)<br>Elementary (29%), High (24%) and College≥ (19%).<br>Family history of breast cancer (13%)	64% delayed presenting to the hospital due to initial use of alternative medicine including: herbal remedy, traditional healers and prayers.<br><br>Definitive diagnoses were made at the hospital.
Ezeome *et al.* 2010[27]	**Nigeria**	**Breast**	Delays in presentation and treatment of breast cancer in Enugu, Nigeria	Cross-sectional survey of breast cancer patients at an oncology specialist unit.	Patients first point of symptom(s) presentation<br><br>Patients first point of conventional medical treatment	**Participants:** 164 patients (162 female & 2 males) **Median (range) age:** 45 (21-77) years<br>**Socioeconomic status:**<br>Low (59%), Middle (40%), High (1%)<br>**Education:** None (15%), Primary (24%), Secondary (29%), Degree (30%)<br>**Religion:** 96% were Christians	13.1% first used traditional healers<br>4.4% first presented to a prayer house.<br>82.3% initially presented to health care facilities.<br>First point of contact with medical facilities:<br>50% within primary (GP)<br>25% to consultant surgeon<br>10.1% to patent medicine dealer<br>7.5% to a gynaecologist<br>5% to a nurse or allied health professional
Adamu *et al.* 2012[28]	**Nigeria**	**Breast**	Management and Outcomes of Male Breast Cancer in Zaria, Nigeria	A retrospective study using 10 years medical records of men with breast cancer in a specialist oncology centre	Route to diagnosis	**Participants:** 57 men<br>**Mean (SD) age:** 59 ±2.3 years	○ 21% initially consulted traditional healers before presenting to the specialist.<br>○ 49% first presented symptoms to the specialist, where all definitive diagnoses were made.
Pruitt *et al.* 2015[29]	**Nigeria**	**Breast**	Social barriers to diagnosis and treatment of breast cancer in patients presenting at a teaching hospital in Ibadan, Nigeria	A qualitative study which used semi-structured interview of patients with breast cancer in a teaching hospital.	Help-seeking behaviour after noticing symptoms.	**Participants:** 31 women with breast cancer.<br>**Median (range) age:** 51 (28-80≥)years<br>**Education:** None (n=7), Primary/Secondary (n= 15)<br>Tertiary (n=9).<br>**Religion:** Christians (83%) and Muslim (17%)	Most women rapidly sought orthodox medical care once they noticed symptoms, however few reported seeking herbal/spiritual help initially.<br><br>Definitive diagnoses were made at the teaching hospital.

For peer review only - http://bmjopen.bmj.com/site/about/guidelines.xhtml

Adesunkanmi *et al.* *2006*[30]	**Nigeria**	**Breast**	The severity, outcome and challenges of breast cancer in Nigeria	A retrospective study using 8 years records of patient with breast cancer diagnosis in a tertiary health centre.	To determine the challenges of breast cancer diagnosis at the centre.	**Participants:** 212 patients<br>**Sex:** 99.5% female<br>**Mean (SD) age:** 48±12.3 years.<br>**Occupation:** Traders (52%), Teachers (31.6%), Nurses (5.5%), Farmers (4%), and Self-employed (1.4%).<br>**Education:** Primary (18%), Secondary (14%) and Tertiary (35%).<br>Previous breast disease (25%) Family history of breast cancer (7.2%)	○ 92% of the tumour were self-detected<br>○ 4.2% by physicians<br>○ and 3.8% by partners<br>○ Definitive diagnoses were made at the tertiary health centre
Aziato *et al.* 2015[37]	**Ghana**	**Breast**	Breast Cancer Diagnosis and Factors Influencing Treatment Decisions in Ghana	A qualitative study using face-to-face interviews of breast cancer patients from a Surgical Unit and breast cancer support group.	Route to diagnosis	**Participants:** 12 female<br>**Age range:** 31-60 years<br>**Religion:** All were Christians	Women self-identified breast lesion or accidently during medical examination for other problems.<br>Participants who self-identified breast lesion presented to the tertiary health centre where definitive diagnoses were made.
Adegokey *et al. 2019*[38]	**Ghana**	**Breast**	Knowledge and Health Seeking Behaviour of Breast Cancer Patients in Ghana	A qualitative study using in-depth interviews to examine help-seeking behaviour of female breast cancer patients at a teaching hospital	Patient help-seeking behaviour after noticing female breast symptoms	**Participants:** 20 females<br>**Median (range) age:** 52.5 (29-80) years<br>**Occupation:** Traders (n=11), Teachers (n=3) Farmers (n=5) and Nurse (n=1).<br>**Education: none (n=4)** Primary (n=12), Secondary (n=1), and Tertiary (n=3).<br>**Religion:** 95% were Christians	12/20 first sought unorthodox care (herbalist, drug stores, home remedies and prayer camps) after noticing symptoms. Some patient went through a cycle of hospital-herbalist- and back to hospital care before diagnosis.<br>Definitive diagnoses were made at the tertiary health centre
Catarino *et al.* 2015[44]	**Madagascar**	**Cervical**	Smartphone Use for Cervical Cancer Screening in Low-Resource Countries: A Pilot Study Conducted in Madagascar	A pilot study of Human Papilloma Virus self-sampling test (self HPV) and Digital Imaging after - Visual inspection of the cervix as possible cervical cancer screening tools.	Cervical cancer diagnosis	**Participants:** 332 women<br>**Mean (SD) age:** 44.7±9.4yrs.<br>**Occupation:** Employed (12%), Farmers (32%), Independent (18%), Housewife (17%) and Others (22%).<br>**Education:** None (12.6%), Primary (41%), Secondary (44%) and Tertiary (2%).<br>25% had previous breast disease and 7.2% had a family history of breast cancer.	137/332 women who self-HPV were found to be HPV positive.<br>95/137 of HPV positive had D-VIA and 8 of these were diagnosed with cervical neoplasia
Paluku *et al.* 2019[45]	**Democratic Republic of Congo**	**Cervical**	Massive single visit cervical pre-cancer and cancer screening in eastern Democratic Republic of Congo	A pilot study of cervical screening with Visual inspection of the cervix with acetic acid (VIA) and Visual inspection of the cervix with Lugol iodine solution (VILI). Participants were identified after a series of announcements in churches and on the radio.	Cervical cancer diagnosis	**Participants:** 644 females participated in the screening programme<br>**Mean (SD) age:** 38.8±13.3years.	○ Following screening with VIA and VILI, 48/644 women had biopsy.<br>○ Cervical intraepithelial neoplasia (CIN) was identified in 15 (2.33%)<br>○ and squamous cell carcinoma (SCC) was identified in 6 (0.93%)
Mlange *et al. 2016*[42]	**Tanzania**	**Cervical**	Patient and disease characteristics associated with late tumour stage at	A cross-sectional survey of women with histologically confirmed cervical cancer at a tertiary health centre	Route to diagnosis	**Participants:** 202 women with cervical cancers<br>**Mean (SD) age:** 50±11 years	○ 95 (47%) initially presented to traditional health practitioner.<br>○ Presenting to a traditional health practitioner was strongly associated with

For peer review only

			presentation of cervical cancer in north-western Tanzania			**Education:** None (57%) Primary (39%), Secondary (2.4%), and College (1.4%).<br>**Occupation:** Farmers (84%), Trader (9.9%), Employed (2.4%), Business (0.9%) and Unemployed (2.4%)	late-stage diagnosis (Odds Ratio = 2.3 [95% CI 1.2–4.2], p = 0.011)
Begoihn *et al.* 2019[36]	**Ethiopia**	**Cervical**	Cervical cancer in Ethiopia – predictors of advanced stage and prolonged time to diagnosis	A retrospective cohort study of patients diagnosed with primary cervical cancer at a tertiary health centre	Route to diagnosis of cervical cancer	**Participants:** 1,575 women with cervical cancers<br>**Mean (SD) age:** 48.9±11.5 years	○ All of the 1,575 women presented cervical cancer symptoms to tertiary health centre.
Eze *et al.* 2013[33]	**Nigeria**	**Cervical**	A Six-Year Study of the Clinical Presentation of Cervical Cancer and the Management Challenges Encountered at a State Teaching Hospital in Southeast Nigeria	A retrospective cohort study of patients diagnosed with primary cervical cancer at a tertiary health centre	Route to diagnosis of cervical	**Participants:** 61 women with primary cervical cancers<br>**Mean (SD) age:** 54±12.7 years<br>**Education:** None (36.1%) Primary (39.3%), Secondary (23%), and College (1.6%).<br>**Occupation:** Farmers (60%), Trader (37.7%), Dependent (36.1%), Business (0.9%) and Retired (6.6%)	○ All of the 61 women presented cervical cancer symptoms to tertiary health centre.
Alatise *et al.* 2017[32]	**Nigeria**	**Colorectal**	Health-Seeking Behaviour and Barriers to Care in Patients With Rectal Bleeding in Nigeria	A prospective survey of patients with rectal bleeding in the general population.	Attitude about seeking expert opinion among patient with rectal bleeding.<br><br>Initial help-seeking after the onset of rectal bleeding	**Participants:** 82 patients with rectal bleeding<br>**Median (range) age:** 45 (18-85) years<br>**Sex:** 78% were males<br>**Education:** Primary (28%), Secondary (33%), tertiary (30%)<br>**Religion:** Christians (66%) and Muslims (33%)	○ 39% of the participants consulted a physician for rectal bleeding.<br>○ 38% suggested that herbs should be used before seeing a physician.<br>○ Patients who scored high on knowledge of rectal bleeding were more likely to consult the physician (Odds ratio: 3.82; 95% CI, 55-10.2).
Chu *et al.* 2010[39]	**South Africa**	**Kaposi's Sarcoma**	AIDS-associated Kaposi's sarcoma is linked to advanced disease and high mortality in a primary care HIV programme in South Africa	Analysis of data from a cohort study of patients with AIDS-associated Kaposi's sarcoma in primary care	Patient pathway to diagnosis of Kaposi's Sarcoma	**Participants:** 215 patients with Kaposi's Sarcoma<br>**Median (interquartile range) age:** 34 (29 -41) years<br>**Sex:** 41% were females	189/6292 patients enrolled at the HIV Clinic were diagnosed with AIDS-associated Kaposi's sarcoma
Freeman *et al.* 2016[46]	**Kenya Uganda Malawi Nigeria Cameroon**	**Kaposi's Sarcoma**	Pitfalls of practicing cancer epidemiology in resource-limited settings: the case of survival and loss to follow-up after a diagnosis of Kaposi's sarcoma in five countries across sub-Saharan Africa	Analysis of HIV-infected patients' in primary care records across five countries.	Route to diagnosis of Kaposi's Sarcoma	**Participants:** 1,328 patients with Kaposi's Sarcoma **Median (interquartile range) age:** 35 (30 -41) years<br>**Sex:** 40% were females	During routine HIV care, 1,328 patients were diagnosed with Kaposi's sarcoma across the five countries between 2009 and 2012
Afungchwi *et al.* 2017[41]	**Cameroon**	**Burkitt Lymphoma**	The role of traditional healers in the diagnosis and management of Burkitt lymphoma in Cameroon: understanding the challenges and moving forward	A survey of parents and carers of children diagnosed with Burtkitt lymphoma in three large hospitals	Route to diagnosis Burkitt Lymphoma	**Participants:** 384 questionnaire completed<br>**Median (range) age:** 8 (1 -15) years.<br>**Sex:** Males (57.4%) and females (42.4%)<br>**Religion:** Christians (68.9%) and Muslims (30%)	○ Overall, 55% of parents used traditional healers before hospital admission<br>○ 41.8% first consulted traditional healers before reporting at local health centre.

For peer review only - http://bmjopen.bmj.com/site/about/guidelines.xhtml

Antel *et al.* 2019	**South Africa**	**Lymphoma**	The determinants and impact of diagnostic delay in lymphoma in a TB and HIV endemic setting	A retrospective cohort study of patients diagnosed with lymphomas. Data sources included hospital records, telephone and face-to-face interviews.	Route to diagnosis of Hodgkin and Non-Hodgkin lymphoma	**Participants:** 163 HIV patients<br>**Median (range) age:** 48 (15-86) years<br>**Sex:** 58% were males<br>**Socioeconomic status:** 70% on social grant or <251 monthly income	All 163 HIV patients were diagnosed with Hodgkin (41) and Non-Hodgkin (122) lymphoma. They were referred to the tertiary health centre by *healthcare practitioners*
Brown *et al.* 2015[31]	**Nigeria**	**Childhood**	A Prospective Study on the Causes of Delayed Diagnosis of Childhood Cancer in Ibadan, Nigeria	A survey of parents and carers of children diagnosed with malignant tumour in a tertiary health care settings	Factors influencing pre-diagnostic intervals among parent/carers of patient with childhood cancers	**Participants:** 91 children with cancer<br>**Median (range) age:** 4years (1month -15years).<br>**Sex:** 50.5% were males	○ 69% of parent initially sought medical help for their children within a health facility<br>○ 19% self-medicated<br>○ 4% used herbalist,<br>○ 3% consulted a patent medicine dealers<br>○ 2% presented to a nurse/health worker and 1% visited a church.<br><br>Health facilities users comprised: 69% Public hospital, 31% Private
Njuguna *et al.* 2016[43]	**Kenya**	**Childhood**	Factors influencing time to diagnosis and treatment among paediatric oncology patients in Kenya	A cross-sectional survey of parent and carers of 99 children diagnosed with a malignancy.	Help-seeking after the onset of symptoms.	**Participants:** 99 children with cancer<br>**Median age:**<br>Children: 5.7years<br>Mother: 31 (19-56) years.<br>**Sex:** 67% were male.<br>**Religion:** 99% of mothers were Christians.<br>**Employment:** Farmers (29%), R*egular jobs (24%),* Casual labourers (6%) and Unemployed (6%)	58 (59%) of parent initially sought alternative treatment for their children, including: praying ceremonies (41%), visiting herbalist (36%), special food intake (11%), and attending traditional healer (3%).<br>First contact with conventional health care facilities included: 60% in primary care, 38% in secondary, and 2% in tertiary health care.

Note: SD Standard Deviation; CI Confidence Interval

For peer review only - http://bmjopen.bmj.com/site/about/guidelines.xhtml

Table 3: Quality of studies

CASP questions for cohort studies	***Was the cohort representative of a defined population***	***Was the exposure accurately measured to minimise bias?***	***Was the outcome accurately measured to minimise bias?***	***Have the authors identified and adjusted for all key confounding factors?***	***How precise are the results?***	***Can the results be applied to the local population?***	***Overall quality***
Adapted question	*Unchanged*	*Was cancer diagnosis appropriately ascertained*	*Was initial consultation(s) accurately defined*	*Unchanged*	*Have they presented estimates of association along with the confidence intervals? Are the confidence intervals narrow?*	*Unchanged*	
Dye *et al.* 2010[34]	Partially met	Met	Partially met	Unmet	Partially met	Partially met	sat
Jemebere 2019	Partially met	Met	Partially met	Unmet	Unmet	Partially met	Sat
Ezeome *et al.* 2010[27]	Partially met	Met	Met	Unmet	Unmet	Partially met	Sat
Adamu *et al.* 2012[28]	Partially met	Met	Partially met	Unmet	Unmet	Partially met	Sat
Adesunkanmi *et al. 2006*	Unmet	Met	Unmet	Unmet	Unmet	Partially met	Low
Catarino *et al.* 2015	Partially met	Met	Met	Partially met	Partially met	Partially met	Sat
Paluku *et al.* 2019	Partially met	Met	Met	Partially met	Partially met	Partially met	Sat
Mlange *et al. 2016*	Partially met	Met	Partially met	Unmet	Unmet	Partially met	Sat
**Begoihn *et al.*** 2019	Partially met	Met	Partially met	N/A	Unmet	Partially met	Sat
**Eze *et al.*** 2013	Partially met	Met	Partially met	Unmet	Unmet	Partially met	Sat
Alatise et al. 2017	Partially met	Met	Partially met	Unmet	Partially met	Partially met	Sat
Chu *et al. 2010*	Partially met	Met	Unmet	N/A	Unmet	Partially met	Sat
Freeman *et al.* 2016[46]	Partially met	Partially met	Unmet	Partially met	Unmet	Partially met	Sat
Afungchwi *et al.* 2017[41]	Partially met	Met	Partially met	Unmet	Unmet	Partially met	Sat
Antel *et al.* 2019	Partially met	Met	Partially met	Partially met	Unmet	Unmet	Sat
Brown *et al.* 2015[31]	Partially met	Met	Partially met	Unmet	Unmet	Partially met	Sat
Njuguna *et al.* 2016[43]	Partially met	Met	Partially met	Partially met	Unmet	Partially met	Sat
CASP questions for qualitative studies	***Was the research design appropriate to address the aims of the research?***	***Was the recruitments strategy appropriate to the aims of the research?***	***Was the data collected in a way that addressed the research issue?***	***Has the relationship between researcher and participants been adequately considered?***	***Was the data analysis sufficiently rigorous?***	***How valuable is the research?***	***Overall quality***
Pruitt *et al. 2015*	Partially met	Partially met	Partially met	Unmet	Partially met	Partially met	Sat
Aziato *et al.* 2015	Partially met	Partially met	Partially met	Unmet	Met	Partially met	Sat
Adegokey *et al. 2019*	Partially met	Met	Partially met	Partially met	Met	Partially met	Sat

Note: Sat satisfactory quality paper; N/A not applicable

For peer review only - http://bmjopen.bmj.com/site/about/guidelines.xhtml

Competing interests: The authors declare that they have no competing interests.

Authors' contributions: TM was involved in all aspects. WH participated in the study design, data interpretation and preparation and revision of the manuscript. All authors read and approved the final manuscript.

Ethical approval: Not applicable.

Acknowledgements: Not applicable

Funding: Authors disclose no funding sources

For peer review only

References

1. Gouda HN, Charlson F, Sorsdahl K, et al. Burden of non-communicable diseases in sub-Saharan Africa, 1990–2017: results from the Global Burden of Disease Study 2017. *The Lancet Global Health* 2019;7(10):e1375-e87. doi: 10.1016/S2214-109X(19)30374-2
2. Global Cancer Observatory. Estimated number of incident cases from 2018 to 2040, all cancers, both sexes, all ages 2018. Available from: http://gco.iarc.fr/tomorrow/graphic-line?type=0&population=900&mode=population&sex=0&cancer=39&age_group=value&apc_male=0&apc_female=0. [cited 2019 03/10].
3. Observatory GC. Estimated number of deaths from 2018 to 2040, all cancers, both sexes, all ages 2018. Available from: http://gco.iarc.fr/tomorrow/graphic-isotype?type=1&population=900&mode=population&sex=0&cancer=39&age_group=value&apc_male=0&apc_female=0. [cited 2019 03/10]
4. Busolo DS, Woodgate RL. Cancer prevention in Africa: a review of the literature. *Glob Health Promot* 2015;22(2):31-9. doi: 10.1177/1757975914537094 [published Online First: 2014/07/17]
5. Ferlay J, Soerjomataram I, Dikshit R, et al. Cancer incidence and mortality worldwide: Sources, methods and major patterns in GLOBOCAN 2012. *Int J Cancer* 2015;136(5):E359-E86. doi: 10.1002/ijc.29210
6. Plummer M, de Martel C, Vignat J, et al. Global burden of cancers attributable to infections in 2012: a synthetic analysis. *The Lancet Global Health* 2016;4(9):e609-e16. doi: 10.1016/S2214-109X(16)30143-7
7. The World Bank. Disease and Mortality in Sub-Saharan Africa. Washington (DC): The International Bank for Reconstruction and Development/The World Bank. 2006.
8. Azubuike SO, Muirhead C, Hayes L, et al. Rising global burden of breast cancer: the case of sub-Saharan Africa (with emphasis on Nigeria) and implications for regional development: a review. *World J Surg Oncol* 2018;16(1):63. doi: 10.1186/s12957-018-1345-2
9. Musgrove DTJJGBARMGAMCDBEPJAMP. Disease Control Priorities in Developing Countries (Second Edition).
10. Brawley OW. The role of government and regulation in cancer prevention. *The Lancet Oncology* 2017;18(8):e483-e93. doi: https://doi.org/10.1016/S1470-2045(17)30374-1
11. McPhail S, Johnson S, Greenberg D, et al. Stage at diagnosis and early mortality from cancer in England. *Br J Cancer* 2015;112 Suppl 1(Suppl 1):S108-S15. doi: 10.1038/bjc.2015.49
12. Jedy-Agba E, McCormack V, Adebamowo C, et al. Stage at diagnosis of breast cancer in sub-Saharan Africa: a systematic review and meta-analysis. *The Lancet Global Health* 2016;4(12):e923-e35. doi: https://doi.org/10.1016/S2214-109X(16)30259-5
13. Stefan DC. Cancer Care in Africa: An Overview of Resources. *J Glob Oncol* 2015;1(1):30-36. doi: 10.1200/JGO.2015.000406
14. Cassim S, Chepulis L, Keenan R, et al. Patient and carer perceived barriers to early presentation and diagnosis of lung cancer: a systematic review. *BMC Cancer* 2019;19(1):25. doi: 10.1186/s12885-018-5169-9
15. Vrinten C, Wardle J, Marlow LAV. Cancer fear and fatalism among ethnic minority women in the United Kingdom. *Br J Cancer* 2016;114(5):597-604. doi: 10.1038/bjc.2016.15
16. Kingham TP, Alatise OI, Vanderpuye V, et al. Treatment of cancer in sub-Saharan Africa. *Lancet Oncol* 2013;14(4):e158-67. doi: 10.1016/s1470-2045(12)70472-2 [published Online First: 2013/04/09]
17. Olaleye O, Ekrikpo U. Epidemiology of Cancers in Sub-Saharan Africa. In: Adedeji OA, ed. Cancer in Sub-Saharan Africa: Current Practice and Future. Cham: Springer International Publishing 2017:3-19.
18. Economist Intelligence Unit. The future of healthcare in Africa. 2012 [Available from: https://perspectives.eiu.com/sites/default/files/EIU-Janssen_HealthcareAfrica_Report_Web.pdf accessed 27/11 2019.
19. Campbell J DG, Buchan J, Pozo-Martin F, Guerra Arias M, Leone C, Siyam A, Cometto G.,. The future of healthcare in Africa,. Forum Report, Third Global Forum on Human Resources for Health, Recife, Brazil Geneva, Global Health Workforce Alliance and World Health Organization, 2013.
20. James PB, Wardle J, Steel A, et al. Traditional, complementary and alternative medicine use in Sub-Saharan Africa: a systematic review. *BMJ Global Health* 2018;3(5):e000895. doi: 10.1136/bmjgh-2018-000895
21. Mash R, Howe A, Olayemi O, et al. Reflections on family medicine and primary healthcare in sub-Saharan Africa. *BMJ Global Health* 2018;3(Suppl 3):e000662. doi: 10.1136/bmjgh-2017-000662
22. Moher D, Shamseer L, Clarke M, et al. Preferred reporting items for systematic review and meta-analysis protocols (PRISMA-P) 2015 statement. *Systematic Reviews* 2015;4(1):1. doi: 10.1186/2046-4053-4-1

For peer review only - http://bmjopen.bmj.com/site/about/guidelines.xhtml

23. The World Bank. Data Catalog: Regions and Countries 2019 [Available from: https://datacatalog.worldbank.org/ accessed 10/10 2019.
24. Rodgers M, Sowden A, Petticrew M, et al. Testing methodological guidance on the conduct of narrative synthesis in systematic reviews: effectiveness of interventions to promote smoke alarm ownership and function. *Evaluation* 2009;15(1):49-73.
25. Critical Appraisal Skills Programme. CASP Appraisal Checklists 2018 [Available from: https://casp-uk.net/casp-tools-checklists/ accessed 06/09 2019.
26. Singh S. Endovista 2016 report. *Journal of Pharmacology and Pharmacotherapeutics* 2013;4(1):76-77. doi: 10.4103/0970-7212.184348
27. Ezeome ER. Delays in presentation and treatment of breast cancer in Enugu, Nigeria. *Niger J Clin Pract* 2010;13(3):311-6.
28. Ahmed A, Ukwenya Y, Abdullahi A, et al. Management and Outcomes of Male Breast Cancer in Zaria, Nigeria. *Int J Breast Cancer* 2012;2012:6. doi: 10.1155/2012/845143
29. Pruitt L, Mumuni T, Raikhel E, et al. Social barriers to diagnosis and treatment of breast cancer in patients presenting at a teaching hospital in Ibadan, Nigeria. *Glob Public Health* 2015;10(3):331-44. doi: https://dx.doi.org/10.1080/17441692.2014.974649
30. Adesunkanmi ARK, Lawal OO, Adelusola KA, et al. The severity, outcome and challenges of breast cancer in Nigeria. *The Breast* 2006;15(3):399-409. doi: https://doi.org/10.1016/j.breast.2005.06.008
31. Brown BJ, Adeleye AO, Ibeh JN. A Prospective Study on the Causes of Delayed Diagnosis of Childhood Cancer in Ibadan, Nigeria. *Pediatr Hematol Oncol* 2015;32(6):365-73. doi: https://dx.doi.org/10.3109/08880018.2015.1040933
32. Alatise OI, Fischer SE, Ayandipo OO, et al. Health-Seeking Behavior and Barriers to Care in Patients With Rectal Bleeding in Nigeria. *J Glob Oncol* 2017;3(6):749-56. doi: https://dx.doi.org/10.1200/JGO.2016.006601
33. Eze JN, Emeka-Irem EN, Edegbe FO. A Six-Year Study of the Clinical Presentation of Cervical Cancer and the Management Challenges Encountered at a State Teaching Hospital in Southeast Nigeria. *Clinical Medicine Insights: Oncology* 2013(7):151-58. doi: 10.4137/CMO.S12017
34. Dye TD, Bogale S, Hobden C, et al. Complex care systems in developing countries: breast cancer patient navigation in Ethiopia. *Cancer* 2010;116(3):577-85. doi: https://dx.doi.org/10.1002/cncr.24776
35. Jemebere W. Barriers Associated with Presentation Delay among Breast Cancer Patients at Hawassa University Comprehensive and Specialized Hospital, Southern Ethiopia. *International Journal of Caring Sciences* 2019;12(3):1356-63.
36. Begoihn M, Mathewos A, Aynalem A, et al. Cervical cancer in Ethiopia – predictors of advanced stage and prolonged time to diagnosis. *Infectious Agents and Cancer* 2019;14(1):36. doi: 10.1186/s13027-019-0255-4
37. Aziato L, Clegg-Lamptey JNA. Breast Cancer Diagnosis and Factors Influencing Treatment Decisions in Ghana. *Health Care Women Int* 2015;36(5):543-57. doi: 10.1080/07399332.2014.911299
38. Agbokey F, Kudzawu E, Dzodzomenyo M, et al. Knowledge and Health Seeking Behaviour of Breast Cancer Patients in Ghana. *Int J Breast Cancer* 2019;2019 (no pagination)
39. Chu KM, Mahlangeni G, Swannet S, et al. AIDS-associated Kaposi's sarcoma is linked to advanced disease and high mortality in a primary care HIV programme in South Africa. *J Int AIDS Soc* 2010;13:23. doi: https://dx.doi.org/10.1186/1758-2652-13-23
40. Antel K, Levetan C, Mohamed Z, et al. The determinants and impact of diagnostic delay in lymphoma in a TB and HIV endemic setting. *BMC Cancer* 2019;19(1):384. doi: 10.1186/s12885-019-5586-4 [published Online First: 2019/04/27]
41. Afungchwi GM, Hesseling PB, Ladas EJ. The role of traditional healers in the diagnosis and management of Burkitt lymphoma in Cameroon: understanding the challenges and moving forward. *BMC Complementary & Alternative Medicine* 2017;17:1-7. doi: 10.1186/s12906-017-1719-y
42. Mlange R, Matovelo D, Rambau P, et al. Patient and disease characteristics associated with late tumour stage at presentation of cervical cancer in northwestern Tanzania. *BMC Womens Health* 2016;16(5):1-6. doi: 10.1186/s12905-016-0285-7
43. Njuguna F, Martijn H, Langat S, et al. Factors influencing time to diagnosis and treatment among pediatric oncology patients in Kenya. *Pediatr Hematol Oncol* 2016;33(3):186-99. doi: https://dx.doi.org/10.3109/08880018.2016.1169566
44. Catarino R, Vassilakos P, Scaringella S, et al. Smartphone Use for Cervical Cancer Screening in Low-Resource Countries: A Pilot Study Conducted in Madagascar. *PLoS ONE* 2015;10(7):e0134309. doi: https://dx.doi.org/10.1371/journal.pone.0134309

For peer review only - http://bmjopen.bmj.com/site/about/guidelines.xhtml

45. Paluku JL, Carter TE, Lee M, et al. Massive single visit cervical pre-cancer and cancer screening in eastern Democratic Republic of Congo. *BMC Womens Health* 2019;19(1):N.PAG-N.PAG. doi: 10.1186/s12905-019-0737-y
46. Freeman E, Semeere A, Wenger M, et al. Pitfalls of practicing cancer epidemiology in resource-limited settings: the case of survival and loss to follow-up after a diagnosis of Kaposi's sarcoma in five countries across sub-Saharan Africa. *BMC Cancer* 2016;16:65. doi: https://dx.doi.org/10.1186/s12885-016-2080-0
47. Elliss-Brookes L, McPhail S, Ives A, et al. Routes to diagnosis for cancer - determining the patient journey using multiple routine data sets. *Br J Cancer* 2012;107(8):1220-26. doi: 10.1038/bjc.2012.408 [published Online First: 2012/09/20]
48. Palser TR, Cromwell DA, Hardwick RH, et al. Impact of route to diagnosis on treatment intent and 1-year survival in patients diagnosed with oesophagogastric cancer in England: a prospective cohort study. *BMJ Open* 2013;3(2):e002129. doi: 10.1136/bmjopen-2012-002129
49. Lamri L, Gripiotis E, Ferrario A. Diabetes in Algeria and challenges for health policy: a literature review of prevalence, cost, management and outcomes of diabetes and its complications. *Globalization and Health* 2014;10(1):11. doi: 10.1186/1744-8603-10-11
50. Mahfoud N, Brahamia B. The Problems of Funding The Health System In Algeria *International Journal of Medicine and Pharmaceutical Sciences (IJMPS)* 2014;2(2):121-30

51. Visual inspection with acetic acid for cervical-cancer screening: test qualities in a primary-care setting. University of Zimbabwe/JHPIEGO Cervical Cancer Project. *Lancet* 1999;353(9156):869-73. [published Online First: 1999/03/27]
52. Moodley J, Walter FM, Scott SE, et al. Towards timely diagnosis of symptomatic breast and cervical cancer in South Africa. *S Afr Med J* 2018;108(10):803-04. doi: 10.7196/SAMJ.2018.v108i10.13478 [published Online First: 2018/11/14]
53. Hamilton W. Five misconceptions in cancer diagnosis. *Br J Gen Pract* 2009;59(563):441-47.
54. Jennifer Dent CKM, Dan Milner, Miriam Mutebi, Anne Ng'ang'a, Olufunmilayo I. Olopade, Timothy R. Rebbeck, D. Cristina Stefan. Africa's Emerging Cancer Crisis: A Call to Action, 2017.
55. A OA, Taiwo O, S AO, et al. Breast cancer awareness, attitude and screening practices in Nigeria: A systematic review. *Clinical Reviews and Opinions* 2016;7(2):11-25. doi: 10.5897/cro16.0101
56. McKenzie F, Zietsman A, Galukande M, et al. Breast cancer awareness in the sub-Saharan African ABC-DO cohort: African Breast Cancer—Disparities in Outcomes study. *Cancer Causes Control* 2018;29(8):721-30. doi: 10.1007/s10552-018-1047-7
57. Keene MR, Heslop IM, Sabesan SS, et al. Complementary and alternative medicine use in cancer: A systematic review. *Complementary Therapies in Clinical Practice* 2019;35:33-47. doi: https://doi.org/10.1016/j.ctcp.2019.01.004
58. Bishop FL, Prescott P, Chan YK, et al. Prevalence of complementary medicine use in pediatric cancer: a systematic review. *Pediatrics* 2010;125(4):768-76. doi: 10.1542/peds.2009-1775 [published Online First: 2010/03/24]
59. Johnson SB, Park HS, Gross CP, et al. Use of Alternative Medicine for Cancer and Its Impact on Survival. *JNCI: Journal of the National Cancer Institute* 2017;110(1):121-24. doi: 10.1093/jnci/djx145

For peer review only

For peer review only - http://bmjopen.bmj.com/site/about/guidelines.xhtml

Figure 1: Flow chart of study selection process

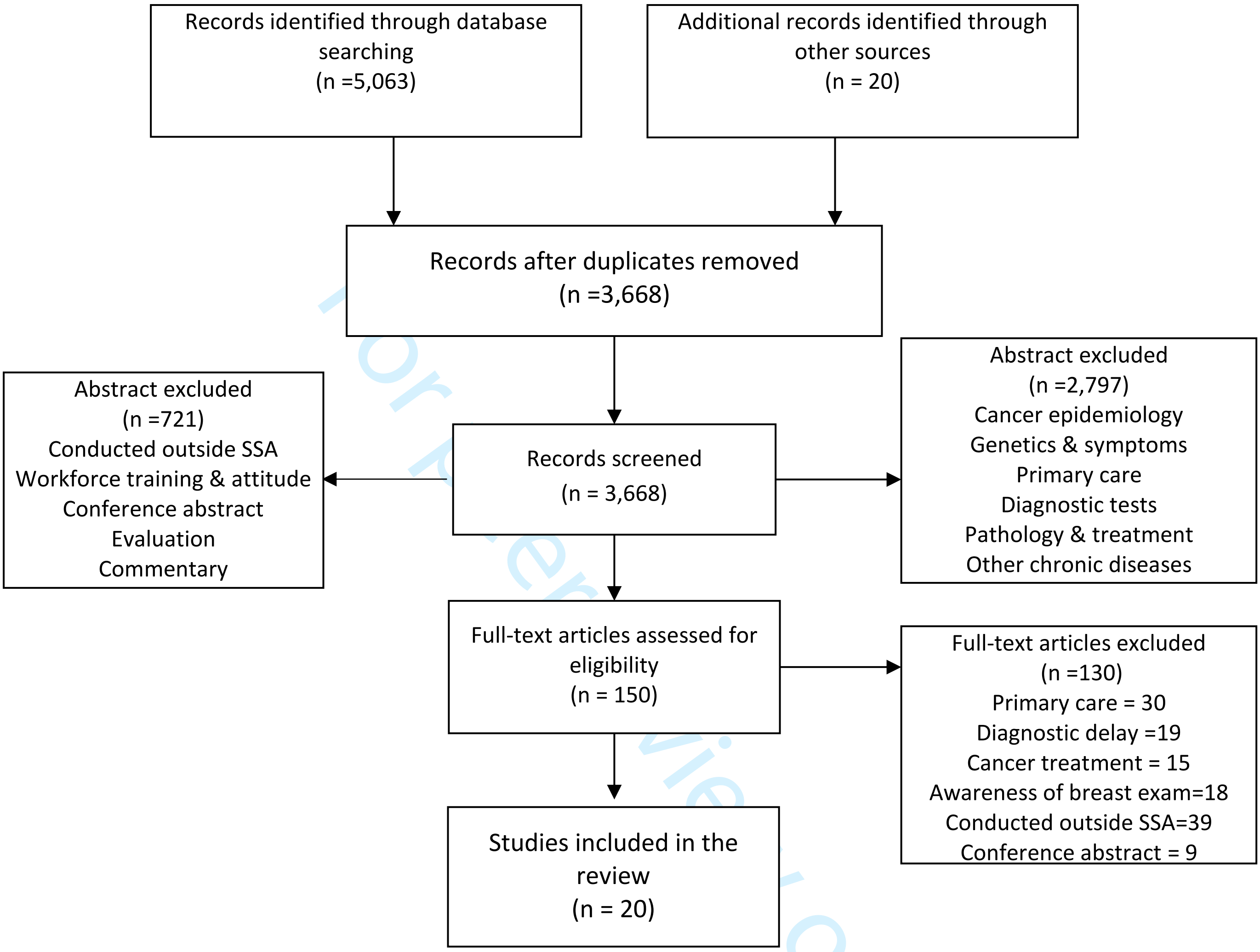

For peer review only

PRISMA 2009 Checklist

Section/topic	#	Checklist item	Reported on page #
**TITLE**			
Title	1	Identify the report as a systematic review, meta-analysis, or both.	1
**ABSTRACT**			
Structured summary	2	Provide a structured summary including, as applicable: background; objectives; data sources; study eligibility criteria, participants, and interventions; study appraisal and synthesis methods; results; limitations; conclusions and implications of key findings; systematic review registration number.	2
**INTRODUCTION**			
Rationale	3	Describe the rationale for the review in the context of what is already known.	3
Objectives	4	Provide an explicit statement of questions being addressed with reference to participants, interventions, comparisons, outcomes, and study design (PICOS).	5
**METHODS**			
Protocol and registration	5	Indicate if a review protocol exists, if and where it can be accessed (e.g., Web address), and, if available, provide registration information including registration number.	N/A
Eligibility criteria	6	Specify study characteristics (e.g., PICOS, length of follow-up) and report characteristics (e.g., years considered, language, publication status) used as criteria for eligibility, giving rationale.	5
Information sources	7	Describe all information sources (e.g., databases with dates of coverage, contact with study authors to identify additional studies) in the search and date last searched.	4-5
Search	8	Present full electronic search strategy for at least one database, including any limits used, such that it could be repeated.	Additional file 2
Study selection	9	State the process for selecting studies (i.e., screening, eligibility, included in systematic review, and, if applicable, included in the meta-analysis).	5-6
Data collection process	10	Describe method of data extraction from reports (e.g., piloted forms, independently, in duplicate) and any processes for obtaining and confirming data from investigators.	6
Data items	11	List and define all variables for which data were sought (e.g., PICOS, funding sources) and any assumptions and simplifications made.	6
Risk of bias in individual studies	12	Describe methods used for assessing risk of bias of individual studies (including specification of whether this was done at the study or outcome level), and how this information is to be used in any data synthesis.	6
Summary measures	13	State the principal summary measures (e.g., risk ratio, difference in means).	N/A
Synthesis of results	14	Describe the methods of handling data and combining results of studies, if done, including measures of consistency (e.g., I^2) for each meta-analysis.	6

For peer review only - http://bmjopen.bmj.com/site/about/guidelines.xhtml

PRISMA 2009 Checklist

Section/topic	#	Checklist item	Reported on page #
Risk of bias across studies	15	Specify any assessment of risk of bias that may affect the cumulative evidence (e.g., publication bias, selective reporting within studies).	7-8
Additional analyses	16	Describe methods of additional analyses (e.g., sensitivity or subgroup analyses, meta-regression), if done, indicating which were pre-specified.	N/A
**RESULTS**			
Study selection	17	Give numbers of studies screened, assessed for eligibility, and included in the review, with reasons for exclusions at each stage, ideally with a flow diagram.	7&20
Study characteristics	18	For each study, present characteristics for which data were extracted (e.g., study size, PICOS, follow-up period) and provide the citations.	7&15-19
Risk of bias within studies	19	Present data on risk of bias of each study and, if available, any outcome level assessment (see item 12).	7, 18, & 19
Results of individual studies	20	For all outcomes considered (benefits or harms), present, for each study: (a) simple summary data for each intervention group (b) effect estimates and confidence intervals, ideally with a forest plot.	7-8 & 15-18
Synthesis of results	21	Present results of each meta-analysis done, including confidence intervals and measures of consistency.	N/A
Risk of bias across studies	22	Present results of any assessment of risk of bias across studies (see Item 15).	7, 18, & 19
Additional analysis	23	Give results of additional analyses, if done (e.g., sensitivity or subgroup analyses, meta-regression [see Item 16]).	N/A
**DISCUSSION**			
Summary of evidence	24	Summarize the main findings including the strength of evidence for each main outcome; consider their relevance to key groups (e.g., healthcare providers, users, and policy makers).	10
Limitations	25	Discuss limitations at study and outcome level (e.g., risk of bias), and at review-level (e.g., incomplete retrieval of identified research, reporting bias).	10
Conclusions	26	Provide a general interpretation of the results in the context of other evidence, and implications for future research.	12
**FUNDING**			
Funding	27	Describe sources of funding for the systematic review and other support (e.g., supply of data); role of funders for the systematic review.	21

From: Moher D, Liberati A, Tetzlaff J, Altman DG, The PRISMA Group (2009). Preferred Reporting Items for Systematic Reviews and Meta-Analyses: The PRISMA Statement. PLoS Med 6(7): e1000097. doi:10.1371/journal.pmed1000097

For more information, visit: **www.prisma-statement.org**.

For peer review only - http://bmjopen.bmj.com/site/about/guidelines.xhtml

Additional file 2: Ovid MEDLINE(R), PsycINFO, Embase, Global Health

Set	Search Statement
1.	Angola*.ti,ab.
2.	Gabon*.ti,ab.
3.	Nigeria*.ti,ab.
4.	Benin*.ti,ab.
5.	Gambia*.ti,ab.
6.	Rwanda*.ti,ab.
7.	Botswana*.ti,ab.
8.	Ghana*.ti,ab.
9.	Sao Tome*.ti,ab.
10.	Sao Tome.mp. and Principe*.ti,ab. [mp=title, abstract, heading word, drug trade name, original title, device manufacturer, drug manufacturer, device trade name, keyword, floating subheading word, candidate term word]
11.	Burkina Faso*.ti,ab.
12.	Guinea-Bissau*.ti,ab.
13.	Seychelles*.ti,ab.
14.	Guinea*.ti,ab.
15.	Senegal*.ti,ab.
16.	Burundi*.ti,ab.
17.	Cabo Verde*.ti,ab.
18.	Kenya*.ti,ab.
19.	Sierra Leone*.ti,ab.
20.	Cameroon*.ti,ab.
21.	Lesotho*.ti,ab.
22.	Somalia*.ti,ab.
23.	Central African Republic*.ti,ab.
24.	Liberia*.ti,ab.
25.	South Africa*.ti,ab.
26.	Chad*.ti,ab.
27.	Madagascar*.ti,ab.
28.	South Sudan*.ti,ab.
29.	Comoros*.ti,ab.
30.	Malawi*.ti,ab.
31.	Sudan*.ti,ab.
32.	Congo*.ti,ab.
33.	Mali*.ti,ab.
34.	Swaziland*.ti,ab.
35.	Mauritania*.ti,ab.
36.	Tanzania*.ti,ab.
37.	Cote d'Ivoire*.ti,ab.
38.	Mauritius*.ti,ab.
39.	togo*.ti,ab.
40.	Equatorial Guinea*.ti,ab.
41.	Mozambique*.ti,ab.
42.	Uganda*.ti,ab.
43.	Eritrea*.ti,ab.
44.	Namibia*.ti,ab.
45.	Zambia*.ti,ab.
46.	Ethiopia*.ti,ab.
47.	Niger*.ti,ab.
48.	Zimbabwe*.ti,ab.
49.	Africa*.ti,ab.

For peer review only

50.	Sub-Sahara*.ti,ab.
51.	1 or 2 or 3 or 4 or 5 or 6 or 7 or 8 or 9 or 10 or 11 or 12 or 13 or 14 or 15 or 16 or 17 or 18 or 19 or 20 or 21 or 22 or 23 or 24 or 25 or 26 or 27 or 28 or 29 or 30 or 31 or 32 or 33 or 34 or 35 or 36 or 37 or 38 or 39 or 40 or 41 or 42 or 43 or 44 or 45 or 46 or 47 or 48 or 49 or 50
52.	cancer*.ti,ab.
53.	exp Neoplasms/
54.	(adenocarcinoma or astrocytoma or glioma or Hodgkin$ or leuk?emia or lymphoma or medulloblastoma or melanoma or mesothelioma or myeloma or neuroblastoma or nonmelanoma or osteosarcoma or retinoblastoma or sarcoma or seminoma or teratoma).ti,ab.
55.	52 or 53 or 54
56.	(pathway adj5 diagnos$).ti,ab.
57.	(pathway adj5 detect$).ti,ab.
58.	(route adj5 diagnos$).ti,ab.
59.	(route adj5 detect$).ti,ab.
60.	diagnos$.ti,ab.
61.	detect$.ti,ab.
62.	consult$.ti,ab.
63.	(help adj5 seek$).ti,ab.
64.	present$.ti,ab.
65.	(route adj5 consult$).ti,ab.
66.	(route adj5 present$).ti,ab.
67.	(pathway adj5 consult$).ti,ab.
68.	(pathway adj5 present$).ti,ab.
69.	56 or 57 or 58 or 59 or 60 or 61 or 62 or 63 or 64 or 65 or 66 or 67 or 68
70.	primary care.ti,ab.
71.	family doctor.ti,ab.
72.	physician.ti,ab.
73.	(health adj5 practitioner).ti,ab.
74.	General Practitioners/ or Family Practice/ or Primary Health Care/
75.	70 or 71 or 72 or 73 or 74
76.	51 and 55 and 69 and 75

For peer review only

BMJ Open

Routes to diagnosis of symptomatic cancer in Sub-Saharan Africa: a systematic review

Journal:	*BMJ Open*
Manuscript ID	bmjopen-2020-038605.R1
Article Type:	Original research
Date Submitted by the Author:	13-Aug-2020
Complete List of Authors:	Martins, Tanimola; University of Exeter, College of Medicine & Health<br>Merriel, Samuel; University of Exeter, College of Medicine & Health<br>Hamilton, Willie; University of Exeter, Primary Care
**Primary Subject Heading**:	General practice / Family practice
Secondary Subject Heading:	Epidemiology, Public health, Oncology
Keywords:	PRIMARY CARE, PUBLIC HEALTH, Epidemiology < ONCOLOGY, International health services < HEALTH SERVICES ADMINISTRATION & MANAGEMENT

SCHOLARONE™
Manuscripts

I, the Submitting Author has the right to grant and does grant on behalf of all authors of the Work (as defined in the below author licence), an exclusive licence and/or a non-exclusive licence for contributions from authors who are: i) UK Crown employees; ii) where BMJ has agreed a CC-BY licence shall apply, and/or iii) in accordance with the terms applicable for US Federal Government officers or employees acting as part of their official duties; on a worldwide, perpetual, irrevocable, royalty-free basis to BMJ Publishing Group Ltd ("BMJ") its licensees and where the relevant Journal is co-owned by BMJ to the co-owners of the Journal, to publish the Work in this journal and any other BMJ products and to exploit all rights, as set out in our licence.

The Submitting Author accepts and understands that any supply made under these terms is made by BMJ to the Submitting Author unless you are acting as an employee on behalf of your employer or a postgraduate student of an affiliated institution which is paying any applicable article publishing charge ("APC") for Open Access articles. Where the Submitting Author wishes to make the Work available on an Open Access basis (and intends to pay the relevant APC), the terms of reuse of such Open Access shall be governed by a Creative Commons licence – details of these licences and which Creative Commons licence will apply to this Work are set out in our licence referred to above.

Other than as permitted in any relevant BMJ Author's Self Archiving Policies, I confirm this Work has not been accepted for publication elsewhere, is not being considered for publication elsewhere and does not duplicate material already published. I confirm all authors consent to publication of this Work and authorise the granting of this licence.

For peer review only

Routes to diagnosis of symptomatic cancer in Sub-Saharan Africa: a systematic review

Tanimola Martins *, Postdoctoral Research Fellow [1]

Samuel W D Merriel, Clinical Senior Research Fellow [1]

William Hamilton, Professor of Primary care Diagnostics [1]

[1] University of Exeter, College of Medicine & Health, College House St Luke's Campus, Magdalen Road, Exeter, EX1 2LU

*Correspondence author's email: tom207@exeter.ac.uk

For peer review only

For peer review only - http://bmjopen.bmj.com/site/about/guidelines.xhtml

Abstract

Background: Most cancers in Sub-Saharan Africa (SSA) are diagnosed at advanced stages, with limited treatment options and poor outcomes. Part of this may be linked to various events occurring in patients' journey to diagnosis. Using the Model of Pathways to Treatment, we examined the evidence regarding the routes to cancer diagnosis in SSA.

Design and settings: A systematic review of available literature was performed

Methods: The PRISMA guidelines were followed. Between September 30th and November 30th 2019, seven electronic databases were searched using terms relating to SSA countries, cancer, and routes to diagnosis; comprising the population, exposure, and outcomes, respectively. Citation lists of included studies were manually searched to identify relevant studies. Furthermore, ProQuest Dissertations & Theses Global was searched to identify appropriate grey literature on the subject.

Results: 18 of 5,083 references identified met the inclusion criteria: eight focused on breast cancer, three focused on cervical cancer, two each focused on lymphoma, Kaposi's sarcoma, and childhood cancers, and one on colorectal cancer. With the exception of Kaposi's sarcoma, definitive diagnoses were made in tertiary healthcare centres, including teaching and regional hospitals. The majority of participants initially consulted within primary care, although a considerable proportion first used alternative medicine before seeking conventional medical help. The quality of included studies was a major concern, but their findings provide important insight into the pathways to cancer diagnosis in the region.

Conclusion: In their journey to diagnosis, patients in SSA mostly consult in primary care or use alternative medicine first. Government and health departments in SSA must find radical solutions to the rising burden of cancer in the region. Investments in sustained cancer awareness programme, research, training and development of primary care and alternative medicine providers to spot and refer suspected cases early, may help improve cancer outcomes in the region.

For peer review only - http://bmjopen.bmj.com/site/about/guidelines.xhtml

For peer review only

Strengths and limitations of this study

- This is the first systematic review of the evidence relating to the routes to diagnosis of cancer in Sub-Saharan Africa
- The search strategies, assessment of quality, and narrative synthesis followed best practice
- Selected studies used small sample sizes and systematically introduced biases in the selection of participants and data collection.
- However, their findings provide unique insights into patients' journey to cancer diagnosis in Sub-Saharan Africa.

For peer review only

For peer review only - http://bmjopen.bmj.com/site/about/guidelines.xhtml

Background

Sub-Saharan Africa (SSA) is overburdened with communicable diseases, whilst the incidence and mortality from non-communicable diseases such as cancer are rising across the region.[1] In 2018, an estimated 1.1million new cancer cases and over 600,000 deaths due to cancer were reported in Africa, based on limited quality data.[2-4] These figures are considerably higher than the estimates for 2012,[5] and are expected to double by 2040, alongside the attendant socio-economic consequences.[3] The increase in cancer incidence is due, in part, to the poor control of cancer-related infections and unhealthy lifestyle choices, some of which may be addressed by implementing effective public health interventions.[6-10]

Mortality from cancer is strongly associated with stage at diagnosis; early-stage cancers enable treatment with curative intent and better prognoses than late-stage diseases.[11-13] Most cancers in SSA are diagnosed at advanced stages, due to late presentation of symptoms, weak referral mechanisms, and limited diagnostic capacity.[12-14] Early-stage cancers and precancerous lesions are detectable by screening asymptomatic patients, but this is limited to few sites and very rarely used in SSA. Therefore, interventions aimed at promoting early symptomatic presentation and expedited diagnosis are likely to yield better cancer outcomes in the region. However, such interventions must be rooted in empirical evidence to ensure effectiveness and maximise local resources use.

The Model of Pathways to Treatment offers a useful framework to examine the routes to diagnosis of symptomatic cancer.[15] It describes five possible events in the pathways to treatment: detection of bodily changes, perceived reasons to seek medical help, first consultation with a healthcare provider, diagnosis, and start of treatment.[15] Numerous studies have explored these events in cancer, but only a few have specifically investigated patients' initial contact with healthcare providers in SSA.[16] Using this framework, we investigated patients' routes to cancer diagnosis in SSA, focusing on the initial point of consultation and eventual diagnosis. Identifying and categorising the routes to diagnosis may explain advanced-stage cancers and provide the basis for early diagnosis interventions in the region.

For peer review only - http://bmjopen.bmj.com/site/about/guidelines.xhtml

Methods

A systematic narrative review was performed. The conduct and reporting of the review was based on the Preferred Reporting Items for Systematic Reviews and Meta-Analysis (PRISMA) framework [see Additional file 1].[17]

Search Strategy

Between September 30th and November 30th 2019, a systematic search of the following electronic databases was performed: Ovid MEDLINE(R) ALL (1946 to September 30, 2019), Embase (1974 to 2019 September 30), Web of Science (1915 (1) - 2019 (69)), PsycINFO (1806 to September Week 2 2019), CINAHL Complete, Global Health (1973 to 2019 Week 36) and African Journals Online (AJOL). The search strategy included terms, their synonyms and MeSH terms relating to SSA countries, cancer, and routes to diagnosis; comprising the population, exposure and outcomes, respectively (Table 1). Additional file 2 shows the search strategy in MEDLINE, PsycINFO, Embase, and Global Health. Citation lists of included studies were manually searched to identify relevant studies. Furthermore, ProQuest Dissertations & Theses Global was searched to identify appropriate grey literature on the subject.

Eligibility criteria

Included studies investigated cancer diagnosis, described the routes or patient's pathway to diagnosis (including the settings of initial consultation and definitive diagnosis), and were conducted in one or more of the 48 SSA countries. The list of SSA countries matches those featured on the World Bank data catalogue used to describe health and socio-economic indices in the region.[18] Excluded studies were non-English studies, focused on populations outside the region of SSA, investigated diseases other than cancer, cancer treatment, outcomes, and attitudes toward cancer diagnoses.

Study Selection

This involved a two-stage screening process. Firstly, title, abstract and full articles of potentially eligible studies were sequentially screened by an experienced researcher (TM) against the inclusion and

For peer review only - http://bmjopen.bmj.com/site/about/guidelines.xhtml

exclusion criteria. Consequently, studies that appeared to meet the inclusion criteria or where a decision could not be made based on the title and/or abstract were selected for full-text review to identify those for the final analysis.

Data Extraction and synthesis

One reviewer (TM) extracted data from all included studies. Extracted data were added to a data extraction spreadsheet, which was initially piloted with seven studies. Data extraction included study characteristics: country of study, design, participants' characteristics, cancer type, health care settings for initial consultation, and eventual diagnosis. Quantitative synthesis was not possible because our final selection differed in terms of cancer sites and outcome measures. For instance, some studies described patients initially presenting to "health care practitioner", a term that may be used to describe primary care physicians or doctors in secondary care. Therefore, we performed a narrative synthesis, using the framework of Rodgers and colleagues.[19] Participants' characteristics and study main findings are illustrated in tables and figures.

Quality assessment

Three reviewers (TM, WH and SM) assessed the methodological quality of eligible studies using the Newcastle-Ottawa Quality Assessment Scale (NOS) for cohort, NOS adapted for cross-sectional studies, [20] [21] and the Joanna Briggs Institute (JBI) Critical Appraisal Checklist for Qualitative Research.[22]TM and SM independently selected the appropriate checklist based on study design. The cohort and cross-sectional studies were awarded stars and rated "good", "satisfactory" or "poor quality", depending on the extent to which they meet the NOS checklists criteria on the three main domains: selection, comparability, outcomes alongside associated statistics. Good-quality studies were awarded four stars in the selection domain, and two stars in each of the comparability and outcome domains. Studies rated satisfactory were awarded two stars in the selection domain, one star in comparability domain, and up to 3 stars in the outcome domain. Poor-quality studies were

For peer review only - http://bmjopen.bmj.com/site/about/guidelines.xhtml

awarded 0 stars in the comparability domain, and one star in the selection or outcome domains. The JBI checklist is not a scoring system but a useful tool for evaluating the risk of bias in the design and conduct of qualitative studies. The checklist consists of 10 criteria with four possible responses: "yes," "no," "unclear," and "inapplicable." Each qualitative study was evaluated against the checklist criteria. Discrepancies between the reviewers were resolved by consensus, although no study was excluded based on quality.

Patient and Public Involvement

There was no formal patient and public involvement in this review.

Result

Study characteristics

The search identified 5,083 articles. After screening title and abstract, and removing duplicates, 4,933 irrelevant articles were excluded: 150 full text articles were assessed with 18 meeting the inclusion criteria. A PRISMA flowchart showing the reasons for abstract and full article exclusions is shown in Figure 1. The 18 studies recruited a total of 4,871 participants from nine SSA countries, 70% of which were females with the average age ranging from 4 to 59 years. The characteristics of included studies are illustrated in Table 2, with the results of quality assessment in Table 3. Seven of the studies were conducted in Nigeria,[23-29] three in Ethiopia,[30-32] two each in Ghana,[33 34] and South Africa,[35 36] and one each in Cameroon,[37] Tanzania,[38] and Kenya.[39]The final study involved five countries (Kenya, Uganda, Malawi, Cameroon and Nigeria).[40]

All 18 studies were observational with seven cross-sectional surveys, seven cohorts (using medical records), three qualitative (face-to-face interviews), and a mixed-methods study (using both qualitative and quantitative data). Eight studies examined breast cancer,[23-26 30 31 33 34] three focused on cervical cancer,[27 32 38]two each focused on lymphoma,[36 37] Kaposi's sarcoma,[35 40] and childhood cancers,[29 39] and one on colorectal cancer.[28] None of the 18 studies specifically investigated the routes

For peer review only - http://bmjopen.bmj.com/site/about/guidelines.xhtml

to cancer diagnosis, although 15 studies reported the settings of initial consultation after symptoms onset. The remaining three studies recruited participants from primary care-based HIV clinics to investigate Kaposi's sarcoma and lymphoma diagnoses.[35 36 40] These studies were included in our final selection given that both cancer types are significantly more common in HIV patients and that HIV patients are mostly seen at such settings.

Assessment of study quality

Overall, none of the qualitative studies fulfilled the JBI checklist criteria and none of the quantitative studies could be classified as ”good quality” due to the limitations in their methodology (Table 3). The main flaws in these studies pertained to their small sample sizes, biases in participant recruitment, and data collection strategies. The sample sizes in most of the cohort and cross-sectional studies were rather small to be representative of the target population. Four-fifth of included studies recruited participants from tertiary healthcare centres, thereby introducing selection bias by systematically excluding patients diagnosed or treated elsewhere. In some studies, surveys and face-to-face interviews were performed by nurses or physician-researchers from the hospitals where participants were receiving treatment, thus drawing possibly desirable responses. Additionally, statistical analyses were largely descriptive, with most studies presenting percentages only. Despite these flaws, however, the studies provided some important findings relevant to the aim of our review, thereby warranting their inclusion in the synthesis.

Routes to cancer diagnosis

Across the eight studies on breast cancer, providers in tertiary healthcare centres made the definitive diagnoses in all cases (Table 2).[23-26 30 31 33 34] After noticing symptoms, participants initially consulted the physicians (in primary or secondary care), used alternative medicine (including traditional healers, herbalists, and prayer centres), or presented directly to the hospital. The proportion of patients using each of these routes to diagnosis differed slightly between studies but very similar across all the eight studies.[23-26 30 31 33 34] On average, around a third of the participants - across the studies - initially

For peer review only - http://bmjopen.bmj.com/site/about/guidelines.xhtml

presented symptoms to each of the physician, alternative medicine practitioners, or directly to the hospital.

In two of the three studies focused on cervical cancer, participants presented with symptoms directly to tertiary health centres where cervical cancer diagnoses were confirmed. (Table 2).[27 32] Conversely, 47% of participants in the third study initially presented symptoms to traditional healthcare practitioners before returning to the tertiary health centres for diagnosis and start of treatment.[38]

In a survey of 82 patients with rectal bleeding and colorectal cancer, Alatise *et al.* found that only 39% of the participants had consulted a physician, with 38% of participants opting to use herbs before going to the doctors (Table 2).[28]

Of 6,292 HIV infected patients enrolled at an HIV clinic, Chu *et al.* found 3% diagnosed with Kaposi's sarcoma within seven years of routine HIV care.[35] Similarly, healthcare providers from 33 HIV clinics across five African countries diagnosed 1,328 HIV patients with Kaposi's sarcoma during four years of routine HIV care.[40] In both studies, providers at the HIV clinics detected Kaposi's sarcomas during routine examination for opportunistic infections.

Two studies surveyed parents and carers of children with childhood cancers to determine causes of diagnostic delay. In one study, 59% of parents initially sought alternative medicine for their children, although about 60% later consulted in primary care, 38% in secondary care, and 2% presented directly to tertiary care.[39] In contrast, 69% of parents in the second study initially sought conventional medical help, but 24% either self-medicated, used herbalist services or presented to a church.[29]

In a survey of parents and carers of children with Burkitt lymphoma, Afungchwi and colleagues showed that 55% had used traditional healers before hospital admission, with 42% using this service before reporting to primary care.[37] In contrast, all 163 patients diagnosed with Hodgkin and Non-Hodgkin lymphoma in Antel *et al's* study were referred to the specialist by healthcare practitioners.[36]

For peer review only - http://bmjopen.bmj.com/site/about/guidelines.xhtml

Discussion

The route to diagnosis is a strong predictor of cancer outcomes.[41 42] In this review, we examined the evidence relating to cancer diagnosis in SSA. Across all selected studies, definitive diagnoses of cancer were made by specialists in large tertiary healthcare centres, except for Kaposi's sarcomas, which were diagnosed at various primary care-based specialist clinics. However, participants' journeys to the specialist clinics are often indirect, with a considerable proportion initially using alternative medicine before consulting conventional medical services.

Strength and limitations

To our knowledge, this is the first systematic review of the evidence regarding the routes to cancer diagnosis in SSA. Our rigorous search strategy and explicit inclusion/exclusion criteria, quality assessment of included studies and narrative synthesis followed best practice. Our search identified only a modest number of studies, a third of which were conducted in Nigeria, which is the most populous country with the largest economy in the region. We omitted the British and French Overseas Territories and few countries (Egypt, Morocco, Algeria, Tunisia, and Libya), which are usually classed as part of the Arab world. Health services in some of these countries are similar to those of the developed world, providing universal care through social or government contributions.[43 44] Healthcare services in many SSA countries are not universally accessible. They are pluralistic with a range of public and private providers who barely communicate with each other. Additionally, our search strategies omitted non-English studies, thereby excluding any studies published in French from the small number of Francophone countries in SSA. The decision to omit these countries in our search strategy may have reduced the number of selected studies slightly, but we have no reason to believe that such omission had any impact on our findings.

A limitation was that almost half of the studies focused on breast cancer, reducing the scope of the review. Also, we may have omitted some studies reporting cancer emergencies or direct symptomatic presentations to tertiary healthcare. Importantly, any review is only as good as the studies it finds.

For peer review only

Our final selection had small sample sizes, which limits interpretation and generalisability. The majority also recruited participants and gathered data (using researcher-administered questionnaires) from the hospital facilities where patients were being treated for their cancers, typically in the tertiary healthcare centres. This is not surprising, with weak primary care services and the absence of established cancer registries in SSA, thus limiting the quality and quantity of data available for research. However, recruiting participants from tertiary health centres systematically excludes patients treated in private hospitals and those whose cancers may never be found due to affordability or comorbidity. Furthermore, gathering data from the hospital using physician-administered questionnaires may generate more socially desirable responses. In this case, it is likely that participants under-report their use of alternative medicine and self-medication to look good in the eyes of their providers who may be part of the research team.

Finally, publication bias is possible as some studies on the subject may have failed to be published in reputable peer-reviewed journals, and so would have been omitted from the databases searched for this review.

Interpretation of findings

The pathways to diagnosis of symptomatic cancer involves a series of events, beginning with the patient noticing a bodily change and deciding to seek medical help.[15] Definitive diagnosis requires biopsy of affected tissue by specialists in secondary or tertiary healthcare settings. In developed countries like the UK and Denmark, most cancer patients initially present with symptoms to primary care, with a smaller proportion presenting to secondary care as emergencies.[41] Primary care physicians in these countries play a key role in selecting those whose symptoms warrant specialist investigations using preliminary test results and clinical guidelines.[45] Healthcare services in many SSA countries consist of a three-tier system: primary care (including dispensaries, health centres and private clinics); secondary care (including private, mission and district hospitals); and tertiary healthcare.[30] The tertiary healthcare centres are referral centres with various sub-specialities, and are

For peer review only - http://bmjopen.bmj.com/site/about/guidelines.xhtml

the main setting for definitive diagnosis of cancer.[30 32 34 46] However, the role of primary care in SSA is not always well-defined, with several unorthodox providers including traditional healers and faith clinics, offering similar services, albeit unqualified to diagnose cancer or to refer patients for specialist investigations. [13 47-49] Patients in these countries may present with symptoms directly to tertiary healthcare centres, regardless of the nature or duration of symptoms. They may also be referred by physicians in primary or secondary care, but often with no standardised referral pathways or mechanism to ensure continuity of care. [13 47-49] This problem is further compounded by frequent long distances to healthcare centres and out-of-pocket payments, particularly for patients in rural and deprived areas – who may resort to alternative medicine instead.

Indeed, a considerable proportion of participants in this review initially used alternative medicine before consulting in primary care, with some also presenting directly to the hospital. Only a third of women with breast cancer initially reported symptoms to primary care, despite widespread awareness campaign with relatively easy to spot symptoms.[50 51] 53% of patients with cervical cancer symptoms, 39% of those with rectal bleeding, and around two-third of childhood cancers initially sought help in primary care. Access to conventional health care is restricted in most SSA countries due to limited availability and affordability.[13 48 52 53] In their respective cancer journey, patients in this region may start with or revert to alternative medicine, which is considered cheaper and more natural, with some practitioners offering complete cure of cancer rather than possible remission offered by conventional medicine.[30 47] The use of alternative medicine is widespread in SSA, though not as fully developed compared to the practice in Asia and North America.[54-56] Yet, a recent study showed that the common diagnosis of Burkitt lymphoma - among alternative medicine practitioners - included liver problem, abscess, witchcraft, poison, hernia, side pain, mushroom in the belly, and toothache.[37] Use of alternative medicine is associated with advanced-stage diagnosis and poorer survival of major cancer types,[57] and so may, in part, explain the poorer outcomes of cancer in SSA.

For peer review only - http://bmjopen.bmj.com/site/about/guidelines.xhtml

The findings of this review may have been influenced by the level of bias in included studies: in which case, our report on the proportion using various route to diagnosis will be inaccurate. If at all, we may have overestimated the proportion of patients consulting in primary care or underestimated those using alternative medicine before diagnosis, given the lack of public awareness of cancer and weakness of healthcare systems in the region, with significant underdiagnoses.

Conclusion

Recent data from SSA suggests a rapid increase in the risk and deaths from major cancer types. In a region where infectious diseases persist, with limited healthcare budgets and shortages of specialists, urgent solutions are required to minimise the burden of cancer on its rapidly growing and ageing population. The majority of participants in our selected studies initially presented symptoms to primary care, though the proportion first using alternative medicine is considerable. This latter group of patients constitutes a major source of concern, bearing in mind that alternative medicine practitioners in SSA are likely to be unequipped to spot cancer or make a specialist referral when necessary. Government and health departments in SSA must consider ways of engaging alternative medicine providers within the mainstream health system to ensure that patients with suspected cancers are diagnosed are not falsely reassured, and their cancer missed when curative treatments can be offered. Acknowledging the challenges that such engagement may pose, ignoring the role of this major care provider in SSA will have a negative impact on patients' route to diagnosis of cancer and other non-communicable diseases.

There is a need for further research to fully understand patients' pathways to cancer diagnosis in SSA. For instance, our review found that the majority of patients initially presented in primary care, but we are uncertain the exact roles this played in their journey to diagnosis. As such, a comprehensive research programme to examine the role of primary care in cancer diagnosis is recommended as this may contribute to the development of possible referral guidelines in primary care. Such programmes

For peer review only - http://bmjopen.bmj.com/site/about/guidelines.xhtml

should investigate consultation patterns, the profile of symptoms presented and investigations performed in primary care before specialist referral. Beside research, there is need for a comprehensive cancer awareness programme to improve public knowledge and providers' (including alternative medicine practitioners) ability to spot the disease in SSA.

For peer review only

For peer review only - http://bmjopen.bmj.com/site/about/guidelines.xhtml

Table 1: Search terms

Population	Exposure	Outcome
**Terms relating Sub-Saharan Countries:**<br>Angola, Gabon, Nigeria, Benin, Gambia, The Rwanda, Botswana, Ghana, São Tomé and Principe, Burkina Faso, Guinea, Senegal, Burundi, Guinea-Bissau, Seychelles, Cabo Verde, Kenya, Sierra Leone, Cameroon, Lesotho, Somalia, Central African Republic, Liberia, South Africa, Chad<br>Madagascar, South Sudan, Comoros, Malawi, Sudan, Congo, Dem. Rep., Mali, Swaziland, Congo Rep., Mauritania, Tanzania, Côte d'Ivoire, Mauritius, Togo, Equatorial Guinea, Mozambique, Uganda, Eritrea, Namibia, Zambia, Ethiopia, Niger, Zimbabwe	**Terms relating to Cancer:**<br>Cancer, Neoplasm, Malignant Neoplasm, tumour, Malignant tumour, Astrocytoma, Adenocarcinoma, Glioma, Mesothelioma, Medulloblastoma, Myeloma, Melanoma, Neuroblastoma, Sarcoma, Nonmelanoma, Osteosarcoma, Teratoma, Seminoma, Hodgkin, Leukaemia, Lymphoma, Retinoblastoma	**Terms relating to the routes to Cancer Diagnosis:**<br>Pathway to diagnosis<br>Pathway to detect*<br>Routes to diagnos*<br>Routes to detect*<br>Diagnos*<br>Detect*<br>Consult*<br>Help-seek*<br>Present*<br>Route to consult*<br>Routes to present*<br>Pathway to consult*<br>Pathway to present*<br>Primary care<br>Family doctor<br>Physician<br>Health care practitioner<br>General Practitioners<br>Family Practice<br>Primary Health Care

For peer review only

Table 2: Study characteristics

Author	Country	Type/site	Title	Method	Outcome measure	Sample characteristics	Relevant findings
Dye *et al.* 2010[30]	**Ethiopia**	**Breast**	Complex care systems in developing countries: breast cancer patient navigation in Ethiopia	A mixed-methods study using semi-structure interview to investigate participant's navigation through the health system before arriving at the tertiary health centre for treatment.	Patient navigation through the health care system that culminated in the treatment of cancer	**Participants:** 55 patients with breast cancer plus 14 carers.<br>**Mean age:** 45.5 years<br>**Sex:** 98% females	Initial presentation of symptoms was:<br>53.7% to primary care<br>16.4% to traditional healers<br>16.4% to local/regional hospital<br>9% to private hospital<br>4.5% directly to the tertiary referral centre.<br>Definitive diagnoses were made at the tertiary health centre.
Jemebere 2019[31]	**Ethiopia**	**Breast**	Barriers Associated with Presentation Delay among Breast Cancer Patients at Hawassa University Comprehensive and Specialized Hospital, Southern Ethiopia	Cross-sectional survey of women diagnosed with breast cancer at a specialised hospital	Route to diagnosis	**Participants:** 106 women<br>**Age range:** 15 - 65years.<br>**Occupation:** Farmers (3%), Labourer (9%), Merchant (16%), Professional 29%) and Housewife (43%).<br>**Education:** None (28%)<br>Elementary (29%), High (24%) and College≥ (19%).<br>Family history of breast cancer (13%)	64% delayed presenting to the hospital due to initial use of alternative medicine including: herbal remedy, traditional healers and prayers.<br><br>Definitive diagnoses were made at the teaching hospital.
Ezeome *et al.* 2010[23]	**Nigeria**	**Breast**	Delays in presentation and treatment of breast cancer in Enugu, Nigeria	Cross-sectional survey of breast cancer patients at an oncology specialist unit (in a teaching hospital).	Patients first point of symptom(s) presentation<br><br>Patients first point of conventional medical treatment	**Participants:** 164 patients (162 female & 2 males) **Median (range) age:** 45 (21-77) years<br>**Socioeconomic status:** Low (59%), Middle (40%), High (1%)<br>**Education:** None (15%), Primary (24%), Secondary (29%), Degree (30%)<br>**Religion:** 96% were Christians	13.1% first used traditional healers<br>4.4% first presented to a prayer house.<br>82.3% initially presented to health care facilities.<br>First point of contact with medical facilities:<br>50% within primary (GP)<br>25% to consultant surgeon<br>10.1% to patent medicine dealer<br>7.5% to a gynaecologist<br>5% to a nurse or allied health professional
Pruitt *et al.* 2015[24]	**Nigeria**	**Breast**	Social barriers to diagnosis and treatment of breast cancer in patients presenting at a teaching hospital in Ibadan, Nigeria	A qualitative study which used semi-structured interview of patients with breast cancer in a teaching hospital.	Help-seeking behaviour after noticing symptoms.	**Participants:** 31 women with breast cancer.<br>**Median (range) age:** 51 (28-80≥)years<br>**Education:** None (n=7), Primary/Secondary (n= 15) Tertiary (n=9).<br>**Religion:** Christians (83%) and Muslim (17%)	Most women rapidly sought orthodox medical care once they noticed symptoms, however few reported seeking herbal/spiritual help initially.<br><br>Definitive diagnoses were made at the teaching hospital.
Adesunkanmi *et al. 2006*[25]	**Nigeria**	**Breast**	The severity, outcome and challenges of breast cancer in Nigeria	A retrospective study using 8 years records of patient with breast cancer diagnosis in a tertiary health centre.	To determine the challenges of breast cancer diagnosis at the centre.	**Participants:** 212 patients<br>**Sex:** 99.5% female<br>**Mean (SD) age:** 48±12.3 years.<br>**Occupation:** Traders (52%), Teachers (31.6%), Nurses (5.5%), Farmers (4%), and Self-employed (1.4%).<br>**Education:** Primary (18%), Secondary (14%) and Tertiary (35%).<br>Previous breast disease (25%) Family history of breast cancer (7.2%)	○ 92% of the tumour were self-detected<br>○ 4.2% by physicians<br>○ and 3.8% by partners<br>○ Definitive diagnoses of all cases were made at the tertiary health centre

For peer review only - http://bmjopen.bmj.com/site/about/guidelines.xhtml

Ahmed *et al.* 2012[26]	**Nigeria**	**Breast**	Management and Outcomes of Male Breast Cancer in Zaria, Nigeria	A retrospective study using 10 years medical records of men with breast cancer in a specialist oncology centre (in a teaching hospital).	Route to diagnosis	**Participants:** 57 men<br>**Mean (SD) age:** 59 ±2.3 years	○ Definitive diagnoses were made at the specialist centre.<br>○ 21% initially consulted traditional healers before presenting to the specialist.<br>○ 49% first presented symptoms to the specialist.
Aziato *et al.* 2015[33]	**Ghana**	**Breast**	Breast Cancer Diagnosis and Factors Influencing Treatment Decisions in Ghana	A qualitative study using face-to-face interviews of breast cancer patients from a Surgical Unit and breast cancer support group.	Route to diagnosis	**Participants:** 12 female<br>**Age range:** 31-60 years<br>**Religion:** All were Christians	Women self-identified breast lesion or accidently during medical examination for other problems.<br>Participants who self-identified breast lesion presented directly to the tertiary health centre where definitive diagnoses were made.
Agbokey *et al. 2019[34]*	**Ghana**	**Breast**	Knowledge and Health Seeking Behaviour of Breast Cancer Patients in Ghana	A qualitative study using in-depth interviews to examine help-seeking behaviour of female breast cancer patients at a teaching hospital	Patient help-seeking behaviour after noticing female breast symptoms	**Participants:** 20 females<br>**Median (range) age:** 52.5 (29-80) years<br>**Occupation:** Traders (n=11), Teachers (n=3) Farmers (n=5) and Nurse (n=1).<br>**Education: none (n=4)** Primary (n=12), Secondary (n=1), and Tertiary (n=3).<br>**Religion:** 95% were Christians	12/20 first sought unorthodox care (herbalist, drug stores, home remedies and prayer camps) after noticing symptoms. Some patient went through a cycle of hospital-to-herbalist-and back to hospital care before diagnosis.<br>In all of the cases, however, definitive diagnoses were made at the tertiary health centre
Mlange *et al. 2016[38]*	**Tanzania**	**Cervical**	Patient and disease characteristics associated with late tumour stage at presentation of cervical cancer in north-western Tanzania	A cross-sectional survey of women with histologically confirmed cervical cancer at a tertiary health centre	Route to diagnosis	**Participants:** 202 women with cervical cancers<br>**Mean (SD) age:** 50±11 years<br>**Education:** None (57%) Primary (39%), Secondary (2.4%), and College (1.4%).<br>**Occupation:** Farmers (84%), Trader (9.9%), Employed (2.4%), Business (0.9%) and Unemployed (2.4%)	○ 95 (47%) initially presented to traditional health practitioner.<br>○ Presenting to a traditional health practitioner was strongly associated with late-stage diagnosis (Odds Ratio = 2.3 [95% CI 1.2–4.2], p = 0.011)<br>○ Definitive diagnosis were made at the tertiary health centre.
Begoihn *et al.* 2019[32]	**Ethiopia**	**Cervical**	Cervical cancer in Ethiopia – predictors of advanced stage and prolonged time to diagnosis	A retrospective cohort study of patients diagnosed with primary cervical cancer at a tertiary health centre	Route to diagnosis of cervical cancer	**Participants:** 1,575 women with cervical cancers<br>**Mean (SD) age:** 48.9±11.5 years	○ All of the 1,575 women presented cervical cancer symptoms to the tertiary health centre.
Eze *et al.* 2013[27]	**Nigeria**	**Cervical**	A Six-Year Study of the Clinical Presentation of Cervical Cancer and the Management Challenges Encountered at a State Teaching Hospital in Southeast Nigeria	A retrospective cohort study of patients diagnosed with primary cervical cancer at a tertiary health centre	Route to diagnosis of cervical	**Participants:** 61 women with primary cervical cancers. **Mean (SD) age:** 54±12.7 years**. Education:** None (36.1%) Primary (39.3%), Secondary (23%), and College (1.6%).<br>**Occupation:** Farmers (60%), Trader (37.7%), Dependent (36.1%), Business (0.9%) and Retired (6.6%)	○ All of the 61 women presented cervical cancer symptoms to the tertiary health centre.
Alatise *et al.* 2017[28]	**Nigeria**	**Colorectal**	Health-Seeking Behaviour and Barriers to Care in Patients With Rectal Bleeding in Nigeria	A prospective survey of patients with rectal bleeding in the general population.	Attitude about seeking expert opinion among patient with rectal bleeding.<br><br>Initial help-seeking after the onset of rectal bleeding	**Participants:** 82 patients with rectal bleeding<br>**Median (range) age:** 45 (18-85) years<br>**Sex:** 78% were males<br>**Education:** Primary (28%), Secondary (33%), tertiary (30%)<br>**Religion:** Christians (66%) and Muslims (33%)	○ 39% of the participants consulted a physician with rectal bleeding.<br>○ 38% suggested that herbs should be used before seeing a physician.<br>○ Patients who scored high on knowledge of rectal bleeding were more likely to consult the physician (Odds ratio: 3.82; 95% CI, 55-10.2).

For peer review only - http://bmjopen.bmj.com/site/about/guidelines.xhtml

Chu *et al.* 2010[35]	**South Africa**	**Kaposi's Sarcoma**	AIDS-associated Kaposi's sarcoma is linked to advanced disease and high mortality in a primary care HIV programme in South Africa	Analysis of data from a cohort study of patients with AIDS-associated Kaposi's sarcoma in primary care	Patient pathway to diagnosis of Kaposi's Sarcoma	**Participants:** 215 patients with Kaposi's Sarcoma<br>**Median (interquartile range) age:** 34 (29 -41) years<br>**Sex:** 41% were females	189/6292 patients enrolled at the HIV Clinic were diagnosed with AIDS-associated Kaposi's sarcoma during routine examination.
Freeman *et al.* 2016[40]	**Kenya<br>Uganda<br>Malawi<br>Nigeria<br>Cameroon**	**Kaposi's Sarcoma**	Pitfalls of practicing cancer epidemiology in resource-limited settings: the case of survival and loss to follow-up after a diagnosis of Kaposi's sarcoma in five countries across sub-Saharan Africa	Analysis of HIV-infected patients' in primary care records across five countries.	Route to diagnosis of Kaposi's Sarcoma	**Participants:** 1,328 patients with Kaposi's Sarcoma **Median (interquartile range) age:** 35 (30 -41) years<br>**Sex:** 40% were females	During routine examination for AIDS-related infections at the HIV clinic, 1,328 patients were diagnosed with Kaposi's sarcoma across the five countries between 2009 and 2012
Afungchwi *et al.* 2017[37]	**Cameroon**	**Burkitt Lymphoma**	The role of traditional healers in the diagnosis and management of Burkitt lymphoma in Cameroon: understanding the challenges and moving forward	A survey of parents and carers of children diagnosed with Burtkitt lymphoma in three large hospitals	Route to diagnosis Burkitt Lymphoma	**Participants:** 384 questionnaire completed<br>**Median (range) age:** 8 (1 -15) years.<br>**Sex:** Males (57.4%) and females (42.4%)<br>**Religion:** Christians (68.9%) and Muslims (30%)	○ Overall, 55% of parents used traditional healers before hospital admission<br>○ 41.8% first consulted traditional healers before reporting at local health centre.
Antel *et al.* 2019[36]	**South Africa**	**Lymphoma**	The determinants and impact of diagnostic delay in lymphoma in a TB and HIV endemic setting	A retrospective cohort study of patients diagnosed with lymphomas. Data sources included hospital records, telephone and face-to-face interviews.	Route to diagnosis of Hodgkin and Non-Hodgkin lymphoma	**Participants:** 163 HIV patients<br>**Median (range) age:** 48 (15-86) years<br>**Sex:** 58% were males<br>**Socioeconomic status:** 70% on social grant or <251 monthly income	All 163 HIV patients were diagnosed with Hodgkin (41) and Non-Hodgkin (122) lymphoma. They were referred to the tertiary health centre by healthcare practitioners
Brown *et al.* 2015[29]	**Nigeria**	**Childhood**	A Prospective Study on the Causes of Delayed Diagnosis of Childhood Cancer in Ibadan, Nigeria	A survey of parents and carers of children diagnosed with malignant tumour in a tertiary health care settings	Factors influencing pre-diagnostic intervals among parent/carers of patient with childhood cancers	**Participants:** 91 children with cancer<br>**Median (range) age:** 4years (1month -15years).<br>**Sex:** 50.5% were males	○ 69% of parent initially sought medical help for their children within a health facility<br>○ 19% self-medicated<br>○ 4% used herbalist,<br>○ 3% consulted a patent medicine dealers<br>○ 2% presented to a nurse/health worker and 1% visited a church.<br>Health facilities users comprised: 69% Public hospital, 31% Private
Njuguna *et al.* 2016[39]	**Kenya**	**Childhood**	Factors influencing time to diagnosis and treatment among paediatric oncology patients in Kenya	A cross-sectional survey of parent and carers of 99 children diagnosed with a malignancy.	Help-seeking after the onset of symptoms.	**Participants:** 99 children with cancer<br>**Median age:**<br>Children: 5.7years<br>Mother: 31 (19-56) years.<br>**Sex:** 67% were male.<br>**Religion:** 99% of mothers were Christians.<br>**Employment:** Farmers (29%), *Regular jobs (24%),* Casual labourers (6%) and Unemployed (6%)	58 (59%) of parent initially sought alternative treatment for their children, including: praying ceremonies (41%), visiting herbalist (36%), special food intake (11%), and attending traditional healer (3%).<br>First contact with conventional health care facilities included: 60% in primary care, 38% in secondary, and 2% in tertiary health care.

Note: SD Standard Deviation; CI Confidence Interval

For peer review only

Table 3: Quality of studies

Study quality and score based on NOS for cohort studies

**Author, cohort studies**	**Selection**				**Comparability**	**Outcomes and associated statistical analysis**			
	Representativeness of the exposed cohort (★)	*Selection of the non-exposed cohort* (★)	*Ascertainment of exposure* (★)	*Demonstration that outcome of interest was not present at start of study* (★)	*Comparability of cohorts on the basis of the design or analysis* (★★)	*Assessment of outcome* (★)	*Was follow-up long enough for outcomes to occur* (★)	*Adequacy of follow up of cohorts* (★)	*overall quality*
Ahmed *et al.*,2012[26]	★	-	-	★	★	★	★	-	Sat
Adesunkanmi *et al., 2006*[25]	★	-	★	★	-	★	★	-	Poor
Begoihn *et al.,* 2019[32]	★	-	★	-★	★	★	-★	-	Sat
Chu *et al., 2010*[35]	★	-	★	★	★	★	★	-	Sat
Freeman *et al.*,2016[40]	★	-	★	★	★	★	★	-	Sat
Eze *et al.,* 2013[27]	★	-	★	★	-	★	-	-	Poor
Antel *et al.,* 2019[36]	★	-	★	★	★	★	★	-	Sat

Study quality and score based on NOS adapted for cross-sectional studies

**Author, cross-sectional studies**	*Representativeness of the sample* (★)	*Justification of sample size* (★)	*Non-respondents* (★)	*Ascertainment of exposure* (★★)	*Comparability of subjects* (★★)	*Assessment of outcome* (★★)	*Statistical test* (★)	*Overall quality*
Jemebere, 2019[31]	★	-	★	★	-	★	-	Poor
Ezeome *et al.,* 2010[23]	★	-	-	★	-	★	-	Poor
Mlange *et al., 2016*[38]	★	-	★	★	★	★	★	Sat
Alatise *et* al., 2017[28]	★	-	-	★	-	★	-	Poor
Afungchwi *et al.,* 2017[37]	★	-	-	★	-	★	-	Poor
Brown *et al.,* 2015[29]	★	-	-	★	★	-	-	Poor
Njuguna *et al.,* 2016[39]	★	-	★	★	★	★	-	Sat

Study quality and scores based on the JBI Checklist for Qualitative Research

Authors, qualitative studies	*Congruity between stated philosophical perspective and the research methodology?*	*Congruity between research method and the research question or objectives?*	*Congruity between research method and the methods used to collect data?*	*Congruity between research method and representation and analysis of data?*	*Congruity between method & interpretation of results?*	*Statement locating the researcher culturally or theoretically?*	*Influence of the researcher on the research, and vice- versa, addressed?*	*Participants' voices adequately represented?*	*Evidence of ethical approval?*	*Conclusions report flow from the analysis, or interpretation, of the data?*
Dye *et al.* 2010[30]	Unclear	Yes	Yes	Yes	Yes	Unclear	No	Yes	Yes	Yes
Pruitt *et al. 2015*[24]	Unclear	Yes	Yes	Yes	Yes	Unclear	No	Yes	Yes	Yes
Aziato *et al.* 2015[33]	Unclear	Yes	Yes	Yes	Yes	Unclear	No	Yes	Yes	Yes
Agbokey *et al. 2019*[34]	Unclear	Yes	Unclear	Yes	Yes	Unclear	No	Yes	Yes	Yes

Note: Sat satisfactory-quality paper

For peer review only - http://bmjopen.bmj.com/site/about/guidelines.xhtml

Competing interests: The authors declare that they have no competing interests.

Authors' contributions: TM was involved in all aspects. WH participated in the study design, data interpretation and preparation and revision of the manuscript. SM participated in the assessment of studies quality and revision of the manuscript. All authors read and approved the final manuscript.

Ethical approval: Not applicable.

Acknowledgements: Not applicable

Funding: TM receives funding from Cancer Research UK (CRUK) Population Research Committee [C56361/A26124]. SM is supported by the CanTest Collaborative, which is funded by Cancer Research UK [C8640/A23385]. WH is co-Directors of CanTest.

Data availability statement: No additional data available

Figure 1: Flow chart of study selection process

For peer review only

References

1. Gouda HN, Charlson F, Sorsdahl K, et al. Burden of non-communicable diseases in sub-Saharan Africa, 1990–2017: results from the Global Burden of Disease Study 2017. *The Lancet Global Health* 2019;7(10):e1375-e87. doi: 10.1016/S2214-109X(19)30374-2
2. Global Cancer Observatory. Estimated number of incident cases from 2018 to 2040, all cancers, both sexes, all ages 2018 [cited 2019 03/10]. Available from: http://gco.iarc.fr/tomorrow/graphic-line?type=0&population=900&mode=population&sex=0&cancer=39&age_group=value&apc_male=0&apc_female=0.
3. Observatory GC. Estimated number of deaths from 2018 to 2040, all cancers, both sexes, all ages 2018 [cited 2019 03/10]. Available from: http://gco.iarc.fr/tomorrow/graphic-isotype?type=1&population=900&mode=population&sex=0&cancer=39&age_group=value&apc_male=0&apc_female=0.
4. Busolo DS, Woodgate RL. Cancer prevention in Africa: a review of the literature. *Glob Health Promot* 2015;22(2):31-9. doi: 10.1177/1757975914537094 [published Online First: 2014/07/17]
5. Ferlay J, Soerjomataram I, Dikshit R, et al. Cancer incidence and mortality worldwide: Sources, methods and major patterns in GLOBOCAN 2012. *Int J Cancer* 2015;136(5):E359-E86. doi: 10.1002/ijc.29210
6. The World Bank. Disease and Mortality in Sub-Saharan Africa. Washington (DC): The International Bank for Reconstruction and Development/The World Bank. 2006.
7. Azubuike SO, Muirhead C, Hayes L, et al. Rising global burden of breast cancer: the case of sub-Saharan Africa (with emphasis on Nigeria) and implications for regional development: a review. *World J Surg Oncol* 2018;16(1):63. doi: 10.1186/s12957-018-1345-2
8. Musgrove DTJJGBARMGAMCDBEPJAMP. Disease Control Priorities in Developing Countries (Second Edition).
9. Brawley OW. The role of government and regulation in cancer prevention. *The Lancet Oncology* 2017;18(8):e483-e93. doi: https://doi.org/10.1016/S1470-2045(17)30374-1
10. Plummer M, de Martel C, Vignat J, et al. Global burden of cancers attributable to infections in 2012: a synthetic analysis. *The Lancet Global Health* 2016;4(9):e609-e16. doi: 10.1016/S2214-109X(16)30143-7
11. McPhail S, Johnson S, Greenberg D, et al. Stage at diagnosis and early mortality from cancer in England. *Br J Cancer* 2015;112 Suppl 1(Suppl 1):S108-S15. doi: 10.1038/bjc.2015.49
12. Jedy-Agba E, McCormack V, Adebamowo C, et al. Stage at diagnosis of breast cancer in sub-Saharan Africa: a systematic review and meta-analysis. *The Lancet Global Health* 2016;4(12):e923-e35. doi: https://doi.org/10.1016/S2214-109X(16)30259-5
13. Olaleye O, Ekrikpo U. Epidemiology of Cancers in Sub-Saharan Africa. In: Adedeji OA, ed. Cancer in Sub-Saharan Africa: Current Practice and Future. Cham: Springer International Publishing 2017:3-19.
14. Cassim S, Chepulis L, Keenan R, et al. Patient and carer perceived barriers to early presentation and diagnosis of lung cancer: a systematic review. *BMC Cancer* 2019;19(1):25. doi: 10.1186/s12885-018-5169-9 [published Online First: 2019/01/10]
15. Scott SE, Walter FM, Webster A, et al. The Model of Pathways to Treatment: Conceptualization and integration with existing theory. *British Journal of Health Psychology* 2013;18(1):45-65. doi: 10.1111/j.2044-8287.2012.02077.x
16. Khakbazan Z, Taghipour A, Latifnejad Roudsari R, et al. Help seeking behavior of women with self-discovered breast cancer symptoms: a meta-ethnographic synthesis of patient delay. *PLoS ONE* 2014;9(12):e110262-e62. doi: 10.1371/journal.pone.0110262
17. Moher D, Shamseer L, Clarke M, et al. Preferred reporting items for systematic review and meta-analysis protocols (PRISMA-P) 2015 statement. *Systematic Reviews* 2015;4(1):1. doi: 10.1186/2046-4053-4-1

For peer review only - http://bmjopen.bmj.com/site/about/guidelines.xhtml

18. The World Bank. Data Catalog: Regions and Countries 2019 [Available from: https://datacatalog.worldbank.org/ accessed 10/10 2019.
19. Rodgers M, Sowden A, Petticrew M, et al. Testing methodological guidance on the conduct of narrative synthesis in systematic reviews: effectiveness of interventions to promote smoke alarm ownership and function. *Evaluation* 2009;15(1):49-73.
20. GA Wells BS, D O'Connell, J Peterson, V Welch, M Losos, P Tugwell,. The Newcastle-Ottawa Scale (NOS) for assessing the quality of nonrandomised studies in meta-analyses [03/08/2020]. Available from: http://www.ohri.ca/programs/clinical_epidemiology/oxford.asp accessed 22/07/2020.
21. Modesti PA, Reboldi G, Cappuccio FP, et al. Panethnic Differences in Blood Pressure in Europe: A Systematic Review and Meta-Analysis. *PLoS ONE* 2016;11(1):e0147601. doi: 10.1371/journal.pone.0147601
22. Lockwood C, Munn Z, Porritt K. Qualitative research synthesis: methodological guidance for systematic reviewers utilizing meta-aggregation. *Int J Evid Based Healthc* 2015;13(3):179-87. doi: 10.1097/xeb.0000000000000062 [published Online First: 2015/08/12]
23. Ezeome ER. Delays in presentation and treatment of breast cancer in Enugu, Nigeria. *Niger J Clin Pract* 2010;13(3):311-6.
24. Pruitt L, Mumuni T, Raikhel E, et al. Social barriers to diagnosis and treatment of breast cancer in patients presenting at a teaching hospital in Ibadan, Nigeria. *Glob Public Health* 2015;10(3):331-44. doi: https://dx.doi.org/10.1080/17441692.2014.974649
25. Adesunkanmi AR, Lawal OO, Adelusola KA, et al. The severity, outcome and challenges of breast cancer in Nigeria. *Breast* 2006;15(3):399-409. doi: 10.1016/j.breast.2005.06.008 [published Online First: 2005/08/09]
26. Ahmed A, Ukwenya Y, Abdullahi A, et al. Management and Outcomes of Male Breast Cancer in Zaria, Nigeria. *Int J Breast Cancer* 2012;2012:6. doi: 10.1155/2012/845143
27. Eze JN, Emeka-Irem EN, Edegbe FO. A Six-Year Study of the Clinical Presentation of Cervical Cancer and the Management Challenges Encountered at a State Teaching Hospital in Southeast Nigeria. *Clinical Medicine Insights: Oncology* 2013(7):151-58. doi: 10.4137/CMO.S12017
28. Alatise OI, Fischer SE, Ayandipo OO, et al. Health-Seeking Behavior and Barriers to Care in Patients With Rectal Bleeding in Nigeria. *J Glob Oncol* 2017;3(6):749-56. doi: https://dx.doi.org/10.1200/JGO.2016.006601
29. Brown BJ, Adeleye AO, Ibeh JN. A Prospective Study on the Causes of Delayed Diagnosis of Childhood Cancer in Ibadan, Nigeria. *Pediatr Hematol Oncol* 2015;32(6):365-73. doi: https://dx.doi.org/10.3109/08880018.2015.1040933
30. Dye TD, Bogale S, Hobden C, et al. Complex care systems in developing countries: breast cancer patient navigation in Ethiopia. *Cancer* 2010;116(3):577-85. doi: https://dx.doi.org/10.1002/cncr.24776
31. Jemebere W. Barriers Associated with Presentation Delay among Breast Cancer Patients at Hawassa University Comprehensive and Specialized Hospital, Southern Ethiopia. *International Journal of Caring Sciences* 2019;12(3):1356-63.
32. Begoihn M, Mathewos A, Aynalem A, et al. Cervical cancer in Ethiopia – predictors of advanced stage and prolonged time to diagnosis. *Infectious Agents and Cancer* 2019;14(1):36. doi: 10.1186/s13027-019-0255-4
33. Aziato L, Clegg-Lamptey JNA. Breast Cancer Diagnosis and Factors Influencing Treatment Decisions in Ghana. *Health Care Women Int* 2015;36(5):543-57. doi: 10.1080/07399332.2014.911299
34. Agbokey F, Kudzawu E, Dzodzomenyo M, et al. Knowledge and Health Seeking Behaviour of Breast Cancer Patients in Ghana. *Int J Breast Cancer* 2019;2019:5239840. doi: 10.1155/2019/5239840

For peer review only

35. Chu KM, Mahlangeni G, Swannet S, et al. AIDS-associated Kaposi's sarcoma is linked to advanced disease and high mortality in a primary care HIV programme in South Africa. *J Int AIDS Soc* 2010;13:23. doi: https://dx.doi.org/10.1186/1758-2652-13-23
36. Antel K, Levetan C, Mohamed Z, et al. The determinants and impact of diagnostic delay in lymphoma in a TB and HIV endemic setting. *BMC Cancer* 2019;19(1):384. doi: 10.1186/s12885-019-5586-4 [published Online First: 2019/04/27]
37. Afungchwi GM, Hesseling PB, Ladas EJ. The role of traditional healers in the diagnosis and management of Burkitt lymphoma in Cameroon: understanding the challenges and moving forward. *BMC Complementary & Alternative Medicine* 2017;17:1-7. doi: 10.1186/s12906-017-1719-y
38. Mlange R, Matovelo D, Rambau P, et al. Patient and disease characteristics associated with late tumour stage at presentation of cervical cancer in northwestern Tanzania. *BMC Womens Health* 2016;16(5):1-6. doi: 10.1186/s12905-016-0285-7
39. Njuguna F, Martijn H, Langat S, et al. Factors influencing time to diagnosis and treatment among pediatric oncology patients in Kenya. *Pediatr Hematol Oncol* 2016;33(3):186-99. doi: https://dx.doi.org/10.3109/08880018.2016.1169566
40. Freeman E, Semeere A, Wenger M, et al. Pitfalls of practicing cancer epidemiology in resource-limited settings: the case of survival and loss to follow-up after a diagnosis of Kaposi's sarcoma in five countries across sub-Saharan Africa. *BMC Cancer* 2016;16:65. doi: https://dx.doi.org/10.1186/s12885-016-2080-0
41. Elliss-Brookes L, McPhail S, Ives A, et al. Routes to diagnosis for cancer - determining the patient journey using multiple routine data sets. *Br J Cancer* 2012;107(8):1220-26. doi: 10.1038/bjc.2012.408 [published Online First: 2012/09/20]
42. Palser TR, Cromwell DA, Hardwick RH, et al. Impact of route to diagnosis on treatment intent and 1-year survival in patients diagnosed with oesophagogastric cancer in England: a prospective cohort study. *BMJ Open* 2013;3(2):e002129. doi: 10.1136/bmjopen-2012-002129
43. Lamri L, Gripiotis E, Ferrario A. Diabetes in Algeria and challenges for health policy: a literature review of prevalence, cost, management and outcomes of diabetes and its complications. *Globalization and Health* 2014;10(1):11. doi: 10.1186/1744-8603-10-11
44. Mahfoud N, Brahamia B. The problems of funding the health system in Algeria. *International Journal of Medicine and*

Pharmaceutical Sciences (IJMPS) 2014;2(2):121-30
45. Hamilton W. Five misconceptions in cancer diagnosis. *Br J Gen Pract* 2009;59(563):441-47.
46. Stefan DC. Cancer Care in Africa: An Overview of Resources. *J Glob Oncol* 2015;1(1):30-36. doi: 10.1200/JGO.2015.000406
47. James PB, Wardle J, Steel A, et al. Traditional, complementary and alternative medicine use in Sub-Saharan Africa: a systematic review. *BMJ Global Health* 2018;3(5):e000895. doi: 10.1136/bmjgh-2018-000895
48. Kingham TP, Alatise OI, Vanderpuye V, et al. Treatment of cancer in sub-Saharan Africa. *Lancet Oncol* 2013;14(4):e158-67. doi: 10.1016/s1470-2045(12)70472-2 [published Online First: 2013/04/09]
49. Mash R, Howe A, Olayemi O, et al. Reflections on family medicine and primary healthcare in sub-Saharan Africa. *BMJ Global Health* 2018;3(Suppl 3):e000662. doi: 10.1136/bmjgh-2017-000662
50. A OA, Taiwo O, S AO, et al. Breast cancer awareness, attitude and screening practices in Nigeria: A systematic review. *Clinical Reviews and Opinions* 2016;7(2):11-25. doi: 10.5897/cro16.0101
51. McKenzie F, Zietsman A, Galukande M, et al. Breast cancer awareness in the sub-Saharan African ABC-DO cohort: African Breast Cancer—Disparities in Outcomes study. *Cancer Causes Control* 2018;29(8):721-30. doi: 10.1007/s10552-018-1047-7

For peer review only - http://bmjopen.bmj.com/site/about/guidelines.xhtml

52. Economist Intelligence Unit. The future of healthcare in Africa. 2012 [Available from: https://perspectives.eiu.com/sites/default/files/EIU-Janssen_HealthcareAfrica_Report_Web.pdf accessed 27/11 2019.
53. Campbell J DG, Buchan J, Pozo-Martin F, Guerra Arias M, Leone C, Siyam A, Cometto G.,. The future of healthcare in Africa,. Forum Report, Third Global Forum on Human Resources for Health, Recife, Brazil Geneva, Global Health Workforce Alliance and World Health Organization, 2013.
54. Keene MR, Heslop IM, Sabesan SS, et al. Complementary and alternative medicine use in cancer: A systematic review. *Complementary Therapies in Clinical Practice* 2019;35:33-47. doi: https://doi.org/10.1016/j.ctcp.2019.01.004
55. Bishop FL, Prescott P, Chan YK, et al. Prevalence of complementary medicine use in pediatric cancer: a systematic review. *Pediatrics* 2010;125(4):768-76. doi: 10.1542/peds.2009-1775 [published Online First: 2010/03/24]
56. Shirwaikar A, Govindarajan R, Rawat AKS. Integrating Complementary and Alternative Medicine with Primary Health Care. *Evidence-Based Complementary and Alternative Medicine* 2013;2013:948308. doi: 10.1155/2013/948308
57. Johnson SB, Park HS, Gross CP, et al. Use of Alternative Medicine for Cancer and Its Impact on Survival. *JNCI: Journal of the National Cancer Institute* 2017;110(1):121-24. doi: 10.1093/jnci/djx145

For peer review only

For peer review only - http://bmjopen.bmj.com/site/about/guidelines.xhtml

Figure 1: Flow chart of study selection process

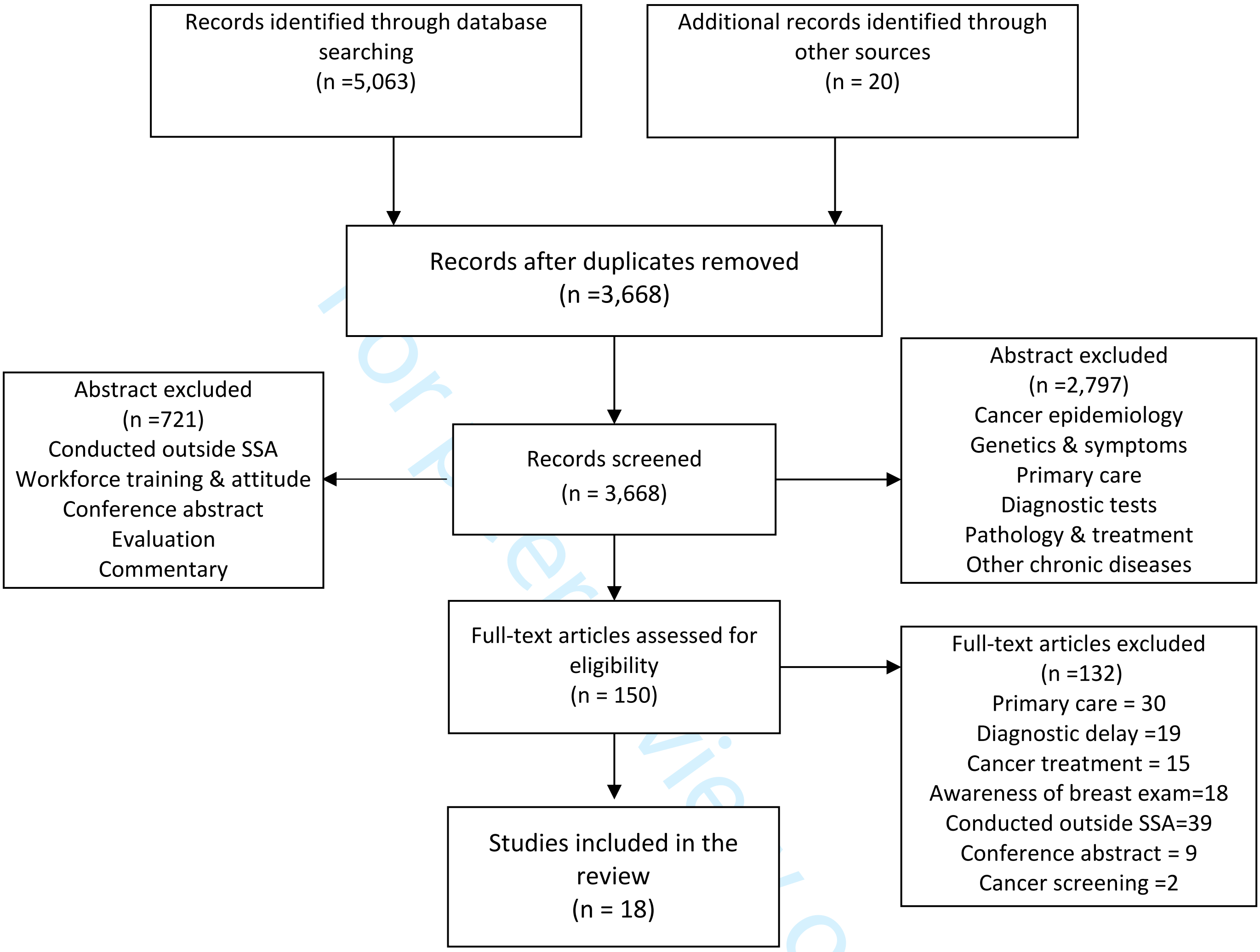

For peer review only

PRISMA 2009 Checklist

Section/topic	#	Checklist item	Reported on page #
**TITLE**			
Title	1	Identify the report as a systematic review, meta-analysis, or both.	1
**ABSTRACT**			
Structured summary	2	Provide a structured summary including, as applicable: background; objectives; data sources; study eligibility criteria, participants, and interventions; study appraisal and synthesis methods; results; limitations; conclusions and implications of key findings; systematic review registration number.	2
**INTRODUCTION**			
Rationale	3	Describe the rationale for the review in the context of what is already known.	4
Objectives	4	Provide an explicit statement of questions being addressed with reference to participants, interventions, comparisons, outcomes, and study design (PICOS).	5
**METHODS**			
Protocol and registration	5	Indicate if a review protocol exists, if and where it can be accessed (e.g., Web address), and, if available, provide registration information including registration number.	N/A
Eligibility criteria	6	Specify study characteristics (e.g., PICOS, length of follow-up) and report characteristics (e.g., years considered, language, publication status) used as criteria for eligibility, giving rationale.	5
Information sources	7	Describe all information sources (e.g., databases with dates of coverage, contact with study authors to identify additional studies) in the search and date last searched.	5
Search	8	Present full electronic search strategy for at least one database, including any limits used, such that it could be repeated.	Additional file 2
Study selection	9	State the process for selecting studies (i.e., screening, eligibility, included in systematic review, and, if applicable, included in the meta-analysis).	5-7
Data collection process	10	Describe method of data extraction from reports (e.g., piloted forms, independently, in duplicate) and any processes for obtaining and confirming data from investigators.	6
Data items	11	List and define all variables for which data were sought (e.g., PICOS, funding sources) and any assumptions and simplifications made.	6
Risk of bias in individual studies	12	Describe methods used for assessing risk of bias of individual studies (including specification of whether this was done at the study or outcome level), and how this information is to be used in any data synthesis.	6-7
Summary measures	13	State the principal summary measures (e.g., risk ratio, difference in means).	N/A
Synthesis of results	14	Describe the methods of handling data and combining results of studies, if done, including measures of consistency (e.g., I^2) for each meta-analysis.	6

For peer review only - http://bmjopen.bmj.com/site/about/guidelines.xhtml

PRISMA 2009 Checklist

Section/topic	#	Checklist item	Reported on page #
Risk of bias across studies	15	Specify any assessment of risk of bias that may affect the cumulative evidence (e.g., publication bias, selective reporting within studies).	6 & 8
Additional analyses	16	Describe methods of additional analyses (e.g., sensitivity or subgroup analyses, meta-regression), if done, indicating which were pre-specified.	N/A
**RESULTS**			
Study selection	17	Give numbers of studies screened, assessed for eligibility, and included in the review, with reasons for exclusions at each stage, ideally with a flow diagram.	7 - 8 and Figure 1
Study characteristics	18	For each study, present characteristics for which data were extracted (e.g., study size, PICOS, follow-up period) and provide the citations.	7-9 and 16-18
Risk of bias within studies	19	Present data on risk of bias of each study and, if available, any outcome level assessment (see item 12).	8 & 19
Results of individual studies	20	For all outcomes considered (benefits or harms), present, for each study: (a) simple summary data for each intervention group (b) effect estimates and confidence intervals, ideally with a forest plot.	7-9 & 16-18
Synthesis of results	21	Present results of each meta-analysis done, including confidence intervals and measures of consistency.	N/A
Risk of bias across studies	22	Present results of any assessment of risk of bias across studies (see Item 15).	8 & 19
Additional analysis	23	Give results of additional analyses, if done (e.g., sensitivity or subgroup analyses, meta-regression [see Item 16]).	N/A
**DISCUSSION**			
Summary of evidence	24	Summarize the main findings including the strength of evidence for each main outcome; consider their relevance to key groups (e.g., healthcare providers, users, and policy makers).	10
Limitations	25	Discuss limitations at study and outcome level (e.g., risk of bias), and at review-level (e.g., incomplete retrieval of identified research, reporting bias).	10
Conclusions	26	Provide a general interpretation of the results in the context of other evidence, and implications for future research.	13
**FUNDING**			
Funding	27	Describe sources of funding for the systematic review and other support (e.g., supply of data); role of funders for the systematic review.	20

From: Moher D, Liberati A, Tetzlaff J, Altman DG, The PRISMA Group (2009). Preferred Reporting Items for Systematic Reviews and Meta-Analyses: The PRISMA Statement. PLoS Med 6(7): e1000097. doi:10.1371/journal.pmed1000097

For more information, visit: **www.prisma-statement.org**.

For peer review only - http://bmjopen.bmj.com/site/about/guidelines.xhtml

Additional file 2: Ovid MEDLINE(R), PsycINFO, Embase, Global Health

Set	Search Statement
1.	Angola*.ti,ab.
2.	Gabon*.ti,ab.
3.	Nigeria*.ti,ab.
4.	Benin*.ti,ab.
5.	Gambia*.ti,ab.
6.	Rwanda*.ti,ab.
7.	Botswana*.ti,ab.
8.	Ghana*.ti,ab.
9.	Sao Tome*.ti,ab.
10.	Sao Tome.mp. and Principe*.ti,ab. [mp=title, abstract, heading word, drug trade name, original title, device manufacturer, drug manufacturer, device trade name, keyword, floating subheading word, candidate term word]
11.	Burkina Faso*.ti,ab.
12.	Guinea-Bissau*.ti,ab.
13.	Seychelles*.ti,ab.
14.	Guinea*.ti,ab.
15.	Senegal*.ti,ab.
16.	Burundi*.ti,ab.
17.	Cabo Verde*.ti,ab.
18.	Kenya*.ti,ab.
19.	Sierra Leone*.ti,ab.
20.	Cameroon*.ti,ab.
21.	Lesotho*.ti,ab.
22.	Somalia*.ti,ab.
23.	Central African Republic*.ti,ab.
24.	Liberia*.ti,ab.
25.	South Africa*.ti,ab.
26.	Chad*.ti,ab.
27.	Madagascar*.ti,ab.
28.	South Sudan*.ti,ab.
29.	Comoros*.ti,ab.
30.	Malawi*.ti,ab.
31.	Sudan*.ti,ab.
32.	Congo*.ti,ab.
33.	Mali*.ti,ab.
34.	Swaziland*.ti,ab.
35.	Mauritania*.ti,ab.
36.	Tanzania*.ti,ab.
37.	Cote d'Ivoire*.ti,ab.
38.	Mauritius*.ti,ab.
39.	togo*.ti,ab.
40.	Equatorial Guinea*.ti,ab.
41.	Mozambique*.ti,ab.
42.	Uganda*.ti,ab.
43.	Eritrea*.ti,ab.
44.	Namibia*.ti,ab.
45.	Zambia*.ti,ab.
46.	Ethiopia*.ti,ab.
47.	Niger*.ti,ab.
48.	Zimbabwe*.ti,ab.
49.	Africa*.ti,ab.

For peer review only

50.	Sub-Sahara*.ti,ab.
51.	1 or 2 or 3 or 4 or 5 or 6 or 7 or 8 or 9 or 10 or 11 or 12 or 13 or 14 or 15 or 16 or 17 or 18 or 19 or 20 or 21 or 22 or 23 or 24 or 25 or 26 or 27 or 28 or 29 or 30 or 31 or 32 or 33 or 34 or 35 or 36 or 37 or 38 or 39 or 40 or 41 or 42 or 43 or 44 or 45 or 46 or 47 or 48 or 49 or 50
52.	cancer*.ti,ab.
53.	exp Neoplasms/
54.	(adenocarcinoma or astrocytoma or glioma or Hodgkin$ or leuk?emia or lymphoma or medulloblastoma or melanoma or mesothelioma or myeloma or neuroblastoma or nonmelanoma or osteosarcoma or retinoblastoma or sarcoma or seminoma or teratoma).ti,ab.
55.	52 or 53 or 54
56.	(pathway adj5 diagnos$).ti,ab.
57.	(pathway adj5 detect$).ti,ab.
58.	(route adj5 diagnos$).ti,ab.
59.	(route adj5 detect$).ti,ab.
60.	diagnos$.ti,ab.
61.	detect$.ti,ab.
62.	consult$.ti,ab.
63.	(help adj5 seek$).ti,ab.
64.	present$.ti,ab.
65.	(route adj5 consult$).ti,ab.
66.	(route adj5 present$).ti,ab.
67.	(pathway adj5 consult$).ti,ab.
68.	(pathway adj5 present$).ti,ab.
69.	56 or 57 or 58 or 59 or 60 or 61 or 62 or 63 or 64 or 65 or 66 or 67 or 68
70.	primary care.ti,ab.
71.	family doctor.ti,ab.
72.	physician.ti,ab.
73.	(health adj5 practitioner).ti,ab.
74.	General Practitioners/ or Family Practice/ or Primary Health Care/
75.	70 or 71 or 72 or 73 or 74
76.	51 and 55 and 69 and 75

For peer review only

BMJ Open

Routes to diagnosis of symptomatic cancer in Sub-Saharan Africa: a systematic review

Journal:	*BMJ Open*
Manuscript ID	bmjopen-2020-038605.R2
Article Type:	Original research
Date Submitted by the Author:	02-Oct-2020
Complete List of Authors:	Martins, Tanimola; University of Exeter, College of Medicine & Health<br>Merriel, Samuel; University of Exeter, College of Medicine & Health<br>Hamilton, Willie; University of Exeter, Primary Care
**Primary Subject Heading**:	General practice / Family practice
Secondary Subject Heading:	Epidemiology, Public health, Oncology
Keywords:	PRIMARY CARE, PUBLIC HEALTH, Epidemiology < ONCOLOGY, International health services < HEALTH SERVICES ADMINISTRATION & MANAGEMENT

SCHOLARONE™
Manuscripts

I, the Submitting Author has the right to grant and does grant on behalf of all authors of the Work (as defined in the below author licence), an exclusive licence and/or a non-exclusive licence for contributions from authors who are: i) UK Crown employees; ii) where BMJ has agreed a CC-BY licence shall apply, and/or iii) in accordance with the terms applicable for US Federal Government officers or employees acting as part of their official duties; on a worldwide, perpetual, irrevocable, royalty-free basis to BMJ Publishing Group Ltd ("BMJ") its licensees and where the relevant Journal is co-owned by BMJ to the co-owners of the Journal, to publish the Work in this journal and any other BMJ products and to exploit all rights, as set out in our licence.

The Submitting Author accepts and understands that any supply made under these terms is made by BMJ to the Submitting Author unless you are acting as an employee on behalf of your employer or a postgraduate student of an affiliated institution which is paying any applicable article publishing charge ("APC") for Open Access articles. Where the Submitting Author wishes to make the Work available on an Open Access basis (and intends to pay the relevant APC), the terms of reuse of such Open Access shall be governed by a Creative Commons licence – details of these licences and which Creative Commons licence will apply to this Work are set out in our licence referred to above.

Other than as permitted in any relevant BMJ Author's Self Archiving Policies, I confirm this Work has not been accepted for publication elsewhere, is not being considered for publication elsewhere and does not duplicate material already published. I confirm all authors consent to publication of this Work and authorise the granting of this licence.

For peer review only

Routes to diagnosis of symptomatic cancer in Sub-Saharan Africa: a systematic review

Tanimola Martins *, Postdoctoral Research Fellow [1]

Samuel W D Merriel, Clinical Senior Research Fellow [1]

William Hamilton, Professor of Primary care Diagnostics [1]

[1] University of Exeter, College of Medicine & Health, College House St Luke's Campus, Magdalen Road, Exeter, EX1 2LU

*Correspondence author's email: tom207@exeter.ac.uk

For peer review only

Abstract

Background: Most cancers in Sub-Saharan Africa (SSA) are diagnosed at advanced stages, with limited treatment options and poor outcomes. Part of this may be linked to various events occurring in patients' journey to diagnosis. Using the Model of Pathways to Treatment, we examined the evidence regarding the routes to cancer diagnosis in SSA.

Design and settings: A systematic review of available literature was performed

Methods: The PRISMA guidelines were followed. Between September 30th and November 30th 2019, seven electronic databases were searched using terms relating to SSA countries, cancer, and routes to diagnosis; comprising the population, exposure, and outcomes, respectively. Citation lists of included studies were manually searched to identify relevant studies. Furthermore, ProQuest Dissertations & Theses Global was searched to identify appropriate grey literature on the subject.

Results: 18 of 5,083 references identified met the inclusion criteria: eight focused on breast cancer, three focused on cervical cancer, two each focused on lymphoma, Kaposi's sarcoma, and childhood cancers, and one on colorectal cancer. With the exception of Kaposi's sarcoma, definitive diagnoses were made in tertiary healthcare centres, including teaching and regional hospitals. The majority of participants initially consulted within primary care, although a considerable proportion first used alternative medicine before seeking conventional medical help. The quality of included studies was a major concern, but their findings provide important insight into the pathways to cancer diagnosis in the region.

Conclusion: The proportion of patients who initially use alternative medicine in their cancer journey may explain a fraction of advanced-stage diagnosis and poor survival of cancer in SSA. However, further research would be necessary to fully understand the exact role (or activities) of primary care and alternative care providers in patient cancer journeys.

For peer review only - http://bmjopen.bmj.com/site/about/guidelines.xhtml

Strengths and limitations of this study

- This is the first systematic review of the evidence relating to the routes to diagnosis of cancer in Sub-Saharan Africa
- The search strategies, assessment of quality, and narrative synthesis followed good practice
- Selected studies used small sample sizes and systematically introduced biases in the selection of participants and data collection.
- However, their findings provide unique insights into patients' journey to cancer diagnosis in Sub-Saharan Africa.

For peer review only

For peer review only - http://bmjopen.bmj.com/site/about/guidelines.xhtml

Background

Sub-Saharan Africa (SSA) is overburdened with communicable diseases, whilst the incidence and mortality from non-communicable diseases such as cancer are rising across the region.[1] The increase in cancer incidence is associated with poor control of cancer-related infections and unhealthy lifestyle choices, which may be addressed, in part, by implementing effective public health interventions.[2-6] Mortality from cancer is strongly associated with stage at diagnosis; early-stage cancers enable treatment with curative intent and better prognoses than late-stage diseases.[7-9] Most cancers in SSA are diagnosed at advanced stages, due to late presentation of symptoms, weak referral mechanisms, and limited diagnostic capacity.[8-10] Early-stage cancers and precancerous lesions are detectable by screening asymptomatic patients, but this is limited to few sites and very rarely used in SSA. Therefore, interventions aimed at promoting early symptomatic presentation and expedited diagnosis are likely to yield better cancer outcomes in the region. However, such interventions must be rooted in empirical evidence to ensure effectiveness and maximise local resources use.

The Model of Pathways to Treatment offers a useful framework to examine the routes to diagnosis of symptomatic cancer.[11] It describes five possible events in the pathways to treatment: detection of bodily changes, perceived reasons to seek medical help, first consultation with a healthcare provider, diagnosis, and start of treatment.[11] Numerous studies have explored these events in cancer, but only a few have specifically investigated patients' initial contact with healthcare providers in SSA.[12] Using this framework, we investigated patients' routes to cancer diagnosis in SSA, focusing on the initial point of consultation and eventual diagnosis. Identifying and categorising the routes to diagnosis may explain advanced-stage cancers and provide the basis for early diagnosis interventions in the region.

Methods

A systematic narrative review was performed. The conduct and reporting of the review was based on the Preferred Reporting Items for Systematic Reviews and Meta-Analysis (PRISMA) framework [see Additional file 1].[13]

For peer review only - http://bmjopen.bmj.com/site/about/guidelines.xhtml

Search Strategy

Between September 30th and November 30th 2019, a systematic search of the following electronic databases was performed: Ovid MEDLINE(R) ALL (1946 to September 30, 2019), Embase (1974 to 2019 September 30), Web of Science (1915 (1) - 2019 (69)), PsycINFO (1806 to September Week 2 2019), CINAHL Complete, Global Health (1973 to 2019 Week 36) and African Journals Online (AJOL). The search strategy included terms, their synonyms and MeSH terms relating to SSA countries, cancer, and routes to diagnosis; comprising the population, exposure and outcomes, respectively (Table 1). Additional file 2 shows the search strategy in MEDLINE, PsycINFO, Embase, and Global Health. Citation lists of included studies were manually searched to identify relevant studies. Furthermore, ProQuest Dissertations & Theses Global was searched to identify appropriate grey literature on the subject.

Eligibility criteria

Included studies investigated cancer diagnosis, described the routes or patient's pathway to diagnosis (including the settings of initial consultation and definitive diagnosis), and were conducted in one or more of the 48 SSA countries. The list of SSA countries matches those featured on the World Bank data catalogue used to describe health and socio-economic indices in the region.[14] Excluded studies were non-English studies, focused on populations outside the region of SSA, investigated diseases other than cancer, cancer treatment, outcomes, and attitudes toward cancer diagnoses. All study designs (qualitative and quantitative) were eligible for inclusion.

Study Selection

This involved a two-stage screening process. Firstly, title, abstract and full articles of potentially eligible studies were sequentially screened by an experienced researcher (TM) against the inclusion and exclusion criteria. Consequently, studies that appeared to meet the inclusion criteria or where a decision could not be made based on the title and/or abstract were selected for full-text review to identify those for the final analysis.

For peer review only - http://bmjopen.bmj.com/site/about/guidelines.xhtml

Data Extraction and synthesis

One reviewer (TM) extracted data from all included studies. Extracted data were added to a data extraction spreadsheet, which was initially piloted with seven studies. Data extraction included study characteristics: country of study, design, participants' characteristics, cancer type, health care settings for initial consultation, and eventual diagnosis. Quantitative synthesis was not possible because our final selection differed in terms of cancer sites and outcome measures. For instance, some studies described patients initially presenting to "health care practitioner", a term that may be used to describe primary care physicians or doctors in secondary care. Therefore, we performed a narrative synthesis, using the framework of Rodgers and colleagues.[15] Participants' characteristics and study main findings are illustrated in tables and figures.

Quality assessment

Three reviewers (TM, WH and SM) assessed the methodological quality of eligible studies using the Newcastle-Ottawa Quality Assessment Scale (NOS) for cohort, NOS adapted for cross-sectional studies, [16 17] and the Joanna Briggs Institute (JBI) Critical Appraisal Checklist for Qualitative Research.[18]TM and SM independently selected the appropriate checklist based on study design. The cohort and cross-sectional studies were awarded stars and rated "good", "satisfactory" or "poor quality", depending on the extent to which they meet the NOS checklists criteria on the three main domains: selection, comparability, outcomes alongside associated statistics. Good-quality studies were awarded four stars in the selection domain, and two stars in each of the comparability and outcome domains. Studies rated satisfactory were awarded two stars in the selection domain, one star in comparability domain, and up to 3 stars in the outcome domain. Poor-quality studies were awarded 0 stars in the comparability domain, and one star in the selection or outcome domains. The JBI checklist is not a scoring system but a useful tool for evaluating the risk of bias in the design and conduct of qualitative studies. The checklist consists of 10 criteria with four possible responses: "yes,"

For peer review only - http://bmjopen.bmj.com/site/about/guidelines.xhtml

"no," "unclear," and "inapplicable." Each qualitative study was evaluated against the checklist criteria. Discrepancies between the reviewers were resolved by consensus, although no study was excluded based on quality.

Patient and Public Involvement

There was no formal patient and public involvement in this review.

Result

Study characteristics

The search identified 5,083 articles. After screening title and abstract, and removing duplicates, 4,933 irrelevant articles were excluded: 150 full text articles were assessed with 18 meeting the inclusion criteria. A PRISMA flowchart showing the reasons for abstract and full article exclusions is shown in Figure 1. The 18 studies recruited a total of 4,871 participants from nine SSA countries, 70% of which were females with the average age ranging from 4 to 59 years. The characteristics of included studies are illustrated in Table 2, with the results of quality assessment in Table 3. Seven of the studies were conducted in Nigeria,[19-25] three in Ethiopia,[26-28] two each in Ghana,[29 30] and South Africa,[31 32] and one each in Cameroon,[33] Tanzania,[34] and Kenya.[35]The final study involved five countries (Kenya, Uganda, Malawi, Cameroon and Nigeria).[36]

All 18 studies were observational with seven cross-sectional surveys, seven cohorts (using medical records), three qualitative (face-to-face interviews), and a mixed-methods study (using both qualitative and quantitative data). Eight studies examined breast cancer,[19-22 26 27 29 30] three focused on cervical cancer,[23 28 34]two each focused on lymphoma,[32 33] Kaposi's sarcoma,[31 36] and childhood cancers,[25 35] and one on colorectal cancer.[24] None of the 18 studies specifically investigated the routes to cancer diagnosis, although 15 studies reported the settings of initial consultation after symptoms onset. The remaining three studies recruited participants from primary care-based HIV clinics to investigate Kaposi's sarcoma and lymphoma diagnoses.[31 32 36] These studies were included in our final

For peer review only - http://bmjopen.bmj.com/site/about/guidelines.xhtml

selection given that both cancer types are significantly more common in HIV patients and that HIV patients are mostly seen at such settings.

Assessment of study quality

Overall, none of the qualitative studies fulfilled the JBI checklist criteria and none of the quantitative studies could be classified as ”good quality” due to the limitations in their methodology (Table 3). The main limitations of these studies pertained to their small sample sizes, biases in participant recruitment, and data collection strategies. The sample sizes in most of the cohort and cross-sectional studies were rather small to be representative of the target population. Four-fifth of included studies recruited participants from tertiary healthcare centres, thereby introducing selection bias by systematically excluding patients diagnosed or treated elsewhere. In some studies, surveys and face-to-face interviews were performed by nurses or physician-researchers from the hospitals where participants were receiving treatment, thus drawing possibly desirable responses. Additionally, statistical analyses were largely descriptive, with most studies presenting percentages only. Despite these limits, however, the studies provided some important findings relevant to the aim of our review, thereby warranting their inclusion in the synthesis.

Routes to cancer diagnosis

Across the eight studies on breast cancer, providers in tertiary healthcare centres made the definitive diagnoses in all cases (Table 2).[19-22 26 27 29 30] After noticing symptoms, participants initially consulted the physicians (in primary or secondary care), used alternative medicine (including traditional healers, herbalists, and prayer centres), or presented directly to the hospital. The proportion of patients using each of these routes to diagnosis differed slightly between studies but very similar across all the eight studies.[19-22 26 27 29 30] On average, around a third of the participants - across the studies - initially presented symptoms to each of the physician, alternative medicine practitioners, or directly to the hospital.

For peer review only - http://bmjopen.bmj.com/site/about/guidelines.xhtml

For peer review only

In two of the three studies focused on cervical cancer, participants presented with symptoms directly to tertiary health centres where cervical cancer diagnoses were confirmed. (Table 2).[23 28] Conversely, 47% of participants in the third study initially presented symptoms to traditional healthcare practitioners before returning to the tertiary health centres for diagnosis and start of treatment.[34]

In a survey of 82 patients with rectal bleeding and colorectal cancer, Alatise *et al.* found that only 39% of the participants had consulted a physician, with 38% of participants opting to use herbs before going to the doctors (Table 2).[24]

Of 6,292 HIV infected patients enrolled at an HIV clinic, Chu *et al.* found 3% diagnosed with Kaposi's sarcoma within seven years of routine HIV care.[31] Similarly, healthcare providers from 33 HIV clinics across five African countries diagnosed 1,328 HIV patients with Kaposi's sarcoma during four years of routine HIV care.[36] In both studies, providers at the HIV clinics detected Kaposi's sarcomas during routine examination for opportunistic infections.

Two studies surveyed parents and carers of children with childhood cancers to determine causes of diagnostic delay. In one study, 59% of parents initially sought alternative medicine for their children, although about 60% later consulted in primary care, 38% in secondary care, and 2% presented directly to tertiary care.[35] In contrast, 69% of parents in the second study initially sought conventional medical help, but 24% either self-medicated, used herbalist services or presented to a church.[25]

In a survey of parents and carers of children with Burkitt lymphoma, Afungchwi and colleagues showed that 55% had used traditional healers before hospital admission, with 42% using this service before reporting to primary care.[33] In contrast, all 163 patients diagnosed with Hodgkin and Non-Hodgkin lymphoma in Antel *et al's* study were referred to the specialist by healthcare practitioners.[32]

Discussion

The route to diagnosis is a strong predictor of cancer outcomes.[37 38] In this review, we examined the evidence relating to cancer diagnosis in SSA. Across all selected studies, definitive diagnoses of cancer

For peer review only - http://bmjopen.bmj.com/site/about/guidelines.xhtml

were made by specialists in large tertiary healthcare centres, except for Kaposi's sarcomas, which were diagnosed at various primary care-based specialist clinics. However, participants' journeys to the specialist clinics are often indirect, with a considerable proportion initially using alternative medicine before consulting conventional medical services.

Strength and limitations

To our knowledge, this is the first systematic review of the evidence regarding the routes to cancer diagnosis in SSA. Our rigorous search strategy and explicit inclusion/exclusion criteria, quality assessment of included studies, and narrative synthesis followed good practice. Our search identified only a modest number of studies, a third of which were conducted in Nigeria, the most populous country with the largest economy in the region. We omitted non-English studies as these may include studies published in French, Portuguese, and other African languages. While the decision to omit these studies may have reduced the number of selected studies slightly, we have no reason to believe that such omission had any impact on our findings.

About half of our final selection focused on breast cancer, reducing the scope of the review. The studies also had small sample sizes, which limits the interpretation and generalisability of our findings. Additionally, the majority recruited participants and gathered data (using researcher-administered questionnaires) from the hospital facilities where patients were being treated for their cancers, typically in the tertiary healthcare centres. This is not surprising, given the weak primary care and limited cancer registries in SSA, thus limiting the quality and quantity of data available for research. However, recruiting participants from tertiary health centres systematically exclude patients treated in private hospitals and those whose cancers may never be found due to affordability or comorbidity. Furthermore, gathering data from the hospital using physician-administered questionnaires may generate more socially desirable responses. In this case, it is likely that participants under-report their use of alternative medicine and self-medication to look good in the eyes of their providers who may be part of the research team.

For peer review only - http://bmjopen.bmj.com/site/about/guidelines.xhtml

Finally, publication bias is possible as some studies on the subject may have failed to be published in reputable peer-reviewed journals, and so would have been omitted from the databases searched for this review.

Interpretation of findings

The pathways to diagnosis of symptomatic cancer involves a series of events, beginning with the patient noticing a bodily change and deciding to seek medical help.[11] Definitive diagnosis requires biopsy of affected tissue by specialists in secondary or tertiary healthcare settings. In developed countries like the UK and Denmark, most cancer patients initially present with symptoms to primary care, with a smaller proportion presenting to secondary care as emergencies.[37] Primary care physicians in these countries play a key role in selecting those whose symptoms warrant specialist investigations using preliminary test results and clinical guidelines.[39] Healthcare services in many SSA countries is pluralistic, comprising a three-tier system: primary care (including dispensaries, health centres and private clinics); secondary care (including private, mission and district hospitals); and tertiary healthcare.[26] The tertiary healthcare centres are referral centres with various sub-specialities, and are the main setting for definitive diagnosis of cancer.[26 28 30 40] However, the role of primary care in SSA is not always well-defined, with several unorthodox providers including traditional healers and faith clinics, offering similar services, albeit unqualified to diagnose cancer or to refer patients for specialist investigations. [9 41-43] Patients in these countries may present with symptoms directly to tertiary healthcare centres, regardless of the nature or duration of symptoms. They may also be referred by physicians in primary or secondary care, but often with no standardised referral pathways or mechanism to ensure continuity of care. [9 41-43] This problem is further compounded by frequent long distances to healthcare centres and out-of-pocket payments, particularly for patients in rural and socioeconomically deprived areas – who may resort to alternative medicine instead.

Indeed, a considerable proportion of participants in this review initially used alternative medicine before consulting in primary care, with some also presenting directly to the hospital. Only a third of

For peer review only - http://bmjopen.bmj.com/site/about/guidelines.xhtml

women with breast cancer initially reported symptoms to primary care, despite widespread awareness campaign with relatively easy to spot symptoms.[44 45] 53% of patients with cervical cancer symptoms, 39% of those with rectal bleeding, and around two-third of childhood cancers initially sought help in primary care. Access to conventional health care is restricted in most SSA countries due to limited availability and affordability.[9 42 46 47] In their respective cancer journey, patients in this region may start with or revert to alternative medicine, which is considered cheaper and more natural, with some practitioners offering complete cure of cancer rather than possible remission offered by conventional medicine.[26 41] The use of alternative medicine is widespread in SSA, although evidence suggests that the practitioners can misdiagnose cancer, resulting in advanced-stage diagnosis and reduced chances of survival.[33 48]

The findings of this review may have been influenced by the level of bias in included studies: in which case, our report on the proportion using various route to diagnosis will be inaccurate. If at all, we may have overestimated the proportion of patients consulting in primary care or underestimated those using alternative medicine before diagnosis, given the lack of public awareness of cancer and weakness of healthcare systems in the region, with significant underdiagnoses.

Conclusion

Recent data from SSA suggests a rapid increase in the risk and deaths from major cancer types. In a region where infectious diseases persist, with limited healthcare budgets and shortages of specialists, urgent solutions are required to minimise the burden of cancer on its rapidly growing and ageing population. The majority of participants in our selected studies initially presented symptoms to primary care, though the proportion first using alternative medicine is considerable. This latter group of patients constitutes a major source of concern, bearing in mind that alternative medicine practitioners in SSA are likely to be unequipped to spot cancer or make a specialist referral when necessary.

For peer review only - http://bmjopen.bmj.com/site/about/guidelines.xhtml

However, there is a need for further research to fully understand patients' pathways to cancer diagnosis in SSA. For instance, our review found that the majority of patients initially presented in primary care, but we are uncertain the exact roles this played in their journey to diagnosis. As such, a comprehensive research programme to examine the role of primary care and alternative care in cancer diagnosis is recommended as this may contribute to the development of possible diagnostic guidelines.

For peer review only

For peer review only - http://bmjopen.bmj.com/site/about/guidelines.xhtml

Table 1: Search terms

Population	Exposure	Outcome
**Terms relating Sub-Saharan Countries:**<br>Angola, Gabon, Nigeria, Benin, Gambia, The Rwanda, Botswana, Ghana, São Tomé and Principe, Burkina Faso, Guinea, Senegal, Burundi, Guinea-Bissau, Seychelles, Cabo Verde, Kenya, Sierra Leone, Cameroon, Lesotho, Somalia, Central African Republic, Liberia, South Africa, Chad<br>Madagascar, South Sudan, Comoros, Malawi, Sudan, Congo, Dem. Rep., Mali, Swaziland, Congo Rep., Mauritania, Tanzania, Côte d'Ivoire, Mauritius, Togo, Equatorial Guinea, Mozambique, Uganda, Eritrea, Namibia, Zambia, Ethiopia, Niger, Zimbabwe	**Terms relating to Cancer:**<br>Cancer, Neoplasm, Malignant Neoplasm, tumour, Malignant tumour, Astrocytoma, Adenocarcinoma, Glioma, Mesothelioma, Medulloblastoma, Myeloma, Melanoma, Neuroblastoma, Sarcoma, Nonmelanoma, Osteosarcoma, Teratoma, Seminoma, Hodgkin, Leukaemia, Lymphoma, Retinoblastoma	**Terms relating to the routes to Cancer Diagnosis:**<br>Pathway to diagnosis<br>Pathway to detect*<br>Routes to diagnos*<br>Routes to detect*<br>Diagnos*<br>Detect*<br>Consult*<br>Help-seek*<br>Present*<br>Route to consult*<br>Routes to present*<br>Pathway to consult*<br>Pathway to present*<br>Primary care<br>Family doctor<br>Physician<br>Health care practitioner<br>General Practitioners<br>Family Practice<br>Primary Health Care

For peer review only

For peer review only - http://bmjopen.bmj.com/site/about/guidelines.xhtml

Table 2: Study characteristics

Author	Country	Type/site	Title	Method	Outcome measure	Sample characteristics	Relevant findings
Dye *et al.* 2010[26]	**Ethiopia**	**Breast**	Complex care systems in developing countries: breast cancer patient navigation in Ethiopia	A mixed-methods study using semi-structure interview to investigate participant's navigation through the health system before arriving at the tertiary health centre for treatment.	Patient navigation through the health care system that culminated in the treatment of cancer	**Participants:** 55 patients with breast cancer plus 14 carers.<br>**Mean age:** 45.5 years<br>**Sex:** 98% females	Initial presentation of symptoms was:<br>53.7% to primary care<br>16.4% to traditional healers<br>16.4% to local/regional hospital<br>9% to private hospital<br>4.5% directly to the tertiary referral centre.<br>Definitive diagnoses were made at the tertiary health centre.
Jemebere 2019[27]	**Ethiopia**	**Breast**	Barriers Associated with Presentation Delay among Breast Cancer Patients at Hawassa University Comprehensive and Specialized Hospital, Southern Ethiopia	Cross-sectional survey of women diagnosed with breast cancer at a specialised hospital	Route to diagnosis	**Participants:** 106 women<br>**Age range:** 15 - 65years.<br>**Occupation:** Farmers (3%), Labourer (9%), Merchant (16%), Professional 29%) and Housewife (43%).<br>**Education:** None (28%)<br>Elementary (29%), High (24%) and College≥ (19%).<br>Family history of breast cancer (13%)	64% delayed presenting to the hospital due to initial use of alternative medicine including: herbal remedy, traditional healers and prayers.<br><br>Definitive diagnoses were made at the teaching hospital.
Ezeome *et al.* 2010[19]	**Nigeria**	**Breast**	Delays in presentation and treatment of breast cancer in Enugu, Nigeria	Cross-sectional survey of breast cancer patients at an oncology specialist unit (in a teaching hospital).	Patients first point of symptom(s) presentation<br><br>Patients first point of conventional medical treatment	**Participants:** 164 patients (162 female & 2 males) **Median (range) age:** 45 (21-77) years<br>**Socioeconomic status:**<br>Low (59%), Middle (40%), High (1%)<br>**Education:** None (15%), Primary (24%), Secondary (29%), Degree (30%)<br>**Religion:** 96% were Christians	13.1% first used traditional healers<br>4.4% first presented to a prayer house.<br>82.3% initially presented to health care facilities.<br>First point of contact with medical facilities:<br>50% within primary (GP)<br>25% to consultant surgeon<br>10.1% to patent medicine dealer<br>7.5% to a gynaecologist<br>5% to a nurse or allied health professional
Pruitt *et al.* 2015[20]	**Nigeria**	**Breast**	Social barriers to diagnosis and treatment of breast cancer in patients presenting at a teaching hospital in Ibadan, Nigeria	A qualitative study which used semi-structured interview of patients with breast cancer in a teaching hospital.	Help-seeking behaviour after noticing symptoms.	**Participants:** 31 women with breast cancer.<br>**Median (range) age:** 51 (28-80≥)years<br>**Education:** None (n=7), Primary/Secondary (n= 15)<br>Tertiary (n=9).<br>**Religion:** Christians (83%) and Muslim (17%)	Most women rapidly sought orthodox medical care once they noticed symptoms, however few reported seeking herbal/spiritual help initially.<br><br>Definitive diagnoses were made at the teaching hospital.
Adesunkanmi *et al. 2006*[21]	**Nigeria**	**Breast**	The severity, outcome and challenges of breast cancer in Nigeria	A retrospective study using 8 years records of patient with breast cancer diagnosis in a tertiary health centre.	To determine the challenges of breast cancer diagnosis at the centre.	**Participants:** 212 patients<br>**Sex:** 99.5% female<br>**Mean (SD) age:** 48±12.3 years.<br>**Occupation:** Traders (52%), Teachers (31.6%), Nurses (5.5%), Farmers (4%), and Self-employed (1.4%).<br>**Education:** Primary (18%), Secondary (14%) and Tertiary (35%).<br>Previous breast disease (25%) Family history of breast cancer (7.2%)	○ 92% of the tumour were self-detected<br>○ 4.2% by physicians<br>○ and 3.8% by partners<br>○ Definitive diagnoses of all cases were made at the tertiary health centre

For peer review only - http://bmjopen.bmj.com/site/about/guidelines.xhtml

Ahmed *et al.* 2012[22]	**Nigeria**	**Breast**	Management and Outcomes of Male Breast Cancer in Zaria, Nigeria	A retrospective study using 10 years medical records of men with breast cancer in a specialist oncology centre (in a teaching hospital).	Route to diagnosis	**Participants:** 57 men<br>**Mean (SD) age:** 59 ±2.3 years	○ Definitive diagnoses were made at the specialist centre.<br>○ 21% initially consulted traditional healers before presenting to the specialist.<br>○ 49% first presented symptoms to the specialist.
Aziato *et al.* 2015[29]	**Ghana**	**Breast**	Breast Cancer Diagnosis and Factors Influencing Treatment Decisions in Ghana	A qualitative study using face-to-face interviews of breast cancer patients from a Surgical Unit and breast cancer support group.	Route to diagnosis	**Participants:** 12 female<br>**Age range:** 31-60 years<br>**Religion:** All were Christians	Women self-identified breast lesion or accidently during medical examination for other problems.<br>Participants who self-identified breast lesion presented directly to the tertiary health centre where definitive diagnoses were made.
Agbokey *et al. 2019*[30]	**Ghana**	**Breast**	Knowledge and Health Seeking Behaviour of Breast Cancer Patients in Ghana	A qualitative study using in-depth interviews to examine help-seeking behaviour of female breast cancer patients at a teaching hospital	Patient help-seeking behaviour after noticing female breast symptoms	**Participants:** 20 females<br>**Median (range) age:** 52.5 (29-80) years<br>**Occupation:** Traders (n=11), Teachers (n=3) Farmers (n=5) and Nurse (n=1).<br>**Education: none (n=4)** Primary (n=12), Secondary (n=1), and Tertiary (n=3).<br>**Religion:** 95% were Christians	12/20 first sought unorthodox care (herbalist, drug stores, home remedies and prayer camps) after noticing symptoms. Some patient went through a cycle of hospital-to-herbalist-and back to hospital care before diagnosis.<br>In all of the cases, however, definitive diagnoses were made at the tertiary health centre
Mlange *et al. 2016*[34]	**Tanzania**	**Cervical**	Patient and disease characteristics associated with late tumour stage at presentation of cervical cancer in north-western Tanzania	A cross-sectional survey of women with histologically confirmed cervical cancer at a tertiary health centre	Route to diagnosis	**Participants:** 202 women with cervical cancers<br>**Mean (SD) age:** 50±11 years<br>**Education:** None (57%) Primary (39%), Secondary (2.4%), and College (1.4%).<br>**Occupation:** Farmers (84%), Trader (9.9%), Employed (2.4%), Business (0.9%) and Unemployed (2.4%)	○ 95 (47%) initially presented to traditional health practitioner.<br>○ Presenting to a traditional health practitioner was strongly associated with late-stage diagnosis (Odds Ratio = 2.3 [95% CI 1.2–4.2], p = 0.011)<br>○ Definitive diagnosis were made at the tertiary health centre.
Begoihn *et al.* 2019[28]	**Ethiopia**	**Cervical**	Cervical cancer in Ethiopia – predictors of advanced stage and prolonged time to diagnosis	A retrospective cohort study of patients diagnosed with primary cervical cancer at a tertiary health centre	Route to diagnosis of cervical cancer	**Participants:** 1,575 women with cervical cancers<br>**Mean (SD) age:** 48.9±11.5 years	○ All of the 1,575 women presented cervical cancer symptoms to the tertiary health centre.
Eze *et al.* 2013[23]	**Nigeria**	**Cervical**	A Six-Year Study of the Clinical Presentation of Cervical Cancer and the Management Challenges Encountered at a State Teaching Hospital in Southeast Nigeria	A retrospective cohort study of patients diagnosed with primary cervical cancer at a tertiary health centre	Route to diagnosis of cervical	**Participants:** 61 women with primary cervical cancers. **Mean (SD) age:** 54±12.7 years. **Education:** None (36.1%) Primary (39.3%), Secondary (23%), and College (1.6%).<br>**Occupation:** Farmers (60%), Trader (37.7%), Dependent (36.1%), Business (0.9%) and Retired (6.6%)	○ All of the 61 women presented cervical cancer symptoms to the tertiary health centre.
Alatise *et al.* 2017[24]	**Nigeria**	**Colorectal**	Health-Seeking Behaviour and Barriers to Care in Patients With Rectal Bleeding in Nigeria	A prospective survey of patients with rectal bleeding in the general population.	Attitude about seeking expert opinion among patient with rectal bleeding.<br><br>Initial help-seeking after the onset of rectal bleeding	**Participants:** 82 patients with rectal bleeding<br>**Median (range) age:** 45 (18-85) years<br>**Sex:** 78% were males<br>**Education:** Primary (28%), Secondary (33%), tertiary (30%)<br>**Religion:** Christians (66%) and Muslims (33%)	○ 39% of the participants consulted a physician with rectal bleeding.<br>○ 38% suggested that herbs should be used before seeing a physician.<br>○ Patients who scored high on knowledge of rectal bleeding were more likely to consult the physician (Odds ratio: 3.82; 95% CI, 55-10.2).

For peer review only - http://bmjopen.bmj.com/site/about/guidelines.xhtml

Chu *et al.* 2010[31]	**South Africa**	**Kaposi's Sarcoma**	AIDS-associated Kaposi's sarcoma is linked to advanced disease and high mortality in a primary care HIV programme in South Africa	Analysis of data from a cohort study of patients with AIDS-associated Kaposi's sarcoma in primary care	Patient pathway to diagnosis of Kaposi's Sarcoma	**Participants:** 215 patients with Kaposi's Sarcoma<br>**Median (interquartile range) age:** 34 (29 -41) years<br>**Sex:** 41% were females	189/6292 patients enrolled at the HIV Clinic were diagnosed with AIDS-associated Kaposi's sarcoma during routine examination.
Freeman *et al.* 2016[36]	**Kenya Uganda Malawi Nigeria Cameroon**	**Kaposi's Sarcoma**	Pitfalls of practicing cancer epidemiology in resource-limited settings: the case of survival and loss to follow-up after a diagnosis of Kaposi's sarcoma in five countries across sub-Saharan Africa	Analysis of HIV-infected patients' in primary care records across five countries.	Route to diagnosis of Kaposi's Sarcoma	**Participants:** 1,328 patients with Kaposi's Sarcoma **Median (interquartile range) age:** 35 (30 -41) years<br>**Sex:** 40% were females	During routine examination for AIDS-related infections at the HIV clinic, 1,328 patients were diagnosed with Kaposi's sarcoma across the five countries between 2009 and 2012
Afungchwi *et al.* 2017[33]	**Cameroon**	**Burkitt Lymphoma**	The role of traditional healers in the diagnosis and management of Burkitt lymphoma in Cameroon: understanding the challenges and moving forward	A survey of parents and carers of children diagnosed with Burtkitt lymphoma in three large hospitals	Route to diagnosis Burkitt Lymphoma	**Participants:** 384 questionnaire completed<br>**Median (range) age:** 8 (1 -15) years.<br>**Sex:** Males (57.4%) and females (42.4%)<br>**Religion:** Christians (68.9%) and Muslims (30%)	○ Overall, 55% of parents used traditional healers before hospital admission<br>○ 41.8% first consulted traditional healers before reporting at local health centre.
Antel *et al.* 2019[32]	**South Africa**	**Lymphoma**	The determinants and impact of diagnostic delay in lymphoma in a TB and HIV endemic setting	A retrospective cohort study of patients diagnosed with lymphomas. Data sources included hospital records, telephone and face-to-face interviews.	Route to diagnosis of Hodgkin and Non-Hodgkin lymphoma	**Participants:** 163 HIV patients<br>**Median (range) age:** 48 (15-86) years<br>**Sex:** 58% were males<br>**Socioeconomic status:** 70% on social grant or <251 monthly income	All 163 HIV patients were diagnosed with Hodgkin (41) and Non-Hodgkin (122) lymphoma. They were referred to the tertiary health centre by healthcare practitioners
Brown *et al.* 2015[25]	**Nigeria**	**Childhood**	A Prospective Study on the Causes of Delayed Diagnosis of Childhood Cancer in Ibadan, Nigeria	A survey of parents and carers of children diagnosed with malignant tumour in a tertiary health care settings	Factors influencing pre-diagnostic intervals among parent/carers of patient with childhood cancers	**Participants:** 91 children with cancer<br>**Median (range) age:** 4years (1month -15years).<br>**Sex:** 50.5% were males	○ 69% of parent initially sought medical help for their children within a health facility<br>○ 19% self-medicated<br>○ 4% used herbalist,<br>○ 3% consulted a patent medicine dealers<br>○ 2% presented to a nurse/health worker and 1% visited a church.<br>Health facilities users comprised: 69% Public hospital, 31% Private
Njuguna *et al.* 2016[35]	**Kenya**	**Childhood**	Factors influencing time to diagnosis and treatment among paediatric oncology patients in Kenya	A cross-sectional survey of parent and carers of 99 children diagnosed with a malignancy.	Help-seeking after the onset of symptoms.	**Participants:** 99 children with cancer<br>**Median age:**<br>Children: 5.7years<br>Mother: 31 (19-56) years.<br>**Sex:** 67% were male.<br>**Religion:** 99% of mothers were Christians.<br>**Employment:** Farmers (29%), *Regular jobs (24%),* Casual labourers (6%) and Unemployed (6%)	58 (59%) of parent initially sought alternative treatment for their children, including: praying ceremonies (41%), visiting herbalist (36%), special food intake (11%), and attending traditional healer (3%).<br>First contact with conventional health care facilities included: 60% in primary care, 38% in secondary, and 2% in tertiary health care.

Note: SD Standard Deviation; CI Confidence Interval

For peer review only

For peer review only - http://bmjopen.bmj.com/site/about/guidelines.xhtml

Table 3: Quality of studies

**Study quality and score based on NOS for cohort studies**									
	**Selection**				**Comparability**	***Outcomes and associated statistical analysis***			
**Author, cohort studies**	*Representativeness of the exposed cohort* (★)	*Selection of the non-exposed cohort* (★)	*Ascertainment of exposure* (★)	*Demonstration that outcome of interest was not present at start of study* (★)	*Comparability of cohorts on the basis of the design or analysis* (★★)	*Assessment of outcome* (★)	*Was follow-up long enough for outcomes to occur* (★)	*Adequacy of follow up of cohorts* (★)	*overall quality*
Ahmed *et al.*,2012[22]	★	-	-	★	★	★	★	-	Sat
Adesunkanmi *et al., 2006*[21]	★	-	★	★	-	★	★	-	Poor
Begoihn *et al.,* 2019[28]	★	-	★	-★	★	★	-★	-	Sat
Chu *et al., 2010*[31]	★	-	★	★	★	★	★	-	Sat
Freeman *et al.*,2016[36]	★	-	★	★	★	★	★	-	Sat
Eze *et al.,* 2013[23]	★	-	★	★	-	★	-	-	Poor
Antel *et al.,* 2019[32]	★	-	★	★	★	★	★	-	Sat

**Study quality and score based on NOS adapted for cross-sectional studies**								
**Author, cross-sectional studies**	*Representativeness of the sample* (★)	*Justification of sample size* (★)	*Non-respondents* (★)	*Ascertainment of exposure* (★★)	*Comparability of subjects* (★★)	*Assessment of outcome* (★★)	*Statistical test* (★)	*Overall quality*
Jemebere, 2019[27]	★	-	★	★	-	★	-	Poor
Ezeome *et al.,* 2010[19]	★	-	-	★	-	★	-	Poor
Mlange *et al., 2016*[34]	★	-	★	★	★	★	★	Sat
Alatise *et* al., 2017[24]	★	-	-	★	-	★	-	Poor
Afungchwi *et al.,* 2017[33]	★	-	-	★	-	★	-	Poor
Brown *et al.,* 2015[25]	★	-	-	★	★	-	-	Poor
Njuguna *et al.,* 2016[35]	★	-	★	★	★	★	-	Sat

**Study quality and scores based on the JBI Checklist for Qualitative Research**									
Authors, qualitative studies	*Congruity between stated philosophical perspective and the research methodology?*	*Congruity between research method and the research question or objectives?*	*Congruity between research method and the methods used to collect data?*	*Congruity between research method and representation and analysis of data?*	*Congruity between method & interpretation of results?*	*Statement locating the researcher culturally or theoretically?*	*Influence of the researcher on the research, and vice- versa, addressed?*	*Participants' voices adequately represented?*	*Evidence of ethical approval?*	*Conclusions report flow from the analysis, or interpretation, of the data?*
Dye *et al.* 2010[26]	Unclear	Yes	Yes	Yes	Yes	Unclear	No	Yes	Yes	Yes
Pruitt *et al. 2015*[20]	Unclear	Yes	Yes	Yes	Yes	Unclear	No	Yes	Yes	Yes
Aziato *et al.* 2015[29]	Unclear	Yes	Yes	Yes	Yes	Unclear	No	Yes	Yes	Yes
Agbokey *et al. 2019*[30]	Unclear	Yes	Unclear	Yes	Yes	Unclear	No	Yes	Yes	Yes

Note: Sat satisfactory-quality paper

For peer review only - http://bmjopen.bmj.com/site/about/guidelines.xhtml

Competing interests: The authors declare that they have no competing interests.

Authors' contributions: TM was involved in all aspects. WH participated in the study design, data interpretation and preparation and revision of the manuscript. SM participated in the assessment of studies quality and revision of the manuscript. All authors read and approved the final manuscript.

Ethical approval: Not applicable.

Acknowledgements: Not applicable

Funding: TM receives funding from Cancer Research UK (CRUK) Population Research Committee [C56361/A26124]. SM is supported by the CanTest Collaborative, which is funded by Cancer Research UK [C8640/A23385]. WH is co-Directors of CanTest.

Data availability statement: No additional data available

Figure 1: Flow chart of study selection process

For peer review only - http://bmjopen.bmj.com/site/about/guidelines.xhtml

References

1. Gouda HN, Charlson F, Sorsdahl K, et al. Burden of non-communicable diseases in sub-Saharan Africa, 1990–2017: results from the Global Burden of Disease Study 2017. *The Lancet Global Health* 2019;7(10):e1375-e87. doi: 10.1016/S2214-109X(19)30374-2
2. The World Bank. Disease and Mortality in Sub-Saharan Africa. Washington (DC): The International Bank for Reconstruction and Development/The World Bank. 2006.
3. Azubuike SO, Muirhead C, Hayes L, et al. Rising global burden of breast cancer: the case of sub-Saharan Africa (with emphasis on Nigeria) and implications for regional development: a review. *World J Surg Oncol* 2018;16(1):63. doi: 10.1186/s12957-018-1345-2
4. Musgrove DTJJGBARMGAMCDBEPJAMP. Disease Control Priorities in Developing Countries (Second Edition).
5. Brawley OW. The role of government and regulation in cancer prevention. *The Lancet Oncology* 2017;18(8):e483-e93. doi: https://doi.org/10.1016/S1470-2045(17)30374-1
6. Plummer M, de Martel C, Vignat J, et al. Global burden of cancers attributable to infections in 2012: a synthetic analysis. *The Lancet Global Health* 2016;4(9):e609-e16. doi: 10.1016/S2214-109X(16)30143-7
7. McPhail S, Johnson S, Greenberg D, et al. Stage at diagnosis and early mortality from cancer in England. *Br J Cancer* 2015;112 Suppl 1(Suppl 1):S108-S15. doi: 10.1038/bjc.2015.49
8. Jedy-Agba E, McCormack V, Adebamowo C, et al. Stage at diagnosis of breast cancer in sub-Saharan Africa: a systematic review and meta-analysis. *The Lancet Global Health* 2016;4(12):e923-e35. doi: https://doi.org/10.1016/S2214-109X(16)30259-5
9. Olaleye O, Ekrikpo U. Epidemiology of Cancers in Sub-Saharan Africa. In: Adedeji OA, ed. Cancer in Sub-Saharan Africa: Current Practice and Future. Cham: Springer International Publishing 2017:3-19.
10. Cassim S, Chepulis L, Keenan R, et al. Patient and carer perceived barriers to early presentation and diagnosis of lung cancer: a systematic review. *BMC Cancer* 2019;19(1):25. doi: 10.1186/s12885-018-5169-9 [published Online First: 2019/01/10]
11. Scott SE, Walter FM, Webster A, et al. The Model of Pathways to Treatment: Conceptualization and integration with existing theory. *British Journal of Health Psychology* 2013;18(1):45-65. doi: 10.1111/j.2044-8287.2012.02077.x
12. Khakbazan Z, Taghipour A, Latifnejad Roudsari R, et al. Help seeking behavior of women with self-discovered breast cancer symptoms: a meta-ethnographic synthesis of patient delay. *PLoS ONE* 2014;9(12):e110262-e62. doi: 10.1371/journal.pone.0110262
13. Moher D, Shamseer L, Clarke M, et al. Preferred reporting items for systematic review and meta-analysis protocols (PRISMA-P) 2015 statement. *Systematic Reviews* 2015;4(1):1. doi: 10.1186/2046-4053-4-1
14. The World Bank. Data Catalog: Regions and Countries 2019 [Available from: https://datacatalog.worldbank.org/ accessed 10/10 2019.
15. Rodgers M, Sowden A, Petticrew M, et al. Testing methodological guidance on the conduct of narrative synthesis in systematic reviews: effectiveness of interventions to promote smoke alarm ownership and function. *Evaluation* 2009;15(1):49-73.
16. GA Wells BS, D O'Connell, J Peterson, V Welch, M Losos, P Tugwell,. The Newcastle-Ottawa Scale (NOS) for assessing the quality of nonrandomised studies in meta-analyses [03/08/2020]. Available from: http://www.ohri.ca/programs/clinical_epidemiology/oxford.asp accessed 22/07/2020.
17. Modesti PA, Reboldi G, Cappuccio FP, et al. Panethnic Differences in Blood Pressure in Europe: A Systematic Review and Meta-Analysis. *PLoS ONE* 2016;11(1):e0147601. doi: 10.1371/journal.pone.0147601
18. Lockwood C, Munn Z, Porritt K. Qualitative research synthesis: methodological guidance for systematic reviewers utilizing meta-aggregation. *Int J Evid Based Healthc* 2015;13(3):179-87. doi: 10.1097/xeb.0000000000000062 [published Online First: 2015/08/12]

For peer review only - http://bmjopen.bmj.com/site/about/guidelines.xhtml

19. Ezeome ER. Delays in presentation and treatment of breast cancer in Enugu, Nigeria. *Niger J Clin Pract* 2010;13(3):311-6.
20. Pruitt L, Mumuni T, Raikhel E, et al. Social barriers to diagnosis and treatment of breast cancer in patients presenting at a teaching hospital in Ibadan, Nigeria. *Glob Public Health* 2015;10(3):331-44. doi: https://dx.doi.org/10.1080/17441692.2014.974649
21. Adesunkanmi AR, Lawal OO, Adelusola KA, et al. The severity, outcome and challenges of breast cancer in Nigeria. *Breast* 2006;15(3):399-409. doi: 10.1016/j.breast.2005.06.008 [published Online First: 2005/08/09]
22. Ahmed A, Ukwenya Y, Abdullahi A, et al. Management and Outcomes of Male Breast Cancer in Zaria, Nigeria. *Int J Breast Cancer* 2012;2012:6. doi: 10.1155/2012/845143
23. Eze JN, Emeka-Irem EN, Edegbe FO. A Six-Year Study of the Clinical Presentation of Cervical Cancer and the Management Challenges Encountered at a State Teaching Hospital in Southeast Nigeria. *Clinical Medicine Insights: Oncology* 2013(7):151-58. doi: 10.4137/CMO.S12017
24. Alatise OI, Fischer SE, Ayandipo OO, et al. Health-Seeking Behavior and Barriers to Care in Patients With Rectal Bleeding in Nigeria. *J Glob Oncol* 2017;3(6):749-56. doi: https://dx.doi.org/10.1200/JGO.2016.006601
25. Brown BJ, Adeleye AO, Ibeh JN. A Prospective Study on the Causes of Delayed Diagnosis of Childhood Cancer in Ibadan, Nigeria. *Pediatr Hematol Oncol* 2015;32(6):365-73. doi: https://dx.doi.org/10.3109/08880018.2015.1040933
26. Dye TD, Bogale S, Hobden C, et al. Complex care systems in developing countries: breast cancer patient navigation in Ethiopia. *Cancer* 2010;116(3):577-85. doi: https://dx.doi.org/10.1002/cncr.24776
27. Jemebere W. Barriers Associated with Presentation Delay among Breast Cancer Patients at Hawassa University Comprehensive and Specialized Hospital, Southern Ethiopia. *International Journal of Caring Sciences* 2019;12(3):1356-63.
28. Begoihn M, Mathewos A, Aynalem A, et al. Cervical cancer in Ethiopia – predictors of advanced stage and prolonged time to diagnosis. *Infectious Agents and Cancer* 2019;14(1):36. doi: 10.1186/s13027-019-0255-4
29. Aziato L, Clegg-Lamptey JNA. Breast Cancer Diagnosis and Factors Influencing Treatment Decisions in Ghana. *Health Care Women Int* 2015;36(5):543-57. doi: 10.1080/07399332.2014.911299
30. Agbokey F, Kudzawu E, Dzodzomenyo M, et al. Knowledge and Health Seeking Behaviour of Breast Cancer Patients in Ghana. *Int J Breast Cancer* 2019;2019:5239840. doi: 10.1155/2019/5239840
31. Chu KM, Mahlangeni G, Swannet S, et al. AIDS-associated Kaposi's sarcoma is linked to advanced disease and high mortality in a primary care HIV programme in South Africa. *J Int AIDS Soc* 2010;13:23. doi: https://dx.doi.org/10.1186/1758-2652-13-23
32. Antel K, Levetan C, Mohamed Z, et al. The determinants and impact of diagnostic delay in lymphoma in a TB and HIV endemic setting. *BMC Cancer* 2019;19(1):384. doi: 10.1186/s12885-019-5586-4 [published Online First: 2019/04/27]
33. Afungchwi GM, Hesseling PB, Ladas EJ. The role of traditional healers in the diagnosis and management of Burkitt lymphoma in Cameroon: understanding the challenges and moving forward. *BMC Complementary & Alternative Medicine* 2017;17:1-7. doi: 10.1186/s12906-017-1719-y
34. Mlange R, Matovelo D, Rambau P, et al. Patient and disease characteristics associated with late tumour stage at presentation of cervical cancer in northwestern Tanzania. *BMC Womens Health* 2016;16(5):1-6. doi: 10.1186/s12905-016-0285-7
35. Njuguna F, Martijn H, Langat S, et al. Factors influencing time to diagnosis and treatment among pediatric oncology patients in Kenya. *Pediatr Hematol Oncol* 2016;33(3):186-99. doi: https://dx.doi.org/10.3109/08880018.2016.1169566

For peer review only - http://bmjopen.bmj.com/site/about/guidelines.xhtml

36. Freeman E, Semeere A, Wenger M, et al. Pitfalls of practicing cancer epidemiology in resource-limited settings: the case of survival and loss to follow-up after a diagnosis of Kaposi's sarcoma in five countries across sub-Saharan Africa. *BMC Cancer* 2016;16:65. doi: https://dx.doi.org/10.1186/s12885-016-2080-0
37. Elliss-Brookes L, McPhail S, Ives A, et al. Routes to diagnosis for cancer - determining the patient journey using multiple routine data sets. *Br J Cancer* 2012;107(8):1220-26. doi: 10.1038/bjc.2012.408 [published Online First: 2012/09/20]
38. Palser TR, Cromwell DA, Hardwick RH, et al. Impact of route to diagnosis on treatment intent and 1-year survival in patients diagnosed with oesophagogastric cancer in England: a prospective cohort study. *BMJ Open* 2013;3(2):e002129. doi: 10.1136/bmjopen-2012-002129
39. Hamilton W. Five misconceptions in cancer diagnosis. *Br J Gen Pract* 2009;59(563):441-47.
40. Stefan DC. Cancer Care in Africa: An Overview of Resources. *J Glob Oncol* 2015;1(1):30-36. doi: 10.1200/JGO.2015.000406
41. James PB, Wardle J, Steel A, et al. Traditional, complementary and alternative medicine use in Sub-Saharan Africa: a systematic review. *BMJ Global Health* 2018;3(5):e000895. doi: 10.1136/bmjgh-2018-000895
42. Kingham TP, Alatise OI, Vanderpuye V, et al. Treatment of cancer in sub-Saharan Africa. *Lancet Oncol* 2013;14(4):e158-67. doi: 10.1016/s1470-2045(12)70472-2 [published Online First: 2013/04/09]
43. Mash R, Howe A, Olayemi O, et al. Reflections on family medicine and primary healthcare in sub-Saharan Africa. *BMJ Global Health* 2018;3(Suppl 3):e000662. doi: 10.1136/bmjgh-2017-000662
44. A OA, Taiwo O, S AO, et al. Breast cancer awareness, attitude and screening practices in Nigeria: A systematic review. *Clinical Reviews and Opinions* 2016;7(2):11-25. doi: 10.5897/cro16.0101
45. McKenzie F, Zietsman A, Galukande M, et al. Breast cancer awareness in the sub-Saharan African ABC-DO cohort: African Breast Cancer—Disparities in Outcomes study. *Cancer Causes Control* 2018;29(8):721-30. doi: 10.1007/s10552-018-1047-7
46. Economist Intelligence Unit. The future of healthcare in Africa. 2012 [Available from: https://perspectives.eiu.com/sites/default/files/EIU-Janssen_HealthcareAfrica_Report_Web.pdf accessed 27/11 2019.
47. Campbell J DG, Buchan J, Pozo-Martin F, Guerra Arias M, Leone C, Siyam A, Cometto G.,. The future of healthcare in Africa,. Forum Report, Third Global Forum on Human Resources for Health, Recife, Brazil Geneva, Global Health Workforce Alliance and World Health Organization, 2013.
48. Johnson SB, Park HS, Gross CP, et al. Use of Alternative Medicine for Cancer and Its Impact on Survival. *JNCI: Journal of the National Cancer Institute* 2017;110(1):121-24. doi: 10.1093/jnci/djx145

For peer review only - http://bmjopen.bmj.com/site/about/guidelines.xhtml

Figure 1: Flow chart of study selection process

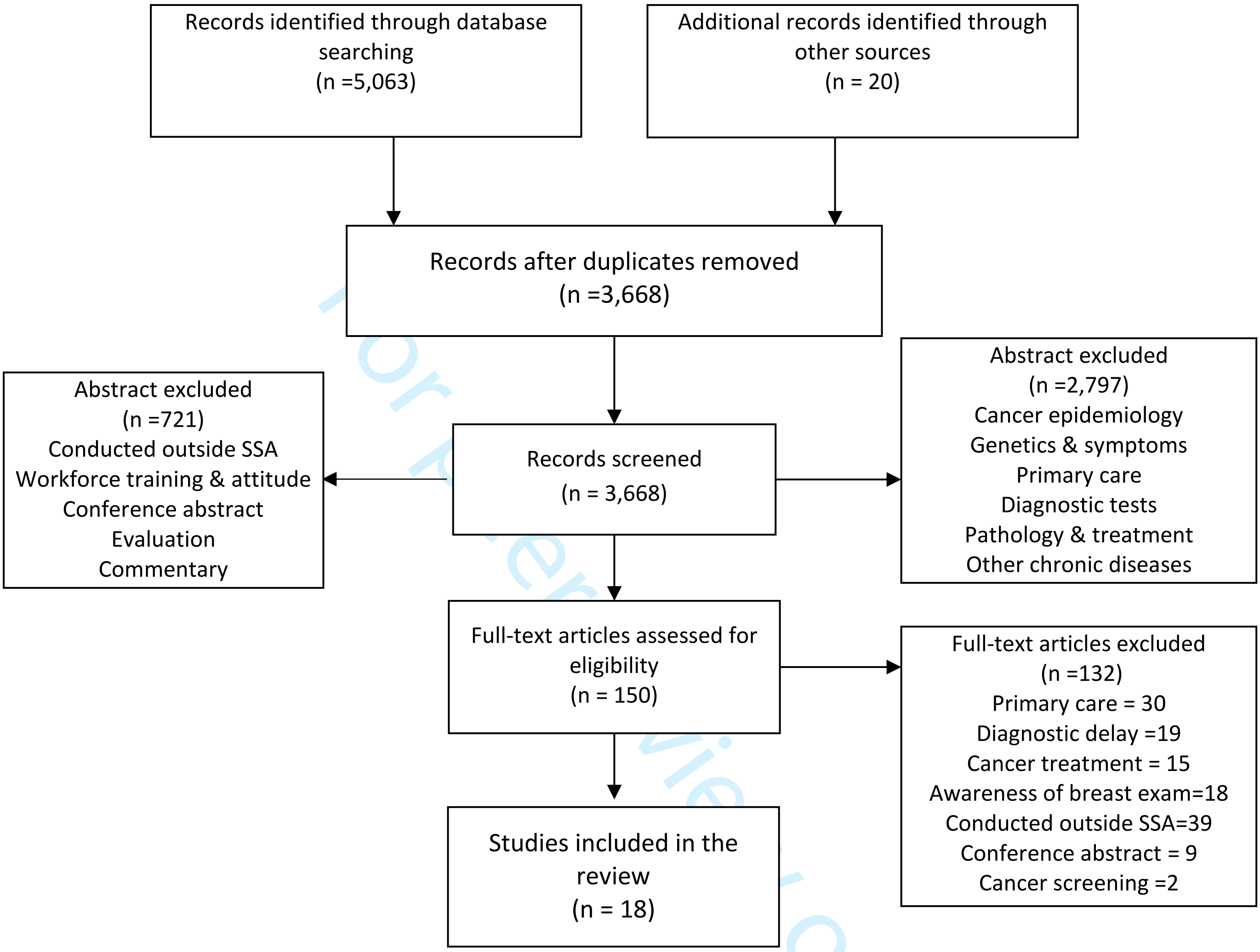

For peer review only

PRISMA 2009 Checklist

Section/topic	#	Checklist item	Reported on page #
**TITLE**			
Title	1	Identify the report as a systematic review, meta-analysis, or both.	1
**ABSTRACT**			
Structured summary	2	Provide a structured summary including, as applicable: background; objectives; data sources; study eligibility criteria, participants, and interventions; study appraisal and synthesis methods; results; limitations; conclusions and implications of key findings; systematic review registration number.	2
**INTRODUCTION**			
Rationale	3	Describe the rationale for the review in the context of what is already known.	4
Objectives	4	Provide an explicit statement of questions being addressed with reference to participants, interventions, comparisons, outcomes, and study design (PICOS).	5
**METHODS**			
Protocol and registration	5	Indicate if a review protocol exists, if and where it can be accessed (e.g., Web address), and, if available, provide registration information including registration number.	N/A
Eligibility criteria	6	Specify study characteristics (e.g., PICOS, length of follow-up) and report characteristics (e.g., years considered, language, publication status) used as criteria for eligibility, giving rationale.	5
Information sources	7	Describe all information sources (e.g., databases with dates of coverage, contact with study authors to identify additional studies) in the search and date last searched.	5
Search	8	Present full electronic search strategy for at least one database, including any limits used, such that it could be repeated.	Additional file 2
Study selection	9	State the process for selecting studies (i.e., screening, eligibility, included in systematic review, and, if applicable, included in the meta-analysis).	5-7
Data collection process	10	Describe method of data extraction from reports (e.g., piloted forms, independently, in duplicate) and any processes for obtaining and confirming data from investigators.	6
Data items	11	List and define all variables for which data were sought (e.g., PICOS, funding sources) and any assumptions and simplifications made.	6
Risk of bias in individual studies	12	Describe methods used for assessing risk of bias of individual studies (including specification of whether this was done at the study or outcome level), and how this information is to be used in any data synthesis.	6-7
Summary measures	13	State the principal summary measures (e.g., risk ratio, difference in means).	N/A
Synthesis of results	14	Describe the methods of handling data and combining results of studies, if done, including measures of consistency (e.g., I^2) for each meta-analysis.	6

For peer review only

For peer review only - http://bmjopen.bmj.com/site/about/guidelines.xhtml

PRISMA 2009 Checklist

Section/topic	#	Checklist item	Reported on page #
Risk of bias across studies	15	Specify any assessment of risk of bias that may affect the cumulative evidence (e.g., publication bias, selective reporting within studies).	6 & 8
Additional analyses	16	Describe methods of additional analyses (e.g., sensitivity or subgroup analyses, meta-regression), if done, indicating which were pre-specified.	N/A
**RESULTS**			
Study selection	17	Give numbers of studies screened, assessed for eligibility, and included in the review, with reasons for exclusions at each stage, ideally with a flow diagram.	7 - 8 and Figure 1
Study characteristics	18	For each study, present characteristics for which data were extracted (e.g., study size, PICOS, follow-up period) and provide the citations.	7-9 and 16-18
Risk of bias within studies	19	Present data on risk of bias of each study and, if available, any outcome level assessment (see item 12).	8 & 19
Results of individual studies	20	For all outcomes considered (benefits or harms), present, for each study: (a) simple summary data for each intervention group (b) effect estimates and confidence intervals, ideally with a forest plot.	7-9 & 16-18
Synthesis of results	21	Present results of each meta-analysis done, including confidence intervals and measures of consistency.	N/A
Risk of bias across studies	22	Present results of any assessment of risk of bias across studies (see Item 15).	8 & 19
Additional analysis	23	Give results of additional analyses, if done (e.g., sensitivity or subgroup analyses, meta-regression [see Item 16]).	N/A
**DISCUSSION**			
Summary of evidence	24	Summarize the main findings including the strength of evidence for each main outcome; consider their relevance to key groups (e.g., healthcare providers, users, and policy makers).	10
Limitations	25	Discuss limitations at study and outcome level (e.g., risk of bias), and at review-level (e.g., incomplete retrieval of identified research, reporting bias).	10
Conclusions	26	Provide a general interpretation of the results in the context of other evidence, and implications for future research.	13
**FUNDING**			
Funding	27	Describe sources of funding for the systematic review and other support (e.g., supply of data); role of funders for the systematic review.	20

From: Moher D, Liberati A, Tetzlaff J, Altman DG, The PRISMA Group (2009). Preferred Reporting Items for Systematic Reviews and Meta-Analyses: The PRISMA Statement. PLoS Med 6(7): e1000097. doi:10.1371/journal.pmed1000097

For more information, visit: www.prisma-statement.org.

For peer review only - http://bmjopen.bmj.com/site/about/guidelines.xhtml

Additional file 2: Ovid MEDLINE(R), PsycINFO, Embase, Global Health

Set	Search Statement
1.	Angola*.ti,ab.
2.	Gabon*.ti,ab.
3.	Nigeria*.ti,ab.
4.	Benin*.ti,ab.
5.	Gambia*.ti,ab.
6.	Rwanda*.ti,ab.
7.	Botswana*.ti,ab.
8.	Ghana*.ti,ab.
9.	Sao Tome*.ti,ab.
10.	Sao Tome.mp. and Principe*.ti,ab. [mp=title, abstract, heading word, drug trade name, original title, device manufacturer, drug manufacturer, device trade name, keyword, floating subheading word, candidate term word]
11.	Burkina Faso*.ti,ab.
12.	Guinea-Bissau*.ti,ab.
13.	Seychelles*.ti,ab.
14.	Guinea*.ti,ab.
15.	Senegal*.ti,ab.
16.	Burundi*.ti,ab.
17.	Cabo Verde*.ti,ab.
18.	Kenya*.ti,ab.
19.	Sierra Leone*.ti,ab.
20.	Cameroon*.ti,ab.
21.	Lesotho*.ti,ab.
22.	Somalia*.ti,ab.
23.	Central African Republic*.ti,ab.
24.	Liberia*.ti,ab.
25.	South Africa*.ti,ab.
26.	Chad*.ti,ab.
27.	Madagascar*.ti,ab.
28.	South Sudan*.ti,ab.
29.	Comoros*.ti,ab.
30.	Malawi*.ti,ab.
31.	Sudan*.ti,ab.
32.	Congo*.ti,ab.
33.	Mali*.ti,ab.
34.	Swaziland*.ti,ab.
35.	Mauritania*.ti,ab.
36.	Tanzania*.ti,ab.
37.	Cote d'Ivoire*.ti,ab.
38.	Mauritius*.ti,ab.
39.	togo*.ti,ab.
40.	Equatorial Guinea*.ti,ab.
41.	Mozambique*.ti,ab.
42.	Uganda*.ti,ab.
43.	Eritrea*.ti,ab.
44.	Namibia*.ti,ab.
45.	Zambia*.ti,ab.
46.	Ethiopia*.ti,ab.
47.	Niger*.ti,ab.
48.	Zimbabwe*.ti,ab.
49.	Africa*.ti,ab.

For peer review only

50.	Sub-Sahara*.ti,ab.
51.	1 or 2 or 3 or 4 or 5 or 6 or 7 or 8 or 9 or 10 or 11 or 12 or 13 or 14 or 15 or 16 or 17 or 18 or 19 or 20 or 21 or 22 or 23 or 24 or 25 or 26 or 27 or 28 or 29 or 30 or 31 or 32 or 33 or 34 or 35 or 36 or 37 or 38 or 39 or 40 or 41 or 42 or 43 or 44 or 45 or 46 or 47 or 48 or 49 or 50
52.	cancer*.ti,ab.
53.	exp Neoplasms/
54.	(adenocarcinoma or astrocytoma or glioma or Hodgkin$ or leuk?emia or lymphoma or medulloblastoma or melanoma or mesothelioma or myeloma or neuroblastoma or nonmelanoma or osteosarcoma or retinoblastoma or sarcoma or seminoma or teratoma).ti,ab.
55.	52 or 53 or 54
56.	(pathway adj5 diagnos$).ti,ab.
57.	(pathway adj5 detect$).ti,ab.
58.	(route adj5 diagnos$).ti,ab.
59.	(route adj5 detect$).ti,ab.
60.	diagnos$.ti,ab.
61.	detect$.ti,ab.
62.	consult$.ti,ab.
63.	(help adj5 seek$).ti,ab.
64.	present$.ti,ab.
65.	(route adj5 consult$).ti,ab.
66.	(route adj5 present$).ti,ab.
67.	(pathway adj5 consult$).ti,ab.
68.	(pathway adj5 present$).ti,ab.
69.	56 or 57 or 58 or 59 or 60 or 61 or 62 or 63 or 64 or 65 or 66 or 67 or 68
70.	primary care.ti,ab.
71.	family doctor.ti,ab.
72.	physician.ti,ab.
73.	(health adj5 practitioner).ti,ab.
74.	General Practitioners/ or Family Practice/ or Primary Health Care/
75.	70 or 71 or 72 or 73 or 74
76.	51 and 55 and 69 and 75

For peer review only
